# cLD: Rare-variant linkage disequilibrium between genomic regions identifies novel genomic interactions

**DOI:** 10.1371/journal.pgen.1011074

**Published:** 2023-12-18

**Authors:** Dinghao Wang, Deshan Perera, Jingni He, Chen Cao, Pathum Kossinna, Qing Li, William Zhang, Xingyi Guo, Alexander Platt, Jingjing Wu, Qingrun Zhang

**Affiliations:** 1 Department of Mathematics and Statistics, University of Calgary, Calgary, Alberta, Canada; 2 Department of Biochemistry and Molecular Biology, University of Calgary, Calgary, Alberta, Canada; 3 The Harker School, San Jose, California, United States of America; 4 Division of Epidemiology, Department of Medicine, Vanderbilt Epidemiology Center, Vanderbilt-Ingram Cancer Center, Vanderbilt University School of Medicine, Nashville, Tennessee, United States of America; 5 Department of Biomedical Informatics, Vanderbilt University School of Medicine, Nashville, Tennessee, United States of America; 6 Department of Genetics, Perelman School of Medicine at the University of Pennsylvania, Philadelphia, Pennsylvania, United States of America; 7 Alberta Children’s Hospital Research Institute, University of Calgary, Calgary, Alberta, Canada; Newcastle University, UNITED KINGDOM

## Abstract

Linkage disequilibrium (LD) is a fundamental concept in genetics; critical for studying genetic associations and molecular evolution. However, LD measurements are only reliable for common genetic variants, leaving low-frequency variants unanalyzed. In this work, we introduce cumulative LD (cLD), a stable statistic that captures the rare-variant LD between genetic regions, which reflects more biological interactions between variants, in addition to lack of recombination. We derived the theoretical variance of cLD using delta methods to demonstrate its higher stability than LD for rare variants. This property is also verified by bootstrapped simulations using real data. In application, we find cLD reveals an increased genetic association between genes in 3D chromatin interactions, a phenomenon recently reported negatively by calculating standard LD between common variants. Additionally, we show that cLD is higher between gene pairs reported in interaction databases, identifies unreported protein-protein interactions, and reveals interacting genes distinguishing case/control samples in association studies.

## Author summary

Linkage disequilibrium (LD) is a crucial concept in genetics, but current methods of measuring LD are not reliable for rare variants. In our study, we introduce a new statistic called cumulative LD (cLD) that captures the linkage disequilibrium between genetic regions containing rare variants, which are often unanalyzed. We demonstrate that cLD is a stable statistic and show through simulations using real data that it is more reliable than traditional LD for rare variants.

Our findings show that cLD is a valuable tool for studying genetic associations and molecular evolution. We use cLD to reveal increased genetic association between genes in 3D chromatin interactions, which standard LD failed to detect. Additionally, we show that cLD identifies unreported protein-protein interactions and distinguishes case/control samples in association studies. Our work is significant because it provides a stable and reliable method for analyzing rare variants, which were previously overlooked due to unreliable LD measurements. Our findings have implications for future genetic association studies and molecular evolution research, particularly in understanding rare variant interactions and their biological significance.

## Introduction

Linkage Disequilibrium (LD) is a fundamental concept in population genetics that statistically captures non-random associations between two genetic variants due to reasons such as lack of recombination or different age of mutations [1]. LD serves as a core component in genotype-phenotype association mapping, as a statistically significant genetic variant could be just a proxy in LD with the genuine causal variant(s) [2]. To this end, LD is critically important in analyzing the fine resolution of genotype-phenotype association mapping [3] and forming polygenic risk scores [4]. Additionally, from the perspective of molecular evolution, LD values substantially higher than expected under neutrality may indicate interesting phenomena, e.g., interactions between loci that are favored by selection [5]. As such, LD has been extensively utilized in evolutionary studies.

The calculation of LD involves the use of allele frequencies of the genetic variants in its denominator to normalize the statistic (**Section 1.1 in S1 Text**) and therefore suffers from a high variance (instability) when allele frequencies are close to zero. As such, in practice, when calculating LD, researchers only use common genetic variants with minor allele frequency (MAF) higher than a threshold (e.g.,0.05), excluding more than 90% of human genetic variants [6].

**Fig 1:**
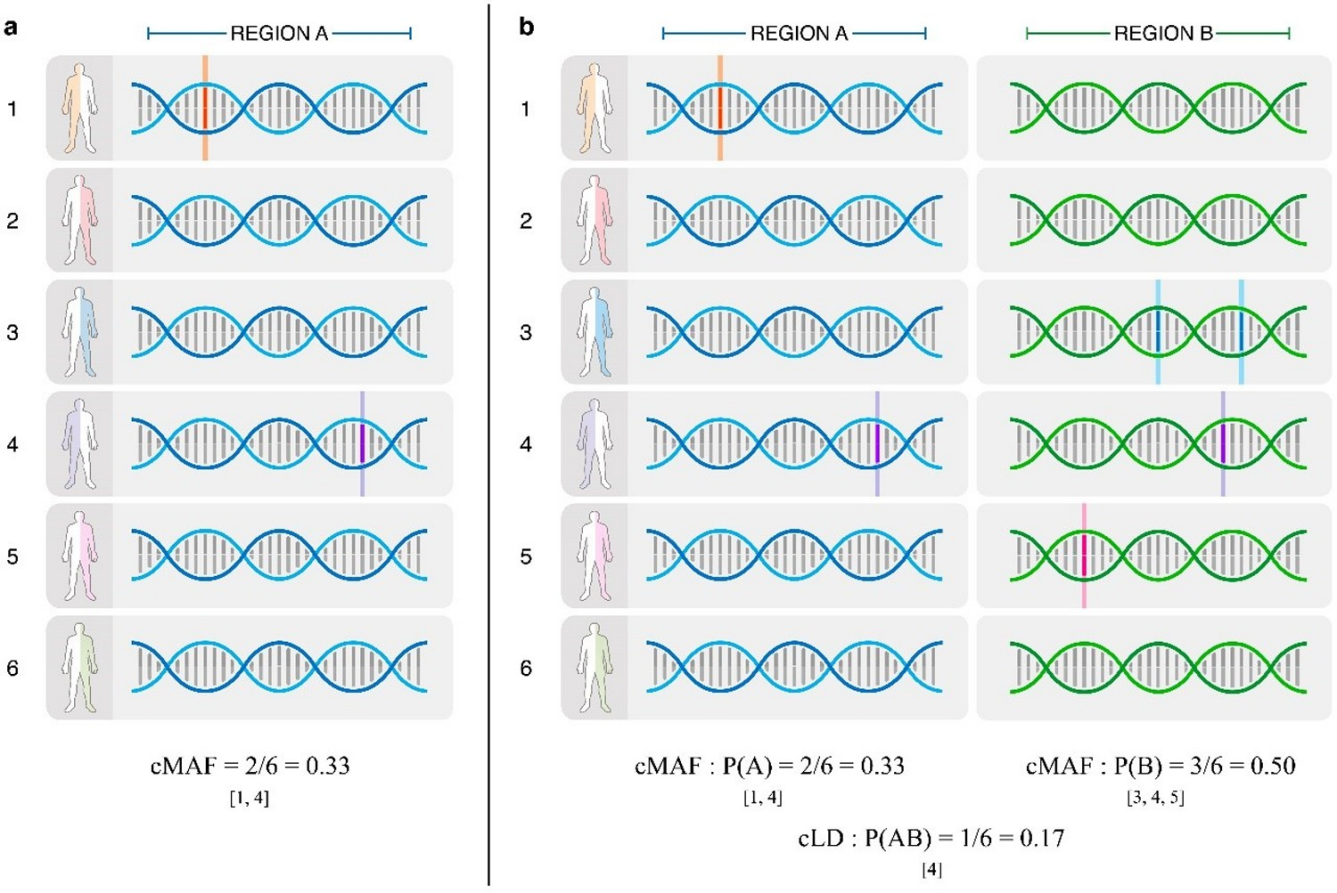
Illustration of the idea of a) cMAF and b) cLD. An example to show the calculation of cLD, inspired by cMAF. **a)** Out of six haplotypes, there are two [1, 4] who have mutations in region A. Therefore, the cMAF P(A) for region A is 2/6 = 0.33. **b)** There are three haplotypes [3, 4, 5] who have mutations in region B and the cMAF P(B) for region B is 3/6 = 0.50. If one considers regions A and B together, there is one individual with mutations in both regions: [4]. Thus, the P(AB) is 1/6 = 0.17. Finally, by yielding P(A), P(B) and P(AB) into the standard formula of LD we have cLD = 0.375. (This is only an illustrating example with 6 haplotypes. In practice, the variants contributing to cMAF or cLD are rare ones with MAF < 0.5%.

In the field of association mapping, researchers have developed multiple techniques to aggregate the associations of multiple rare variants with a phenotype into a single shared effect[7]. One of the pioneering methods that is still popularly used [8] is synthesizing a cumulative allele frequency from multiple rare genetic variants in the same genetic region (e.g., within a gene). The cumulative minor allele frequency (cMAF) is defined on a region containing multiple rare variants: an individual will be labelled as a “mutant” if it has at least one of the rare variants, and then the proportion of individuals in the sample that are labelled as mutants will be the cMAF for this region (**Fig 1A**).

Building on the idea of cMAF and the essence of LD, we developed a statistic, cumulative Linkage Disequilibrium (cLD) to capture the aggregated correlation between two sets of rare variants (**Description of the Methods; Fig 1B**). LD may be defined in three quantities, i.e., *D*,*D*′ and *r*^2^. *D* is just the observed haplotype frequency minus the expected. *D*′ uses the theoretical maximal of *D* to carry out the normalization, and *r*^2^ uses the variance for normalization. Although we defined all of the three quantities for cLD (**Section 1.1 in S1 Text**), the analysis and verification are centralized by *r*^2^ as it is equivalent to standard correlation in statistics, easing the mathematical derivations.

In contrast to the previous attempts to utilize LD between multiple variants focusing on dominant haplotypes [9] or joint distributions [10], cLD emphasizes biological interactions. Additionally, previously researchers have proposed composite linkage [11,12], which addresses the property of variances and its normalization, however, does not incorporate rare variants.

**Fig 2:**
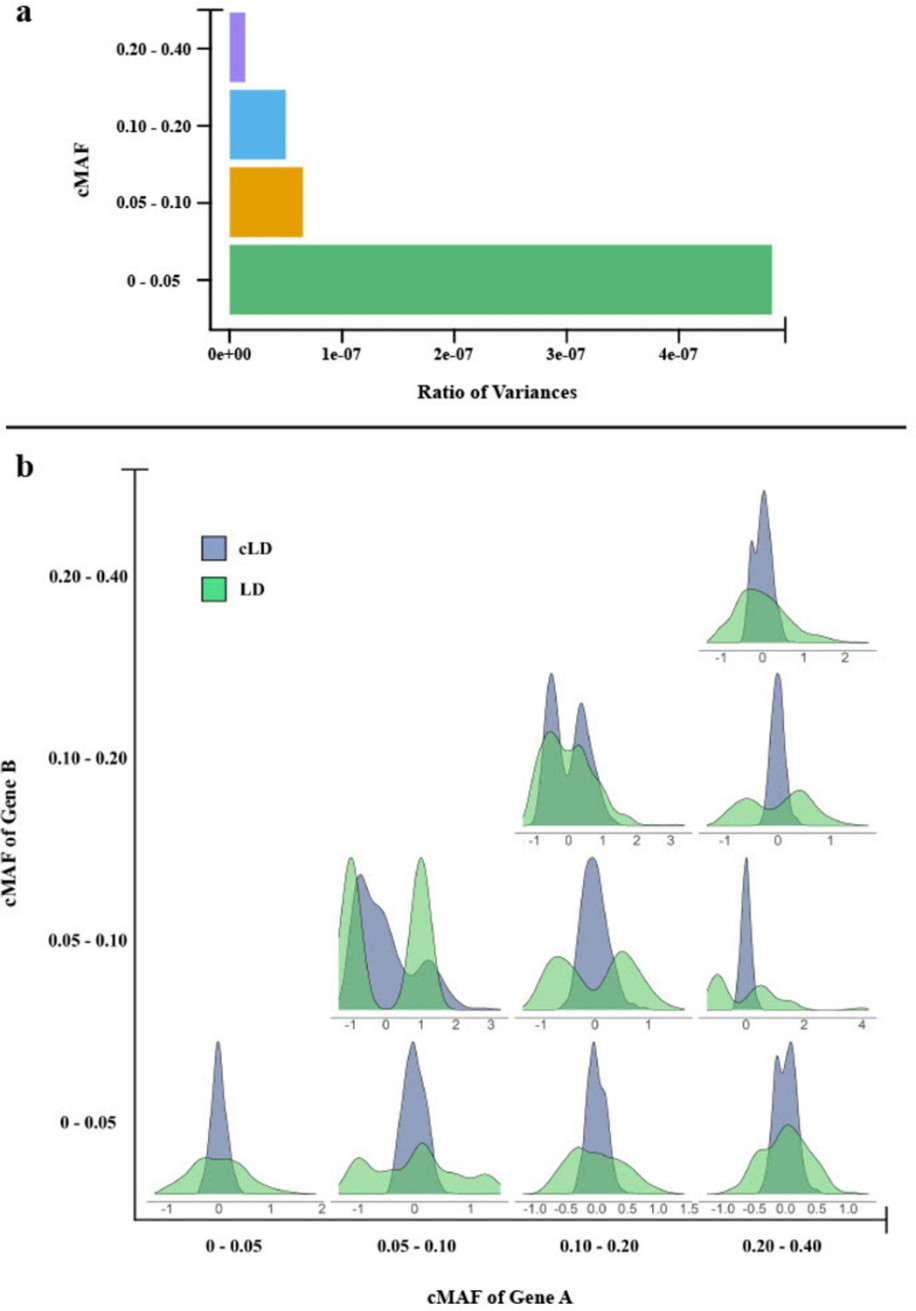
Stability of cLD and LD revealed by closed-form variance calculation and bootstrapped distributions. **a)** The gene pairs were split into four different bins based on the cMAF values, i.e., <0.05, 0.05–0.10, 0.10–0.20, and 0.20–0.40 (y-axis). The x-axis is the ratio between the variances of cLD and LD, i.e., Var(cLD)/Var(LD). **b)** Probability density distribution of cLD and LD from bootstrapped samples. Results from the European population are shown. See **Figs A and B in S1 Text** for other populations.

**Fig 3:**
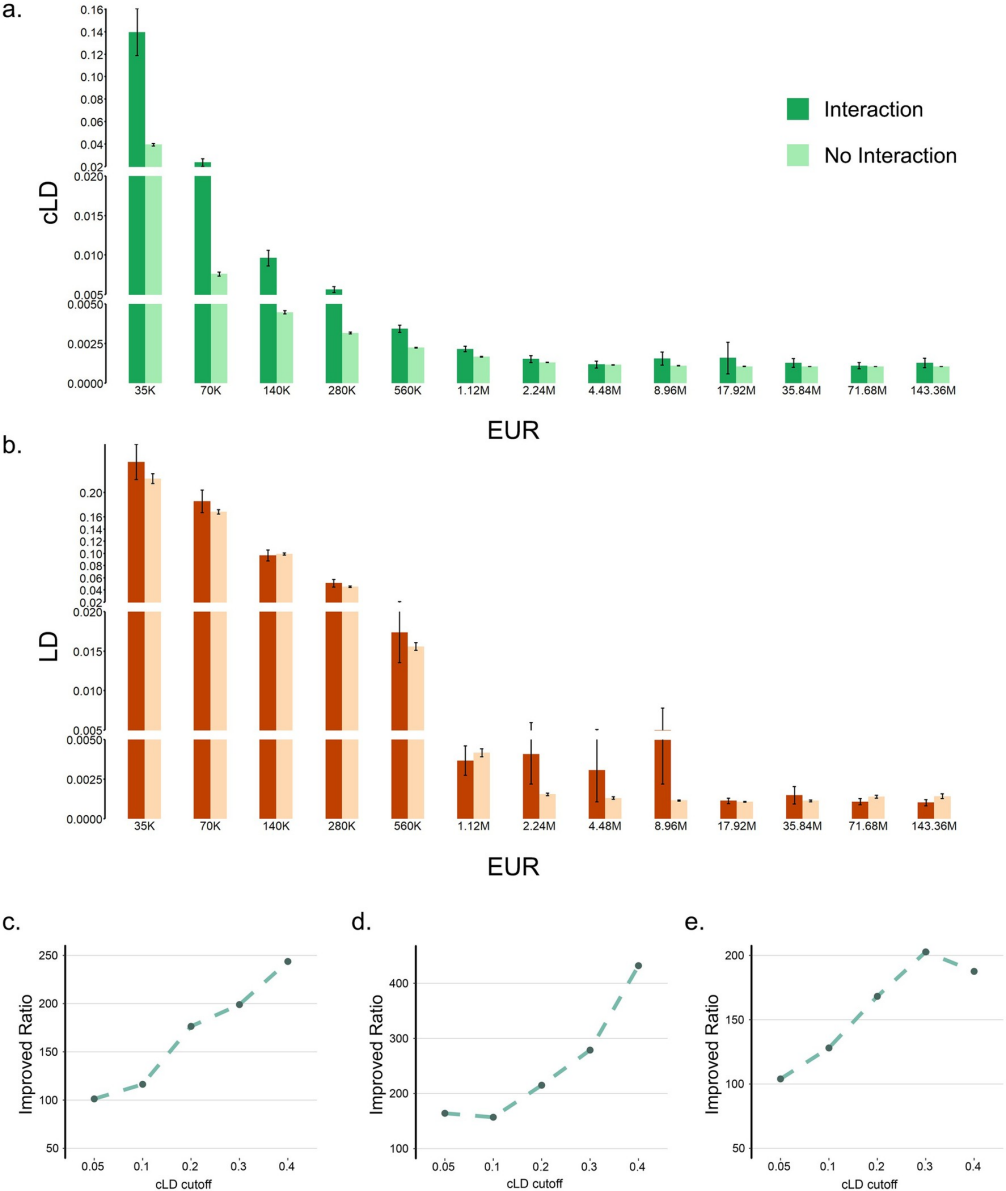
Enrichment of cLD among pairs of genes in chromatin contact regions. **a)** The comparisons of cLD values between the 3D chromatin interaction regions and non-interaction regions among 13 different distance groups in the European population. (Other populations are shown in **Fig D in S1 Text**) The confidence intervals for these bars are presented in **Table I in S1 Text**. **b)** The same comparisons using standard LD in the European population. (Other populations are shown in **Fig E in S1 Text**) **c-e)** The ratios between the number of gene pairs in 3D chromatin interaction regions against the number of gene pairs that are not in 3D regions. The x-axis is the cLD value cutoffs above which the gene pairs are counted. **c)** European population. **d)** African population. **e)** East Asian population.

**Fig 4:**
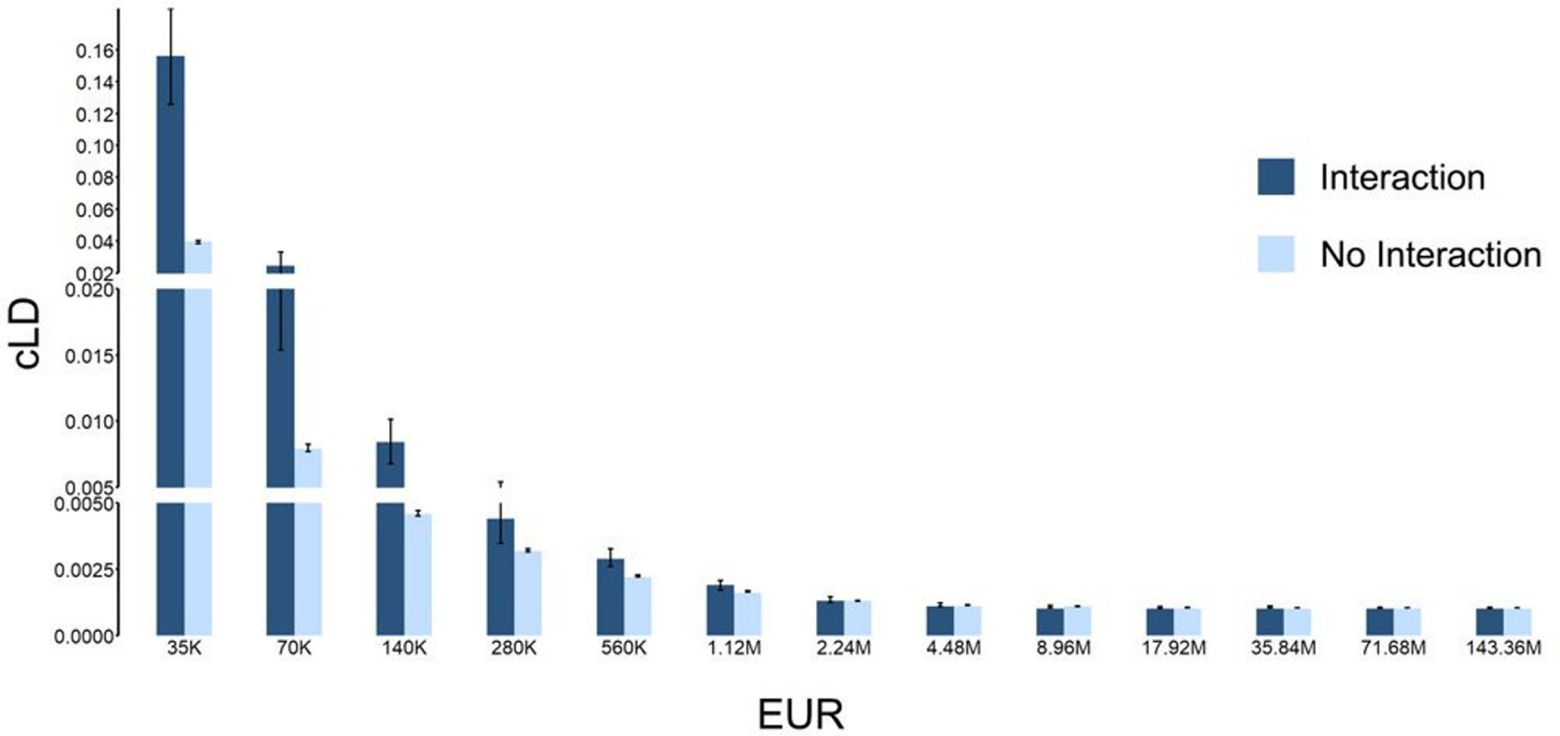
The comparisons of average cLD values in European populations between gene pairs found in interaction databases and all pairs that are not in databases. Each bar represents the average of pairs with distance smaller than the value of its x-axis label but larger than the value of the previous x-axis label. (Other populations show the same trend, as depicted in **Fig F in S1 Text**).

**Fig 5:**
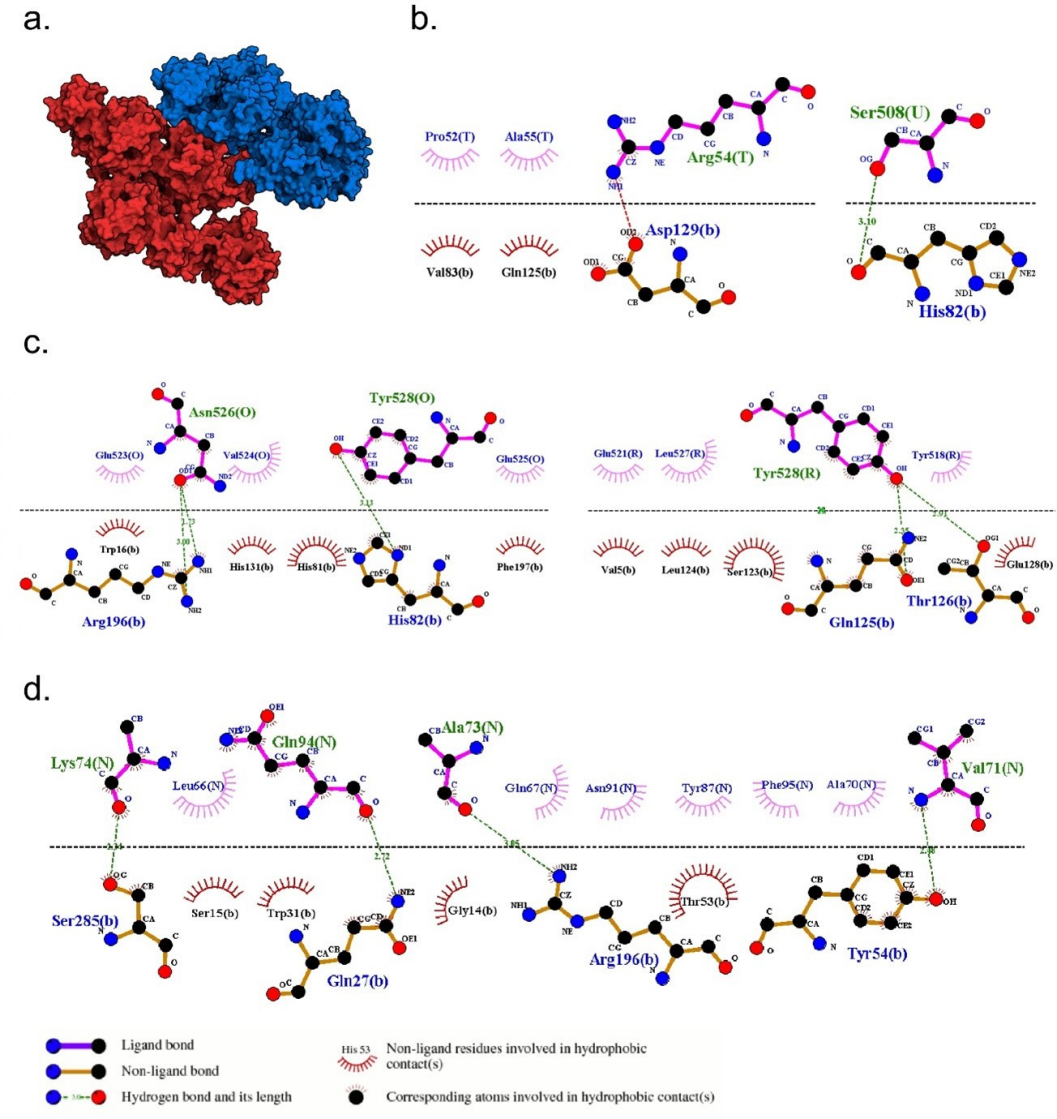
Protein docking interaction between 3BCZ and 4RIQ revealed by cLD (= 0.86) with a binding affinity of -341.21 kJ/mol. **a)** Structure of 3BCZ (red) and 4RIQ (blue) protein-protein complex. **b-d)** 2D representation of closest interacting residues around the protein-protein interaction interfaces, including multiple non-covalent bonds, for example, hydrogen bonds (green dotted line) and hydrophobic interactions (read and rose semi-circle with spikes). Residues for the 3BCZ are depicted in upper letters (T, U, O, R, N) and for the 4RIQ are depicted in lower letters.

**Fig 6:**
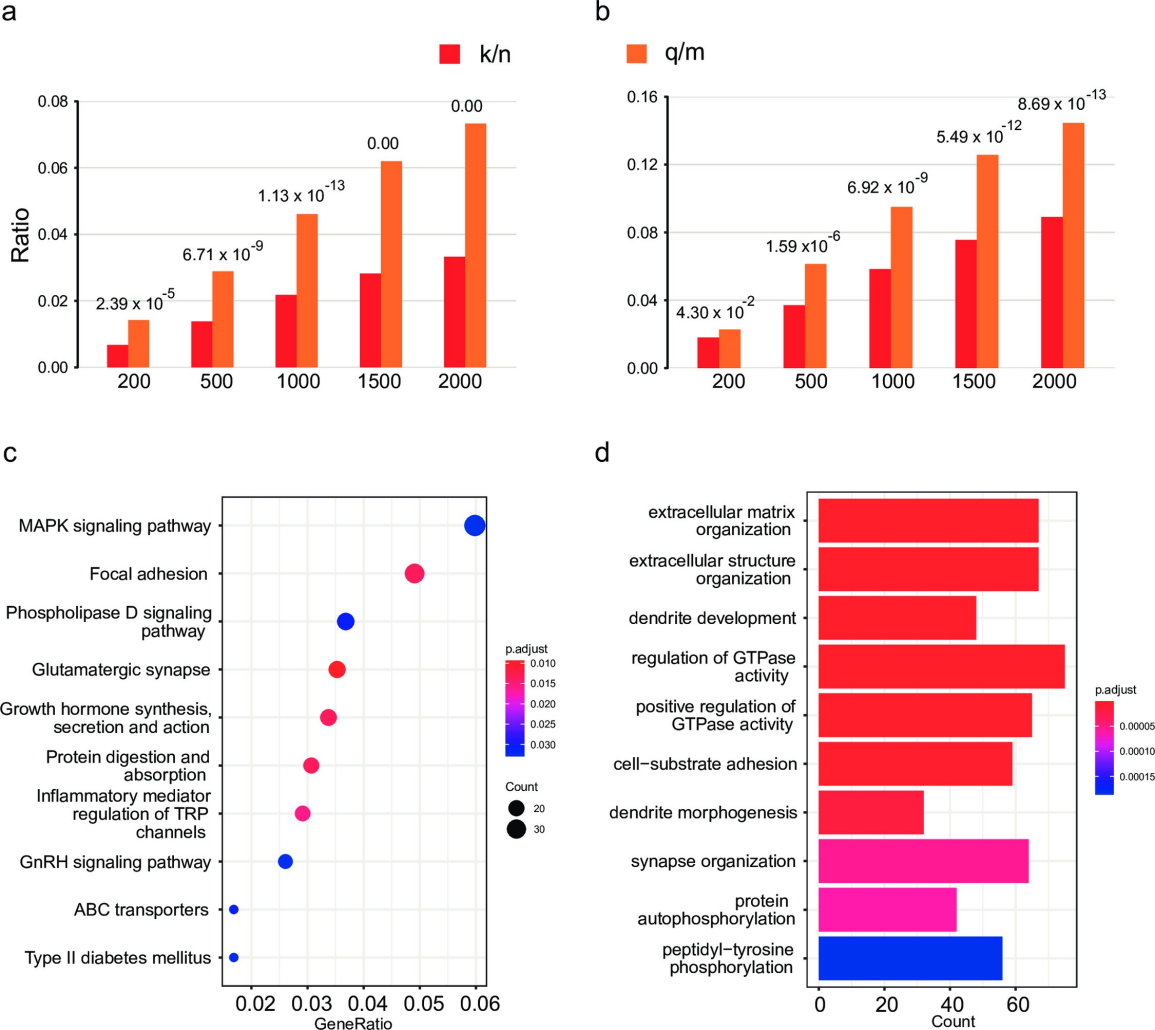
ΔcLD gene pairs in case/control association mapping data: annotation of top genes and enrichment of pathways. **a-b)** Group bar charts show the ratio between the number of selected genes being validated in the database dividing the number of genes in the database (q/m) as well as the number of selected genes dividing the total number of all known minus m (k/n). The values on the top of each bar are the p-values of the hypergeometric distribution probability test. The x-axis indicated the top gene pairs using different cutoffs, [200, 500, …, 2,000]. **a)** DisGeNET database. **b**) SFARI database. **c)** a dot plot showing the top 10 KEGG pathways ranked by the GeneRatio values. The size of the balls indicates the number of the genes enriched and the color indicates the level of the enrichment (P-adjusted values). The GeneRatio is calculated as count/setSize. ’count’ is the number of genes that belong to a given gene-set, while ’setSize’ is the total number of genes in the gene-set. **d).** a bar plot showing the top 10 enriched biological processes ranked by p-values. The correlation is more significant as the red/blue ratio increases. The number on the x-axis indicates the number of genes that belong to a given gene set.

We thoroughly tested the property of cLD in terms of it *r*^2^ version. First, using both theoretical closed-form derivation and bootstrapped simulations (**Verification and Comparison**), it is verified that cLD enjoys way lower variance than the standard LD when applied to rare variants, evidencing cLD’s higher stability (**Fig 2**). We then applied cLD to four scenarios in genetic analysis (**Applications**), discovering additional knowledge that have not been reported (or attempted but negatively reported) using standard LD (**Figs 3–6**).

## Description of the method

**The input data** to calculate cLD is a set of *haplotype sequences* representing a population of interest. To convert genotype data into inferred haplotypes, we recommend the use of ShapeIt2 [13] and MVNcall [14] that have been used to generate the Phase 3 haplotype data in the 1000 Genomes Project. The cLD is defined on two genetic *regions*. Users will specify a list of regions for which the cLD values between each pair of regions will be calculated. Only rare variants (defined by a user specified MAF maximal cutoff, e.g., 0.5%) are included in the calculation of cLD. The sizes of regions will influence the cLD calculations, although our practice shows that the default setting that specifies each gene as a region will lead to appropriate outcomes (see **Discussion** for more analysis).

### The intuitive idea of defining cLD

To explain the difference between LD and cLD, we recall the calculation of traditional LD between two genetic variants, with minor alleles *A* and *B*, respectively: The essential part is the calculation of individual MAFs *P*_*A*_ and *P*_*B*_ and the frequency that *A* and *B* show up in the same haplotype, *P*_*AB*_. Based on these, the difference *D* = *P*_*AB*_ ‒ *P*_*A*_*P*_*B*_ dividing a normalization defines LD. To calculate cLD between two regions, *A* and *B*, we first use cMAF to define *P*_*A*_ and *P*_*B*_ (the proportion of individual haplotypes carrying at least one rare variant within regions *A* and *B*, respectively); and then *P*_*AB*_, the proportion of haplotypes who have at least one rare variant in both regions *A* and *B* (**Fig 1B**). With the cLD version of *P*_*A*_ and *P*_*B*_ and *P*_*AB*_ ready, the same way for LD is used to calculate cLD. Mathematical details are spelt out in below and **Sections 1.1 and 1.2 in S1 Text**).

### Mathematical definition of LD and cLD

The calculation of standard LD between two bi-allelic genetic variants *A* and *B* is based on the MAFs *P*_*A*_ and *P*_*B*_, as well as *P*_*AB*_, the frequency of these two alleles of *A* and *B* showing up together. Then one calculates the unnormalized disequilibrium statistic *D* = *P*_*AB*_ ‒ *P*_*A*_*P*_*B*_. To rescale the statistic based on allele frequency, one can normalize *D* by dividing it by the allele frequency variances:

$$r^{2}=\frac{D^{2}}{P_{A}\left(1-P_{A}\right)P_{B}\left(1-P_{B}\right)}.$$

An alternative definition to *r*^2^ is *D*′, which has a different way of normalization. In this paper, we used *r*^2^ as the representative. Because LD involves *P*_*A*_ and *P*_*B*_ in the denominator, it is highly instable when *P*_*A*_ or *P*_*B*_ are close to zero, which means LD cannot be used if *A* or *B* are rare variants.

The cLD statistic is designed to handle the above problem by aggregating rare variants cumulatively. More specifically, here we look at two sets of variants in two genetic regions, e.g., two genes, again namely *A* and *B*. Assuming that there are *m* SNVs in gene *A*, and there are *r* SNVs in gene *B*. Also, we assume the sample size is *n*. Then, for gene *A*, we use *S*_1*i*_,*S*_2*i*_,…,*S*_*mi*_ to denote the allele of the *s*-th SNVs (s = 1,2,…,*m*) in the *i*-th individual (*i* = 1,2,…,*n*). Similarly, for gene *B*, we use {*K*_1*i*_,*K*_2*i*_,…,*K*_*ri*_} to denote the allele of the *k*-th SNV (*k* = 1,2,…,*r*) in the *i*-th individual (*i* = 1,2,…,*n*). Note that *S*_*si*_ and *K*_*ki*_ is either 0 or 1. (0 denotes a major allele, whereas 1 denotes a minor allele).

Then we have the cMAF (*P*_*A*_ & *P*_*B*_) defined below

$$P_{A}=\frac{1}{n}\sum_{i=1}^{n}I\left(\sum_{s=1}^{m}S_{si}\geq 1\right)$$

$$P_{B}=\frac{1}{n}\sum_{i=1}^{n}I\left(\sum_{k=1}^{r}K_{ki}\geq 1\right)$$

Where *I*(.) is the indicator function, yielding 1 if its logical argument evaluates to *true*, otherwise 0. *P*_*AB*_ is then defined as the proportion of individual haplotypes with a minor allele in both regions:

$$P_{AB}=\frac{1}{n}\sum_{i=1}^{n}I\left(I\left(\sum_{s=1}^{m}S_{si}\geq 1\right)+I\left(\sum_{k=1}^{r}K_{ki}\geq 1\right)=2\right)$$

Following the same formula of LD, however, using the above cumulative calculation of *P*_*A*_, *P*_*B*_, and *P*_*AB*_, we define the *r*^2^ version of cLD:

$$cLD=\frac{{\left(P_{AB}-P_{A}P_{B}\right)}^{2}}{P_{A}\left(1-P_{A}\right)P_{B}\left(1-P_{B}\right)}.$$

The more rigorous mathematical descriptions and the definition of *D*′ version is provided in **Sections 1.1 and 1.2 in S1 Text**

## Verification and comparison

### Rationale and outline of the verification

Both LD and cLD could be used to capture the correlation between two sets of rare variants. However, these two measures differ in the aspect of stability. Intuitively, as cMAF is always higher than MAF, cLD’s variance (reflecting its instability) should be lower than LD’s. We verify this intuition by deriving the closed-form of variance of both LD and cLD using multinomial distributions and their multivariate normal approximation as well as the multivariate Delta Method [15] (**Sections 2.1 and 2.2 in S1 Text**). Additionally, following the conventional statistical procedure of bootstrapping to empirically estimate stability, we sub-sampled half of each population sample 1,000 times to form bootstrapped distributions for both cLD and LD (**Section 2.4 in S1 Text**).

For both verifications, the 1000 Genomes Variant Call Data were used. In particular, the phased (i.e., haploid instead of diploid) variant call data of the Phase 3 of the 1000 Genomes dataset was obtained through The European Bioinformatics Institute’s dedicated FTP server [16].

### Derivation of theoretical variance of cLD in contrast to LD

To obtain the theoretical variance of cLD and LD, we derived their asymptotic distributions using the multivariate normal distribution and the multivariate Delta method [15]. The details are in **Sections 2.1 and 2.2 in S1 Text**.

### Assessing the instability of LD and cLD using bootstrapped distributions

To use bootstrapped samples to quantify instability, we randomly sampled half of the haplotypes in three main 1000 Genomes Project populations (EUR, AFR, or EAS) without replacement, and calculated the average cLD and average LD over the gene pairs within cMAF bins and repeated this procedure 1,000 times. Based on these bootstrapped cLD and LD values we formed bootstrapped distributions for cLD and LD respectively. The cLD and LD values have been divided by their mean in the corresponding populations under comparison to ensure that the standard deviation of data is understood in the context of its mean (**Section 2.3 in S1 Text**). Our comparisons are therefore a unit-less representation of this relationship and allows for comparison between diverse data sets. More specifically, we randomly sampled 1,000 genes and assessed their pairwise LD and cLD in stratified cMAF bins (**Section 2.4 in S1 Text**) using half of the haplotypes in the given population (AFR, EAS or EUR). These randomly drawn subsamples (each with half of the individuals in the original population) form bootstrapped samples. We define the LD of a gene pair as the average value of LD over all rare SNV pairs within that gene pair. In each iteration, we calculate the average cLD over the gene pairs in each bin (**Section 2.4 in S1 Text**).

### Observation: High stability of cLD in contrast to standard LD

By plugging in the allele frequencies calculated using the 1000 Genomes Project data [6] into the derived closed-form variances (**Section 2.3 in S1 Text**), we observed that the variance of cLD is at least six orders of magnitudes smaller (i.e., more stable) than the alternative—calculating LD directly on rare variants in all ethnic populations and all cMAF bins (**Fig 2A, Aa and Ba in S1 Text**). Additionally, the subsampling showed that cLD exhibits much slimmer bootstrapped distributions than LD across all cMAF bins and all three ethnic groups (**Fig 2B, Ab and Bb in S1 Text**), further confirming the greater stability of cLD compared to traditional measures of LD.

## Applications

### Application 1: cLD reveals linkage disequilibrium between 3D contact regions where standard LD fails

#### Rationale and outline

In this application, we show a distinct advantage of cLD over LD: the ability to reveal linkage disequilibrium between 3D contact regions. By aggregating information from multiple independent mutations, cLD is sensitive to subtle interactions poorly reflected by LD (which can only account for two at a time). As such, cLD captures more biological interactions in addition to traditional LD that focuses more on the lack of recombination. Interactions within the 3D structure of genomes is one place where this difference allows for insight from cLD where LD-based methods fail. The availability of high-throughput experimental technologies that can assess chromatin conformation such as Hi-C [17,18] allows researchers to analyze genetic regions that are in close contact in 3D spatial structure. There was a widely disseminated expectation that the 3D genomic interaction in the form of chromatin contact may leave a footprint in genetic LD [19]. Motivated by such expectation, Whalen and Pollard calculated standard LD based on common variants (MAF>0.05) in 1000 Genomes Project data [6] and reported negative results stating that genetic LD map is not overlapping with the 3D contact map [19].

#### Procedures of Calculating cLD and LD for gene pairs in 3D interaction regions

To revisit the previously negatively reported relationship between 3D interaction regions and genetic linkage disequilibrium [19], we calculated both cLD and LD in a Hi-C assessment in the developing brain [20], which has 27,982 brain-specific paired 3D-interacting regions, measured from neurons derived from human induced pluripotent stem cells (hiPSCs).

Again, the 1000 Genomes Project data were used. We first calculated the physical distance between the genes in each pair and separate the gene pairs into 13 distance groups, from 35Kb, 70Kb, 140Kb, …, to 143.2Mb, forming a geometric progression with a ratio of 2 (**Section 3.1 in S1 Text**). After stratifying all gene pairs into distance groups, within each distance group, we calculated cLD between all gene pairs and further split them into two categories: the ones that are located in 3D interaction regions (assessed by Hi-C experiments) and the ones that are located in non-3D interaction regions. The gene pairs with only one gene in an interaction region were discarded. Finally, the average cLD values over gene pairs within interaction and non-interaction regions were used to conduct the comparison, quantified by two two-sample tests, namely Mantel-Haenszel and Fisher exact tests (**Section 3.4 in S1 Text**).

As Whalen & Pollard’s work [19] is not at the resolution of genes, we re-calculated standard LD using common variants based on gene pairs (**Section 3.2 in S1 Text**). Briefly, the procedure of calculating standard LD mirrors the one used above for cLD using the same distance groups and 3D-interaction vs non-interaction categories. As standard LD is defined by individual variants (not by genes), the following averaging steps were taken. For each gene pair in the 3D interaction regions, we randomly chose 2,000 rare variant pairs from it to calculate their LD values. For each selected rare variant pair, we calculated its distance and then, among the gene pairs without 3D interactions, we randomly selected another rare variant pair with the same or closest possible distance (**Section 3.2 in S1 Text**). As a result, we achieved 2,000 randomly selected variant pairs from gene pairs without interaction that were matched up with the 2,000 variant pairs from gene pairs with interaction. The average values of the 2,000 variant-pairs were deemed as the LD between the gene pair.

#### Observations

By reanalyzing the 1000 Genomes sequencing data and Hi-C data [17,18] in the developing brain using cLD on rare variants (**Materials and Methods; Sections 3.1 and 3.2 in S1 Text**), we revealed that the 3D chromatin interactions did leave genetic footprints in the form of higher cLD in pairs of genes that are in the 3D contact regions (**Fig 3A and 3D in S1 Text**). To assess the statistical significance of the enrichment of cLD in 3D contact regions, we conducted Mantel-Haenszel and Fisher exact tests (**Section 3.4 in S1 Text**), both of which are highly significant (P-value < 1.0E-50; **Tables J and R and Section 3.4.1 in S1 Text**).

Our re-calculation of standard LD using common variants based on gene pairs (**Section 3.2 in S1 Text**) shows a subtle effect (**Fig 3B and 3E in S1 Text**) but still not statistically significant with Mantel-Haenszel and Fisher exact tests (P-value = 0.999**; Tables K and P and Section 3.4.1 in S1 Text**).

Additionally, we checked the ratio between the number of pairs of genes within the 3D contact regions and the number of pairs outside the 3D contact regions as a function of their cLD cut-off. More specifically, we prespecified a cLD value cutoff and only counted the gene pairs with cLD value higher than this cutoff; then we separated the number of genes within or outside 3D contact regions and calculated their ratios (**Section 3.5 in S1 Text**). Indeed, we found that the ratios are significantly larger than 1.0 and increase as the cLD cutoffs increase (**Fig 3C–3E and Table S in S1 Text)**.

Taking together, 3D interactions clearly overlap with genetic interactions; and cLD is instrumental in observing this whereas standard LD fails.

### Application 2: cLD is enriched in known interacting genes

To demonstrate that gene-gene interactions leave footprints in rare genetic mutations regardless of their physical positions, we computed the distribution of average cLD among interacting pairs genes reported in four frequently used interaction databases, namely Reactome [21], BioGRID [22], MINT [23] and IntAct [24] (**Materials and Methods; Section 3.3 in S1 Text**). The related datasets were downloaded from their corresponding websites and the IDs were matched using standard gene models (gencode v17). To quantify the distance between genes, only gene pairs within the same chromosomes were used. Calculation of cLD and LD follows the same procedure as described for the 3D-interaction analysis, and the same two-sample tests (Mantel-Haenszel and Fisher exact tests) were used to quantify the significant levels (**Section 3.4 in S1 Text**). We compared this distribution of cLD against a null distribution formed by all pairs of genes. Indeed, the comparisons led to the expected result: for gene pairs separated by any physical distance within 2MB, cLD is elevated in interacting gene pairs (**Fig 4 and F in S1 Text**). Again, the Mantel-Haenszel and Fisher exact tests confirm that the differences are significant (P-value < 1.0E-20; **Table Q and Section 3.4.2 in S1 Text**).

### Application 3: cLD identified novel pairs of likely interacting proteins

To examine the novel gene pairs with higher cLD values have the receptor-ligand interactions of their translated proteins, we performed protein-docking analysis to obtain the evidence. HDOCKlite-v1.1 [25,26] was employed for conducting the protein-protein docking analysis between the cLD prioritized protein pairs (**Section 4 in S1 Text**). The protein’s crystal structure was obtained from the Protein Data Bank [27] and further validated [28] (**Section 4.1 in S1 Text**). The output file of the docked complex was visualized by PyMOL 2.5.1 [29], and the 2D plot of the protein-protein binding region was analyzed and deduced using LigPlot+ v.2.2 [30] (**Section 4.2 in S1 Text**).

Looking at all pairs of genes, we observed several pairs without prior evidence of interaction with extraordinarily high cLD, such as between genes *MEMO1* and *DPY30* (encoding proteins 3BCZ and 4RIQ, respectively) with a cLD of 0.86. We then conducted systematic protein docking analysis for the genes of large cLD values (top 0.01% among all gene pairs) with cMAF > 0.05 and existing IDs in PDB, however, not reported in any interaction databases (**Materials and Methods; Section 4.1 and Table L in S1 Text**). These criteria lead to 19 pairs of genes for protein-docking. We found multiple lines of evidence of the interaction at protein level for five pairs (**Table M in S1 Text**) in terms of both binding affinity and interacting residues (**Fig 5A–5D and G–J in S1 Text**).

### Application 4: Differences in cLD distinguish cases/controls in Autism exome data

#### Rationale and procedures

To explore the use of cLD in distinguishing cases and controls in a typical association study, we calculated cLD using the whole exome sequencing data to study Autism Spectrum Disorder (ASD) [31] [dbGaP ID: phs000298.v4.p3] (**Section 5.1 in S1 Text**). We first calculated cLD values for each gene pair for cases and controls groups separately. Then, we calculated the absolute differences between the cLD values in case and control groups for each gene pair, which was called ***ΔcLD***. These absolute differences were sorted from largest to smallest. The top ranked genes pairs were collected and called cLD-differential gene pairs, or ΔcLD genes (**Section 5.2 and 5.3 in S1 Text**). Based on their ΔcLD values, we selected the top 200, 500, 1,000, 1,500 and 2,000 cLD-differential gene pairs (i.e., ΔcLD genes) and used the genes sets for the downstream functional annotations. We utilized two different databases, Simons Foundation Autism Research Initiative (SFARI) [32] and DisGeNET [33] as the gold-standard because they are frequently used in the field of ASD studies and general disease gene queries, respectively. We used the hypergeometric distribution probability to assess the p-value of the significance of enrichment of the cLD-differential genes against the background of gold-standard genes (**Section 5.4 in S1 Text**). Additionally, using the top 2,000 cLD-differential gene pairs, we conducted GO enrichment [34] and KEGG pathway analysis [35].

#### Observations

The genes included in the pairs with high ΔcLD scores are highly enriched in both the Autism related genes in DisGeNET (**Fig 6A**) and SFARI (**Fig 6B**). Gene Ontology [34] and pathways (KEGG)[35,36] enrichment analysis for the high ΔcLD genes (**Materials and Methods; Table T and Section 5.4 in S1 Text**) also showed sensible biological functions and pathways (**Fig 6C and 6D**) that are well supported by the literature (**Section 5.4 in S1 Text**) [34–49]. By taking a closer look of the 20 genes identified by the top 10 gene pairs with the highest ΔcLD values, found that 14 genes (70%) have been reported to be associated with ASD, including *DENND4A*, *EFCAB5*, *ABI2*, *RAPH1*, *MSTO1*, *DAP3*, *ARL13B*, *PRB2*, *PRB1*, *ZNF276*, *FANCA*, *ADAM7*, *SLC26A1* and *TUBB8* (**Table N in S1 Text**). Moreover, among the rest of six genes, we also identified indirect links of two, *RAB11A* and *IDUA* with ASD (**Section 5.3 in S1 Text**).

## Discussion

LD is a critical concept applicable to many types of genetic analyses. In this work, we have defined cLD, a new statistic addressing the association between genetic regions using rare genetic variants. By both closed-form derivations and statistical simulations, we proved the stability of cLD in contrast to the high instability of standard LD (when applied to rare variants). The stability and the focus on biological interaction allows cLD to capture additional information from the distributions of many variants segregating in a population at low frequencies within particular regions of a genome. Indeed, by applying cLD to real data, we observed interesting overlapping pattern of 3D interactions and genetic interactions that have been negatively reported by using standard LD. We also successfully analyzed protein docking and association mapping, providing two broadly impactable use-cases of cLD. With its demonstrated power in identifying gene and protein interactions, cLD might offer an essential tool to analyze biological interactions and their evolution using rare genetic variants.

The cLD statistics is inspired by Burden Tests pioneered in the field of association mapping, which have been invented in 2008[50]; and extensively researched over the past decade[51]. Burden tests have been used to identify the association between genes and complex traits via the aggregation of rare variants [52,53]. The definition of cLD is an extension of cMAF, the core concept pioneered by burden tests, to second order interactions. In association mapping, many methods utilize kernel machine to measure the similarity between individuals at the focal region, e.g., SKAT [54], which are also popularly used. It should be feasible to apply SKAT-type techniques to define cLD, for instance, by using combined kernel calculations [55]. We leave the detailed definition and theoretical/practical investigations to the future work.

In the present work, only rare-rare cLD are considered. However, in a similar statistical framework, rare-common cLD may be defined as well. Given a common variant A, and a region B where rare variants are to be aggregated, one may first calculate P(A) as MAF and P(B) using cMAF; then P(AB) could be defined by the change that there is at least one rare variant in B showing up in the same haplotype with A. As such, cLD between A and B can be calculated.

Many statistical and computational strategies have been developed to identify gene-based Gene-Gene Interactions (GGIs). These include canonical correlation-based U statistic (CCU)[56], Kernelized CCU, or KCCU [57,58], non-parametric entropy-based technique called GBIGM [59,60], as well as pattern mining methods[61]. Although cLD has identified many gene-pairs Application 4, there is no rigorous statistical test to quantify the false positive and the P-value of the significance. Assuming there is no correlation between cases and controls, we derived the asymptotic distribution of ΔcLD (**Section 2.3.2 in S1 Text**) which will lay the ground of developing an additional tool to detect gene-gene interactions in case/control GWAS data. This will be an interesting future work for us to follow.

**Fig 7:**
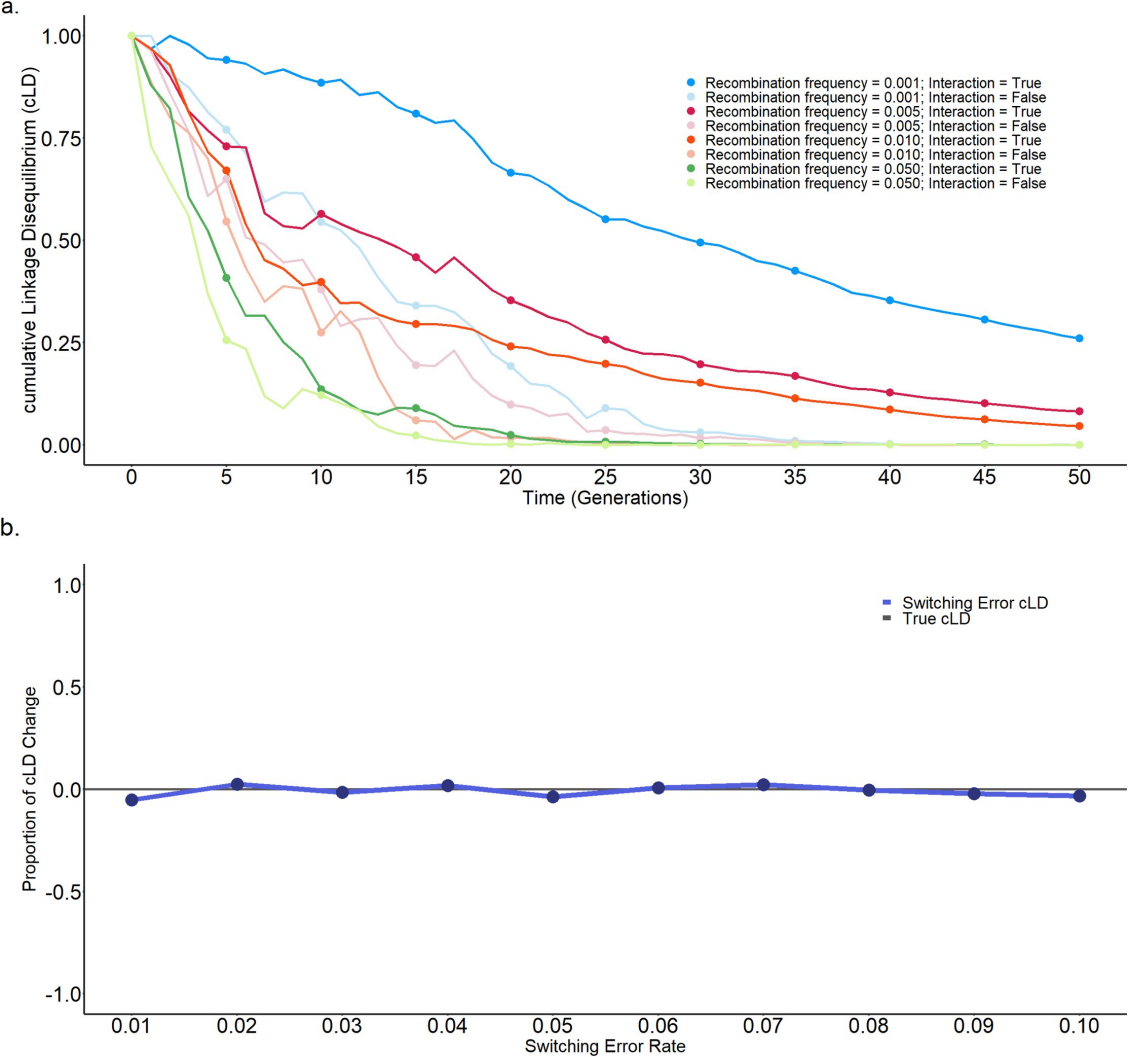
The alternation of cLD under multiple conditions. **a)** Recombination and interaction. Using simulations, we demonstrate the temporal decay of cLD. The pace of cLD decay with respect to recombination rate is illustrated. Notably, the rate of cLD decay is decelerated in the presence of genetic interactions. **b)** Switching error in phasing. Comparison of cLD in the presence of switching error to true cLD across multiple gene pairs are illustrated. We computed the average cLD value under various switching error rate settings (0.01, 0.02, …, 0.1). For each switching error rate, we used the proportion of cLD change to quantify its deviation from true cLD. As illustrated in the figure, in the presence of switching error, cLD remains remarkably close to true cLD, even as the switching error rate increases. This highlights the robustness of cLD against switching errors.

cLD is designed to capture biological interactions, therefore the cLD decay proportional to recombination may be slower in the presence of interactions. To thoroughly understand the behaviour of cLD under different conditions, we developed a new simulator and carried out an additional simulation. This software simulates a growing population and negative selection against individual mutations (two main reasons why rare variants are enriched in human genomics) but favour positive synergy between two mutations (**Section 6.2 in S1 Text**) to characterize the cLD decay with respect to number of generations with or without interactions. Analog to the standard reports of LD decay against generations, we reported the cLD decay against generations with or without interactions between two genes (**Fig 7A**). One can see that the cLD also decays along time, but it is slower when interactions are present. This confirms our intention and results in real data that cLD captures interactions through the combination of rare variants.

Although cLD is more stable than LD, the calculations may be confounded by some technical issues: (1) selection of genetic regions, (2) depth of sequencing, and (3) tools of phasing haplotypes. First, when carrying out a burden test, a critical consideration is the genetic region to apply the aggregation. Too large a region will cause the cMAF being one (i.e., too many rare variants), however too small a region will cause the cMAF close to zero (i.e., too few rare variants). Both will lead to meaningless outcome. The same trade-off applies to cLD, as cMAF plays a critical role in it. Based on our analysis, we suggest that a practical use is usually set a gene as the region. Additionally, the functional annotation-based selections used in burden tests [62] can also help prioritize rare variants to be selected to contribute to cLD.

Second, sequencing depth may also alter the values of cLD substantially because deeper sequencing will reveal more rare-SNVs. The main analysis in this paper is based on the Phase 3 data of the 1000 Genomes Project, which has a low coverage of 4X per individual. To investigate the difference between the low-depth and the newly released high-depth data[63], we calculated the distribution of number of rare variants (defined as MAF < 0.5%) per gene, cMAF per gene and cLD patterns. We indeed found substantial difference in number of variants per gene, which increased from a median of 86 (in low-depth data) to a median of 94 (in high-depth data). However, the overall distributions, expressed by a probability density functions (PDFs), of both number of rare variants per gene and cMAF generated by the low-depth and high-depth data remain quite similar (**Figs K and L in S1 Text**). Consistent to this observation, the observed the same pattern of cLD also remains the same (**Fig M in S1 Text**). This shows that cLD is generally robust to the number of rare variants as long as total numbers are in a reasonable range (which may be ensured by the selection of regions being a gene).

Third, phasing variants is a precondition of applying cLD, however phasing might be difficult for rare variants (in contrast to common variants) [64]. Our theoretical and bootstrap assessment both assume haplotypes are correctly obtained. In connection to this, we simulated haplotype data by adding switching errors reported by [64] and calculated cLD accordingly (**Section 6.3 in S1 Text**). The outcome based on the simulations shows that the change of cLD is at a reasonable scale (**Fig 7B**). Nevertheless, the practice in this work showing four successful applications suggests that the default phasing carried out by the 1000 Genomes Consortium worked well. In the applications, researchers using cLD should ensure phasing as accurate as possible. Efforts of phasing haplotypes focusing on rare variants may be also utilized in the future [65–67].

## Supporting information

S1 Text Fig A in S1 Tex. Stability of cLD and LD revealed by closed-form variance calculation and bootstrapped resampling. The EUR population is shown. a) The gene pairs were split into four different bins based on the cMAF values, i.e., < 0.05, 0.05–0.1, 0.1–0.2, and 0.2–0.4, which has been shown in the y-axis. The x-axis is the ratio between the variance of LD and cLD. b) Probability density distribution of cLD and LD by generating bootstrapped samples. **Fig B in S1 Text**. Stability of cLD and LD revealed by closed-form variance calculation and bootstrapped resampling. The AFR population is shown. a) The gene pairs were split into four different bins based on the cMAF values, i.e., < 0.05, 0.05–0.1, 0.1–0.2, and 0.2–0.4, which has been shown in the y-axis. The x-axis is the ratio between the variance of LD and cLD. b) Probability density distribution of cLD and LD by generating bootstrapped samples. **Fig C in S1 Text**. Stability of cLD and LD revealed by closed-form variance calculation and bootstrapped resampling. The EAS population is shown. a) The gene pairs were split into four different bins based on the cMAF values, i.e., < 0.05, 0.05–0.1, 0.1–0.2, and 0.2–0.4, which has been shown in the y-axis. The x-axis is the ratio between the variance of LD and cLD. b) Probability density distribution of cLD and LD by generating bootstrapped samples. **Fig D in S1 Text**. The comparisons of cLD values between the 3D chromatin interaction regions and non-interaction regions among 13 different distance groups in (a) the whole population, (b) AFR, and (c) EAS. The European population, EUR, has been displayed in the main text Fig 3A. **Fig E in S1 Text**. The comparisons of LD values between the 3D chromatin interaction regions and non-interaction regions among 13 different distance groups in (a) the whole population, (b) AFR, and (c) EAS. The European population, EUR, has been displayed in the main text Fig 3B. **Fig F in S1 Text**. The comparisons of cLD values between the gene-gene interaction regions and regions without interactions among 13 different distance groups in (a) the whole population, (b) AFR, and (c) EAS. The European population, EUR, has been displayed in the main text Fig 4. **Fig G in S1 Text**. Protein docking interaction between 1FYV and 4OM revealed by cLD (0.69) with a binding affinity of -266.77 kJ/mol. a) Structure of 1FYV (red) and 4OM (blue) protein-protein complex. b-c) 2D representation of closest interacting residues around the protein-protein interaction interfaces, including hydrogen bonds (green dotted line) and hydrophobic interactions (read and rose semi-circle with spikes). Residues for the 1FYV are depicted by uppercase letters (A, B) and for the 4OM are depicted by lower lowercase letter (a). **Fig H in S1 Text**. Protein docking interaction between 3S5N and 4HND revealed by cLD (0.52) with a binding affinity of -302.64 kJ/mol. a) Structure of 3S5N (red) and 4HND (blue) protein-protein complex. b-c) 2D representation of closest interacting residues around the protein-protein interaction interfaces, including hydrogen bonds (green dotted line) and hydrophobic interactions (read and rose semi-circle with spikes). Residues for the 3S5N are depicted by uppercase letter (A) and for the 4HND are depicted by lowercase letters (a, b). **Fig I in S1 Text**. Protein docking interaction between 1WVA and 6HO2 revealed by cLD (0.40) with a binding affinity of -263.19 kJ/mol. a) Structure of 1WVA (red) and 6HO2 (blue) protein-protein complex. b-c) 2D representation of closest interacting residues around the protein-protein interaction interfaces, including hydrogen bonds (green dotted line) and hydrophobic interactions (read and rose semi-circle with spikes). Residues for the 1WVA are depicted by uppercase letter (I) and for the 6HO2 are depicted in lowercase letter (a). **Fig J in S1 Text**. Protein docking interaction between 3L81 and 5FUR revealed by cLD (0.33) with a binding affinity of -277.36 kJ/mol. a) Structure of 3L81 (red) and 5FUR (blue) protein-protein complex. b-c) 2D representation of closest interacting residues around the protein-protein interaction interfaces, including hydrogen bonds (green dotted line) and hydrophobic interactions (read and rose semi-circle with spikes). Residues for the 3L81 are depicted by uppercase letters (A, B) and for the 5FUR are depicted in the lowercase letter (a). **Fig K in S1 Text**. Probability Density Functions of Distributions of the Number of Rare SNVs per Gene in Low-Depth (Red) and High-Depth (Blue) Data. The median number of rare SNVs per gene in the low-depth data is 86, while in the high-depth data it is 94. Although there is a difference in the median values, the distribution of the number of rare SNVs per gene is not significantly different between the two datasets. The definition of rare SNV is MAF < 0.005. **Fig L in S1 Text**. Probability Density Functions of Distributions of cMAF in Low-Depth (Red) and High-Depth (Blue) Data three population groups. (a) EUR population; (b) AFR population; (c) EAS population. The red line represents the low-depth data (4X coverage) and the blue line represents the high-depth data (30X coverage). The distributions of cMAF are very similar in the three populations, indicating that cLD performs well similarly in low-depth and high-depth data. The cMAF calculation uses a standard cutoff of defining the rare variants as MAF < 0.005. **Fig M in S1 Text**. The comparisons of cLD values between the 3D chromatin interaction regions and non-interaction regions among 13 different distance groups using High-depth data. (a) EUR, (b) AFR, and (c) EAS. **Fig N in S1 Text**. Illustration of the robustness of cLD, a) original SNVs b) simulated switching error. a) In the original data, we have 7 individuals and 7 SNVs in the gene. The gene line at the bottom is computed through integration across SNVs. b) We simulated phasing errors in individuals 1, 2, and 3 at SNVs 6–7, 1–3, and 7, respectively. However, after integration, the gene line remains unchanged. This example illustrates the robustness of the cLD against switching errors. **Table A in S1 Text.** Example of MAF for single SNV. **Table B in S1 Text.** Cumulative effects of SNVs in a region. **Table C in S1 Text.** Cumulative genetic “allele”s for cLD calculation. **Table D in S1 Text.** Example of SNVs data for cLD calculation. **Table E in S1 Text.** Example of cumulated gene alleles for cLD calculation. **Table F in S1 Text**. Data of two SNVs. **Table G in S1 Text**. Multinomial modelling of two SNVs. **Table H in S1 Text**. Multinomial modelling of two genes. **Table I in S1 Text**. The quantiles of cLD values in the 3D chromatin interaction regions and non-interaction regions among 13 different distance groups in three populations. **Table J in S1 Text**. EUR counts for Mantel-Haenszel test (3D-interaction, cLD). **Table K in S1 Text**. EUR counts for Mantel-Haenszel test (Hi-C, LD). **Table P in S1 Text**. Average cLD and LD differences under different distance groups within Hi-C regions. **Table Q in S1 Text**. The number of gene pairs above and below the cutoff (the 0.5 quantile), the ratio and the statistic tests between interaction and no interaction groups. **Table R in S1 Text**. The number of gene pairs above and below the cutoff (the 0.5 quantile), the ratio and the statistic tests within and without Hi-C regions. **Table S in S1 Text**. Comparisons between the number of gene pairs with cLD values larger than the cutoffs in whole genome and within 3D regions (only on chromosome 2). **Table L in S1 Text**. List of 19 gene pairs (not reported in any databases) with large cLD values with cMAF > 0.05 and existing IDs in PDB. **Table M in S1 Text**. List of candidate proteins with their respective cLD values and binding affinities. All candidates formed stable protein-protein complexes with negative binding energies. **Table N in S1 Text**. From the top 10 gene pairs with the highest cLD values, we identified 20 unique genes. 14 out of these 20 genes (70%) have been reported to be associated with ASD, including DENND4A, EFCAB5, ABI2, RAPH1, MSTO1, DAP3, ARL13B, PRB2, PRB1, ZNF276, FANCA, ADAM7, SLC26A1 and TUBB8. **Table T in S1 Text**. The success, success rate and p-value of hypergeometric distribution probability for DisGeNET and SFARI database from top 200 to 2000 gene pairs. **Table O in S1 Text**. List of parameters and their descriptions explaining their functionality.
(PDF)
