# Supplementary material for: cLD: Rare-variant linkage disequilibrium between genomic regions identifies novel genomic interactions
Source: PLoS Genet. 2023 Dec 18;19(12):e1011074. doi: 10.1371/journal.pgen.1011074 (PMC10758262; doi:10.1371/journal.pgen.1011074)
Supplement: S1 Text — Stability of cLD and LD revealed by closed-form variance calculation and bootstrapped resampling. The EUR population is shown. a) The gene pairs were split into four different bins based on the cMAF values, i.e., < 0.05, 0.05–0.1, 0.1–0.2, and 0.2–0.4, which has been shown in the y-axis. The x-axis is the ratio between the variance of LD and cLD. b) Probability density distribution of cLD and LD by generating bootstrapped samples. Fig B in S1 Text. Stability of cLD and LD revealed by closed-form variance calculation and bootstrapped resampling. The AFR population is shown. a) The gene pairs were split into four different bins based on the cMAF values, i.e., < 0.05, 0.05–0.1, 0.1–0.2, and 0.2–0.4, which has been shown in the y-axis. The x-axis is the ratio between the variance of LD and cLD. b) Probability density distribution of cLD and LD by generating bootstrapped samples. Fig C in S1 Text. Stability of cLD and LD revealed by closed-form variance calculation and bootstrapped resampling. The EAS population is shown. a) The gene pairs were split into four different bins based on the cMAF values, i.e., < 0.05, 0.05–0.1, 0.1–0.2, and 0.2–0.4, which has been shown in the y-axis. The x-axis is the ratio between the variance of LD and cLD. b) Probability density distribution of cLD and LD by generating bootstrapped samples. Fig D in S1 Text. The comparisons of cLD values between the 3D chromatin interaction regions and non-interaction regions among 13 different distance groups in (a) the whole population, (b) AFR, and (c) EAS. The European population, EUR, has been displayed in the main text Fig 3A. Fig E in S1 Text. The comparisons of LD values between the 3D chromatin interaction regions and non-interaction regions among 13 different distance groups in (a) the whole population, (b) AFR, and (c) EAS. The European population, EUR, has been displayed in the main text Fig 3B. Fig F in S1 Text. The comparisons of cLD values between the gene-gene interaction regions and regi [file pgen.1011074.s001.pdf]

cLD: Rare-variant linkage disequilibrium between genomic regions identifies novel genomic interactions

S1 TEXT

Dinghao Wang, Deshan Perera, Jingni He, Chen Cao, Pathum Kossinna,

Qing Li, William Zhang, Xingyi Guo, Alexander Platt, Jingjing Wu, Qingrun Zhang

Table of Contents

Table of Contents 1

1 Definitions and data source 3

1.1 Definition of LD and cLD 3

1.2 An example 7

1.3 Dataset and the population labels 8

2 Asymptotic Properties of cLD 10

2.1 Derivation of closed-form distribution of LD 11

2.1.1 Multinomial distribution 11

2.1.2 Asymptotic normality approximation of LD 13

2.2 Derivation of closed-form distribution of cLD 15

2.3 Comparison of variances using closed-form distributions 17

2.3.1 Comparison of variances between cLD and LD 17

2.3.2 Asymptotic distribution of cLD changes in Case and Control groups . 19

2.4 Estimating stability by Bootstrapped distributions 20

3 Applying cLD to real sequence data 26

3.1 Gene pairs grouped by distance bins 27

3.2 LD and cLD in 3D interacting regions assessed by Hi-C experiments 27

3.3 LD and cLD in databases for gene-gene interactions 30

3.4 Two-sample tests assessing significance levels 33

3.4.1 Two-sample Tests of cLD & LD in 3D interacting regions 35

3.4.2 Two-sample tests of cLD in gene-gene interaction databases 36

3.5 Enrichment of high-cLD pairs in 3D Interaction regions 37

4 Protein interactions and docking 40

4.1 Pipeline for protein-protein docking analysis 40

4.2 Protein-protein docking results 41

5 Association mapping using cLD and annotations 47

5.1 Data source and quality control 47

5.2 Calculating cLD in case-control datasets 48

5.3 Enrichment of cLD-differential genes in functional databases 48

5.4 Enrichment of cLD-differential genes in GO and KEGG 51

6 Evaluating cLD under different conditions 53

6.1 Sequencing depth 53

6.2 Recombination and interaction 57

6.3 Switching error in haplotype inference 59

Chapter 1

Definitions and data source

In this chapter, we mathematically defined the new statistic cLD, essentially an extension of LD analogue to the extension from MAF to cMAF. We presented its detailed mathematical formulation as well as an example. To facilitate the proofs in the next Chapter regarding the asymptotic properties of cLD (in contrast to LD), we used a mathematical language that describes the alleles and haplotypes as random variables. For this reason, LD will also be formulated using similar language and notations. We also presented details of the 1000 Genomes Project data based on which we calculated cLD and LD in various populations.

1.1 Definition of LD and cLD

For a specific genomic location, we used 1 and 0 to denote the presence and absence of a non-reference allele, respectively. Then, the occurrence of a variant in a haplotype naturally follows a Bernoulli distribution, in which the success probability could be estimated by the Minor Allele Frequency (MAF) at this locus. In the example given in Supplementary Table A, there are 5 individual single-SNV haplotypes $X_1, \ldots, X_5$ and each of them takes value 0 or 1.

Without losing generality, consistent to the practise in most literature, we assumed that $X_1, \ldots, X_5$ are independent and identically distributed (i.i.d.) samples drawn from

Table A: Example of MAF for single SNV

POS	Gene	Info	HG1-1	HG1-2	HG2-1	HG2-2	HG3-1	MAF
SNV1	1	...	X_1	X_2	X_3	X_4	X_5	p

a Bernoulli distribution with success rate that is approximated by the MAF of this mutated allele.

Next we defined the scenario of cumulative case, i.e., at the region level (such as a gene), based on the genotype displayed in Supplementary Table B. In this table, the shaded rows represent the genomic region for Gene 1 and the un-shaded rows are for Gene 2. X_{ijk} denote whether a minor allele is present (1) or absent (0) in the i-th haplotype of the j-th gene and k-th variant in this gene, $i = 1, \ldots, n$ (number of haplotypes, which is twice of the number of individuals), $j = 1, \ldots, g$ (number of genes), $k = 1, \ldots, v$ (number of variants in this gene). Since X_{ijk}'s only take value 0 or 1, we again assume they follow Bernoulli distribution, i.e. $X_{ijk} \sim B(1, p_{jk})$, where p_{jk} is the MAF of the and k-th variant in the j-th gene. $B(n, p)$ denotes the Binomial distribution with n trials and p being the chance of success for each trial. Note that we only considered rare variants with MAF$\leq$ 0.005. The true MAF value p_{jk} could be estimated by the sample MAF $\hat{p}_{jk}$. Also, we assumed that X_{ijk}, $i = 1, \ldots, n$, are independent random variables.

Table B: Cumulative effects of SNVs in a region

POS	Gene	Info	HG1-1	HG1-2	HG2-1	HG2-2	HG3-1	other haplotypes	MAF
6034	1	...	X_{111}	X_{211}	X_{311}	X_{411}	X_{511}	...	p_{11}
6041	1	...	X_{112}	X_{212}	X_{312}	X_{412}	X_{512}	...	p_{12}
6047	1	...	X_{113}	X_{213}	X_{313}	X_{413}	X_{513}	...	p_{13}
6057	1	...	X_{114}	X_{214}	X_{314}	X_{414}	X_{514}	...	p_{14}
6064	1	...	X_{115}	X_{215}	X_{315}	X_{415}	X_{515}	...	p_{15}
...	...	...	...	...	...	...	...	...	...
7064	2	...	X_{121}	X_{221}	X_{321}	X_{421}	X_{521}	...	p_{21}
7124	2	...	X_{122}	X_{222}	X_{322}	X_{422}	X_{522}	...	p_{22}
7235	2	...	X_{123}	X_{223}	X_{323}	X_{423}	X_{523}	...	p_{23}
...	...	...	...	...	...	...	...	...	...

In order to define the cLD, with the data of rare variants in Supplementary Table B, we first formed the cumulative ”allele”s for each gene, illustrated in Supplementary Table C. In this table, $X_{i1} = I(\sum_k X_{i1k} > 0)$ and $X_{i2} = I(\sum_k X_{i2k} > 0)$, where $I(.)$ is the logical function that will yield to 1 if the logical expression is true, and 0 otherwise. Intuitively, any SNVs being 1 will lead to the cumulative allele to be 1. The cumulative allele will be 0 only if all SNVs are 0. By counting the number of 1 in each row and dividing the total number of columns (i.e., haplotypes), one can straightforwardly calculate the cumulative MAF, or cMAF. Formally:

$$cMAF = \frac{\sum_i X_{i1}}{n}. \tag{1.1}$$

Table C: Cumulative genetic “allele”s for cLD calculation

POS	Gene	Info	HG1-1	HG1-2	HG2-1	HG2-2	HG3-1	other haplotypes
Gene 1	1	...	X_{11}	X_{21}	X_{31}	X_{41}	X_{51}	...
Gene 2	2	...	X_{12}	X_{22}	X_{32}	X_{42}	X_{52}	...

Analogue to the traditional definition of the r^2 form of LD between to loci being

$$r^2 = \frac{(p_{AB} - p_A p_B)^2}{p_A(1-p_A)p_B(1-p_B)}. \tag{1.2}$$

where A and B are two loci, $p_{(.)}$ denotes the MAF, and the cumulative version of p_A, p_B and p_{AB} being

$$p_A = \frac{\sum_i X_{i1}}{n}, \tag{1.3}$$

$$p_B = \frac{\sum_i X_{i2}}{n}, \tag{1.4}$$

$$p_{AB} = \frac{1}{n}\sum_i X_{i1}X_{i2}. \tag{1.5}$$

we defined the cLD between Gene 1 and Gene 2 as:

$$cLD = \frac{\left[\frac{1}{n}\sum_i X_{i1}X_{i2} - \frac{1}{n^2}(\sum_i X_{i1})(\sum_i X_{i2})\right]^2}{\frac{\sum_i X_{i1}}{n}(1-\frac{\sum_i X_{i1}}{n})\frac{\sum_i X_{i2}}{n}(1-\frac{\sum_i X_{i2}}{n})}. \tag{1.6}$$

The above definition estimates the correlation between two genetic regions (e.g., genes) in terms of the aggregated effects of rare variants. In contrast, LD statistic can only be calculated for two variants. Specifically, using our notations, the LD between the u-th variant of Gene 1 and the v-th variant of Gene 2 is defined as

$$LD_{(u,v)} = \frac{\left[\frac{1}{n}\sum_i X_{i1u}X_{i2v} - \frac{1}{n^2}(\sum_i X_{i1u})(\sum_i X_{i2v})\right]^2}{\frac{\sum_i X_{i1u}}{n}(1-\frac{\sum_i X_{i1u}}{n})\frac{\sum_i X_{i2v}}{n}(1-\frac{\sum_i X_{i2v}}{n})}. \tag{1.7}$$

The above definitions of cLD and LD are based on the r^2 format. Alternative, one may define the cLD analogue of D' below:

$$D' = \frac{D}{D_{max}} = \frac{\frac{1}{n}\sum_i X_{i1}X_{i2} - \frac{1}{n^2}(\sum_i X_{i1})(\sum_i X_{i2})}{D_{max}}$$

where

$$D = P_{AB} - P_A P_B = \frac{1}{n}\sum_i X_{i1}X_{i2} - \frac{1}{n^2}(\sum_i X_{i1})(\sum_i X_{i2}).$$

and

$$D_{\max} = \begin{cases} \max\{-P_A P_B, -(1-P_A)(1-P_B)\} \\ = \max\left\{-\frac{1}{n^2}\sum_i X_{i1}\sum_i X_{i2}, -(1-\frac{1}{n}\sum_i X_{i1})(1-\frac{1}{n}\sum_i X_{i2})\right\}, & D \leq 0, \\ \max\{P_A(1-P_B), (1-P_A)P_B\} \\ = \max\left\{\frac{1}{n}\sum_i X_{i1}(1-\frac{1}{n}\sum_i X_{i2}), (1-\frac{1}{n}\sum_i X_{i1})\frac{1}{n}\sum_i X_{i2}\right\}, & D > 0. \end{cases}$$

But in this work, we ONLY focused on the r^2 version, leaving the analysis of D' version to the future work.

Up to now, we didn't have any assumption on the independence of variants. With these

definitions, I gave the rigorous derivations of the distributions of cLD and LD in the following Chapter.

1.2 An example

In this section, we used a toy example to show the calculation of cLD. The example is a small SNV dataset given in Supplementary Table D, an instance of Supplementary Table B.

Table D: Example of SNVs data for cLD calculation

POS	Gene	Info	HG1-1	HG1-2	HG2-1	HG2-2	HG3-1
6034	1	...	1	0	0	0	1
6041	1	...	0	0	0	0	0
6047	1	...	0	0	0	0	0
6057	1	...	0	0	0	1	0
6064	1	...	0	0	0	0	0
7064	2	...	0	0	1	1	0
7124	2	...	1	0	0	0	0
7235	2	...	0	0	0	0	1

From Supplementary Table D, one can form two cumulative gene alleles given in Supplementary Table E (which is an instance of the Supplementary Table C presented previously.

Table E: Example of cumulated gene alleles for cLD calculation

POS	Gene	Info	HG1-1	HG1-2	HG2-1	HG2-2	HG3-1
Gene 1	1	...	1	0	0	1	1
Gene 2	2	...	1	0	1	1	1

As such, based on the formula 1.6 the cLD between Gene 1 and Gene 2 is:

$$cLD = \frac{\left[\frac{1}{n}\sum_i X_{i1}X_{i2} - \frac{1}{n^2}(\sum_i X_{i1})(\sum_i X_{i2})\right]^2}{\frac{\sum_i X_{i1}}{n}(1-\frac{\sum_i X_{i1}}{n})\frac{\sum_i X_{i2}}{n}(1-\frac{\sum_i X_{i2}}{n})} = \frac{(\frac{1}{5}\cdot 3 - \frac{1}{5^2}\cdot 3\cdot 4)^2}{0.6\cdot 0.4\cdot 0.2\cdot 0.8} = 0.375.$$

1.3 Dataset and the population labels

The calculation of cLD in this paper is based on the dataset from the 1000 Genomes Project [1]. This project started in January 2008 and aimed at creating a complete and detailed catalogue of human genetic variations, which can be used for population genetic studies.

The variant call data of the Phase 3 analysis of the 1000 Genome dataset was obtained through The European Bioinformatics Institute's dedicated FTP (http://ftp.1000genomes.ebi.ac.uk) server. The complete variant call dataset was found using the webpage (Announcements — 1000 Genomes (internationalgenome.org)) (This is a sub-page maintained by the 1000 Genome webpage) and downloaded from (Index of /vol1/ftp/release/20130502/ (ebi.ac.uk)).

This dataset has 26 populations: ASW (African Ancestry in SW USA), ACB (African Caribbean in Barbados), BEB (Bengali in Bangladesh), GBR (British from England and Scotland), CDX (Chinese Dai in Xishuangbanna), CLM (Colombian in Medellín, Colombia), ESN (Esan in Nigeria), FIN (Finnish in Finland), GWD (Gambian in Western Division - Mandinka), GIH (Gujarati Indians in Houston, Texas, USA), CHB (Han Chinese in Beijing, China), CHS (Han Chinese South, China), IBS (Iberian populations in Spain), ITU (Indian Telugu in the U.K.), JPT (Japanese in Tokyo, Japan), KHV (Kinh in Ho Chi Minh City, Vietnam), LWK (Luhya in Webuye, Kenya), MSL (Mende in Sierra Leone), MXL (Mexican Ancestry in Los Angeles CA USA), PEL (Peruvian in Lima, Peru), PUR (Puerto Rican in Puerto Rico), PJL (Punjabi in Lahore, Pakistan), STU (Sri Lankan Tamil in the UK), TSI (Toscani in Italia), YRI (Yoruba in Ibadan, Nigeria), CEU (Utah Residents with Northern and Western European ancestry).

Moreover, these 26 populations could be split into five super-populations: EUR (European, 503 samples), EAS (East Asian, 504 samples), AMR (American, 347 samples), SAS (South Asian, 489 samples) and AFR (African, 661 samples). In our studies in the next chapter, we used EUR, EAS and AFR as examples to demonstrate the implementation and performance of cLD. The selection was based on that they are frequently used in other literature and they do not contain substantial population admixtures. Relatives have been

removed to ensure that samples are generally unrelated. More specifically, if an individual's parent or sibling is present in the dataset, then this person is removed from the calculation.

Chapter 2

Asymptotic Properties of cLD

In this chapter, we derived the asymptotic distributions of cLD and LD to show that cLD enjoys lower variance and is more stable than LD. This has been done using two distinct mathematical approaches, outlined below:

First, we used colsed-form derivation of the variance of LD and cLD and used them to calculate their variances using parameters collected from the 1000 Genomes Project data. More specifically, we first modelled the exact distribution of cLD and LD via multinomial random variables using the allele counts, and then used multivariate Central Limit Theory (CLT) and multivariate Delta method to derive the asymptotic distributions of LD and cLD respectively. These results are presented in Sections 2.1 and 2.2. Such results allow us to derive the variances in closed form ready to be calculated. In Section 2.3, we compared the variability of cLD with LD and showed that cLD has a lower variance than LD.

As the above derivation assumes large samples (for the multivariate CLT to be valid), for the cases of limited samples, in section 2.4, we used bootstrap method to show that cLD is more stable than LD in the partial samples. Here the property of being "sable" is reflected by the slimmer distribution (i.e., with lower variability) formed by the bootstrapped samples.

2.1 Derivation of closed-form distribution of LD

2.1.1 Multinomial distribution

With the same notation defined in Chapter 1, we used X_{ijk} to denote the i-th haplotype of the j-th gene and the k-th variant. Without loss of generality, we assumed that SNV1 and SNV2 come from Gene 1 and Gene 2 respectively. The data for two SNVs are denoted in Supplementary Table F. In such a case, each haplotype has a pair of SNVs (SNV1 is the u-th variant from Gene 1 while SNV2 is the v-th variant from Gene 2), and the i-th pair (X_{i1u}, X_{i2v}), $i = 1, \ldots, n$, on (SNV1, SNV2) can take possible values $(1,0)$, $(1,1)$, $(0,1)$ and $(0,0)$. Further, these n pairs can be treated as i.i.d. following a multinomial distribution given in Supplementary Table G. This table shows a multinomial distribution for the i-th haplotype, where O_i's denote the frequency for each possible pair value. The third column lists the counts for each possible outcome of the pair (X_{i1u}, X_{i2v}) throughout the entire dataset, and the fourth column represents the corresponding population probabilities of the multinomial distribution. Intuitively the population parameters $p_1, \ldots, p_4$ can be estimated by sample fractions, i.e.

$$\hat{p}_i = O_i/n, \quad i = 1, \ldots, 4.$$

Table F: Data of two SNVs

POS	Gene	Info	HG01	HG02	HG03	HG04	HG05	...	MAF
SNV1	1	...	X_{11u}	X_{21u}	X_{31u}	X_{41u}	X_{51u}	...	p_{11}
SNV2	2	...	X_{12v}	X_{22v}	X_{32v}	X_{42v}	X_{52v}	...	p_{21}

Table G: Multinomial modelling of two SNVs

X_{i1u}	X_{i2v}	Count	Probability
1	1	O_1	p_1
0	1	O_2	p_2
1	0	O_3	p_3
0	0	$O_4 = n - O_1 - O_2 - O_3$	$p_4 = 1 - p_1 - p_2 - p_3$

With these preparations of defining notations, we estimated the LD $((r^2))$ and then derive the asymptotic distribution of the resulted estimator. Firstly, the LD can be rewritten in terms of current parameters as

$$LD_{(u,v)} = \left[\frac{p_{AB} - p_A p_B}{\sqrt{p_A(1-p_A)p_B(1-p_B)}}\right]^2 = \left[\frac{p_1 - (p_1+p_3)(p_1+p_2)}{\sqrt{(p_1+p_3)(p_2+p_4)(p_1+p_2)(p_3+p_4)}}\right]^2. \quad (2.1)$$

Then the LD can be estimated by plugging-in the estimators using the counts O_x, $(x = 1, 2, 3, 4)$:

$$\begin{aligned} \widehat{LD}_{(u,v)} &= \left[\frac{\hat{p}_1 - (\hat{p}_1+\hat{p}_3)(\hat{p}_1+\hat{p}_2)}{\sqrt{(\hat{p}_1+\hat{p}_3)(\hat{p}_2+\hat{p}_4)(\hat{p}_1+\hat{p}_2)(\hat{p}_3+\hat{p}_4)}}\right]^2 \\ &= \frac{\left[\frac{O_1}{n} - \frac{1}{n^2}(O_1+O_2)(O_1+O_3)\right]^2}{\frac{O_1+O_3}{n}(1-\frac{O_1+O_3}{n})\frac{O_1+O_2}{n}(1-\frac{O_1+O_2}{n})} \\ &= \frac{(O_1O_4 - O_2O_3)^2}{(O_1+O_2)(O_1+O_3)(O_2+O_4)(O_3+O_4)}. \end{aligned} \quad (2.2)$$

Though both LD and $\widehat{LD}$ are calculated for fixed variant pair (u, v), for notation simplicity we remove their dependence on (u, v) in the subscripts and let $LD(\boldsymbol{p}) = LD_{(u,v)}$ and $LD(\boldsymbol{O}/n) = \widehat{LD}_{(u,v)}$, where $\boldsymbol{p} = (p_1, p_2, p_3, p_4)^\top$ and $\boldsymbol{O} = (O_1, O_2, O_3, O_4)^\top$.

The mean of the estimator $LD(\boldsymbol{O}/n)$ is thus given by:

$$E[LD(\boldsymbol{O}/n)] = \sum_{\Omega_n} \frac{(O_1O_4 - O_2O_3)^2}{(O_1+O_2)(O_1+O_3)(O_2+O_4)(O_3+O_4)} \cdot p(O_1, O_2, O_3),$$

where $\Omega_n = \{(O_1, O_2, O_3) : O_i \in \mathbb{N} \cup \{0\}, O_i \leq n, O_1 + O_2 + O_3 \leq n\}$ and

$$p(O_1, O_2, O_3) = \frac{n!}{O_1!O_2!O_3!O_4!} {p_1}^{O_1} {p_2}^{O_2} {p_3}^{O_3} {p_4}^{O_4}.$$

The variance of $LD(\boldsymbol{O}/n)$ is $Var(LD(\boldsymbol{O}/n)) = E\left[(LD(\boldsymbol{O}/n))^2\right] - E^2[LD(\boldsymbol{O}/n)]$.

It is known that when sample size n is reasonably large, the calculation of multinomial probabilities are very tedious, thus the expressions for the mean and variance of $LD(\boldsymbol{O}/n)$

given above are not practical. To solve this problem, we next used the multivariate CLT and Delta method to approximate the exact distribution of $LD(\boldsymbol{O}/n)$ when n is large.

2.1.2 Asymptotic normality approximation of LD

For notation simplicity, we defined events E_1 as $(X_{1u}, X_{2v}) = (1,1)$ on (SNV1, SNV2), E_2 as $(X_{1u}, X_{2v}) = (0,1)$ on (SNV1, SNV2), E_3 as $(X_{1u}, X_{2v}) = (1,0)$ on (SNV1, SNV2), and E_4 as $(X_{1u}, X_{2v}) = (0,0)$ on (SNV1, SNV2). Then

$$\boldsymbol{O} = \begin{pmatrix} O_1 \\ O_2 \\ O_3 \\ O_4 \end{pmatrix} = \sum_{i=1}^{n} \begin{pmatrix} I(E_1 \text{ occurs on the } i\text{−th haplotype}) \\ I(E_2 \text{ occurs on the } i\text{−th haplotype}) \\ I(E_3 \text{ occurs on the } i\text{−th haplotype}) \\ I(E_4 \text{ occurs on the } i\text{−th haplotype}) \end{pmatrix} = \sum_{i=1}^{n} \boldsymbol{I_i},$$

where $\boldsymbol{I_i} = (I_{i1}, I_{i2}, I_{i3}, I_{i4})^\top$ and

$$I_{ij} = I(E_j \text{ occurs on the } i\text{−th haplotype}), \quad j = 1,2,3,4, \ i = 1, \ldots, n.$$

As a result, $\boldsymbol{O} \sim Multinom(n; \boldsymbol{p})$ with $\boldsymbol{p} = (p_1, p_2, p_3, p_4)^\top$. Since these n samples are independent, by multivariate central limit theorem (CLT), we have the asymptotic normality of $\frac{\boldsymbol{O}}{n}$ given by

$$\sqrt{n}\left(\frac{\boldsymbol{O}}{n} - \boldsymbol{p}\right) \xrightarrow{\mathcal{L}} N(\boldsymbol{0}, \Sigma),$$

where $\xrightarrow{\mathcal{L}}$ denotes convergence in distribution and the asymptotic covariance matrix $\Sigma = Cov(\boldsymbol{I_1}) = (\sigma_{jl})$. Straightforward calculation gives

$$\sigma_{jl} = E[I_{1j}I_{1l}] - E[I_{1j}]E[I_{1l}] = P(I_{1j} = I_{1l} = 1) - p_j p_l = \begin{cases} -p_j p_l, & j \neq l, \\ p_j - p_j p_l, & j = l. \end{cases}$$

Thus

$$\Sigma = \begin{pmatrix} p_1 - p_1^2 & -p_1p_2 & -p_1p_3 & -p_1p_4 \\ -p_2p_1 & p_2 - p_2^2 & -p_2p_3 & -p_2p_4 \\ -p_3p_1 & -p_3p_2 & p_3 - p_3^2 & -p_3p_4 \\ -p_4p_1 & -p_4p_2 & -p_4p_3 & p_4 - p_4^2 \end{pmatrix} = \boldsymbol{D_p} - \boldsymbol{p}\boldsymbol{p}^\top, \tag{2.3}$$

where $\boldsymbol{D_p}$ is the diagonal matrix with diagonal elements $p_1, \ldots, p_4$. Note that Σ is singular, since the sum of each row of Σ gives 0. As a result, when sample size n is large, we have the approximated distributions of $\boldsymbol{O}/n$ and $\boldsymbol{O}$ given by

$$\frac{\boldsymbol{O}}{n} \sim AN\left(\boldsymbol{p}, \frac{\Sigma}{n}\right), \quad \boldsymbol{O} \sim AN\left(n\boldsymbol{p}, n\Sigma\right),$$

where 'AN' stands for Asymptotic Normal.

In order to derive the asymptotic distributions of cLD and LD, first note that the LD given in (1.7) is a function of $\boldsymbol{O}$, thus the multivariate Delta method applied to the asymptotic normality of $\boldsymbol{O}$ would give us the asymptotic normality of LD. Let $\boldsymbol{J}_{LD} = \left[\frac{\partial LD(\boldsymbol{O}/n)}{\partial \boldsymbol{O}}\right]\Big|_{\boldsymbol{O}=n\boldsymbol{p}}$ be the 1×4 Jacobian Matrix of $LD(\boldsymbol{O}/n)$, with the $(1, j)$-th element being $\left(\frac{\partial LD(\boldsymbol{O}/n)}{\partial O_j}\right)|_{\boldsymbol{O}=n\boldsymbol{p}}$, where

$$\frac{\partial LD(\boldsymbol{O}/n)}{\partial O_1} = \frac{(O_1O_4 - O_2O_3)[2O_2O_3(O_1 + O_4) + (O_2 + O_3)(O_1O_4 + O_2O_3)]}{(O_1 + O_2)^2(O_1 + O_3)^2(O_2 + O_4)(O_3 + O_4)},$$

$$\frac{\partial LD(\boldsymbol{O}/n)}{\partial O_2} = -\frac{(O_1O_4 - O_2O_3)[2O_1O_4(O_2 + O_3) + (O_1 + O_4)(O_1O_4 + O_2O_3)]}{(O_1 + O_2)^2(O_1 + O_3)(O_2 + O_4)^2(O_3 + O_4)},$$

$$\frac{\partial LD(\boldsymbol{O}/n)}{\partial O_3} = -\frac{(O_1O_4 - O_2O_3)[2O_1O_4(O_2 + O_3) + (O_1 + O_4)(O_1O_4 + O_2O_3)]}{(O_1 + O_2)(O_1 + O_3)^2(O_2 + O_4)(O_3 + O_4)^2},$$

$$\frac{\partial LD(\boldsymbol{O}/n)}{\partial O_4} = \frac{(O_1O_4 - O_2O_3)[2O_2O_3(O_1 + O_4) + (O_2 + O_3)(O_1O_4 + O_2O_3)]}{(O_1 + O_2)(O_1 + O_3)(O_2 + O_4)^2(O_3 + O_4)^2}.$$

Then multivariate Delta method gives the asymptotic distribution of $LD(\boldsymbol{O}/n)$ as

$$LD(\boldsymbol{O}/n) - LD(\boldsymbol{p}) \sim AN\left(0, n\boldsymbol{J}_{LD}\Sigma\boldsymbol{J}_{LD}^\top\right), \tag{2.4}$$

where Σ is given in (2.3).

The derived asymptotic distribution of $LD(\boldsymbol{O}/n)$ given in (2.4) can be used for various downstream tasks. For instance, one can assess the significance of LD using the p-value calculated by the distribution.

In this work, the use of the above distribution is to estimate the asymptotic variance of $LD(\boldsymbol{O}/n)$, $n\boldsymbol{J}_{LD}\Sigma\boldsymbol{J}_{LD}^{\top}$, which can not be used directly in practice due to the unknown $\boldsymbol{p}$ involved. Thus to estimate $Var(\widehat{LD})$, intuitively we have to use plug-in method by replacing the unknowns by the observed allele counts. More specifically, we let $\widehat{\Sigma}$ denote the Σ in (2.3) with $\boldsymbol{p}$ replaced by $\boldsymbol{O}/n$ and let $\widehat{\boldsymbol{J}}_{LD} = \frac{\partial LD(\boldsymbol{O}/n)}{\partial \boldsymbol{O}}$, then when sample size n is large, the variance of $LD(\boldsymbol{O}/n)$ can be estimated by

$$\widehat{Var}(LD(\boldsymbol{O}/n)) = n\widehat{\boldsymbol{J}}_{LD}\widehat{\Sigma}\widehat{\boldsymbol{J}}_{LD}^{\top}. \tag{2.5}$$

2.2 Derivation of closed-form distribution of cLD

The asymptotic distribution and estimated variance of cLD could be similarly derived as to those of LD in Section 2.1. The difference between cLD and LD is that they are functions of different data. LD is calculated on two SNVs, while cLD is calculated on aggregated effect of SNVs in two genes. That said, the forms of SNV pairs and gene pairs are mathematically similar and can be modelled similarly. Here, we extended the derivations for LD to cLD with some non-essential modifications.

For n independent pairs (X_{i1}, X_{i2}), $i = 1, \ldots, n$, observed on two genes (Gene 1, Gene 2), they can be treated as i.i.d. following a multinomial distribution given in Supplementary Table H, along with frequencies M_i's for each possible pair value.

Firstly, the cLD (r^2) can be rewritten in terms of current parameters as

$$cLD := cLD(\boldsymbol{q}) = \left[\frac{q_1 - (q_1+q_3)(q_1+q_2)}{\sqrt{(q_1+q_3)(q_2+q_4)(q_1+q_2)(q_3+q_4)}}\right]^2. \tag{2.6}$$

Table H: Multinomial modelling of two gene lines

X_{i1}	X_{i2}	Count	Probability
1	1	M_1	q_1
0	1	M_2	q_2
1	0	M_3	q_3
0	0	$M_4 = n - M_1 - M_2 - M_3$	$q_4 = 1 - q_1 - q_2 - q_3$

Then the cLD can be estimated by the plug-in estimator

$$\begin{aligned} \widehat{cLD} := cLD(\boldsymbol{M}/n) &= \frac{\left[\frac{M_1}{n} - \frac{1}{n^2}(M_1+M_2)(M_1+M_3)\right]^2}{\frac{M_1+M_3}{n}(1-\frac{M_1+M_3}{n})\frac{M_1+M_2}{n}(1-\frac{M_1+M_2}{n})} \\ &= \frac{(M_1M_4 - M_2M_3)^2}{(M_1+M_2)(M_1+M_3)(M_2+M_4)(M_3+M_4)}. \end{aligned} \tag{2.7}$$

where $\boldsymbol{M} = (M_1, M_2, M_3, M_4)^\top$ and $\boldsymbol{q} = (q_1, q_2, q_3, q_4)^\top$. Following exactly the same steps as in Section 2.1 for LD, we have

$$cLD(\boldsymbol{M}/n) - cLD(\boldsymbol{q}) \sim AN\left(0, n\boldsymbol{J}_{cLD}\boldsymbol{Q}\boldsymbol{J}_{cLD}^\top\right), \tag{2.8}$$

where

$$\boldsymbol{Q} = \begin{pmatrix} q_1 - q_1^2 & -q_1q_2 & -q_1q_3 & -q_1q_4 \\ -q_2q_1 & q_2 - q_2^2 & -q_2q_3 & -q_2q_4 \\ -q_3q_1 & -q_3q_2 & q_3 - q_3^2 & -q_3q_4 \\ -q_4q_1 & -q_4q_2 & -q_4q_3 & q_4 - q_4^2 \end{pmatrix}, \tag{2.9}$$

$$\boldsymbol{J}_{cLD} = \left[\frac{\partial cLD(\boldsymbol{M}/n)}{\partial \boldsymbol{M}}\right]\Bigg|_{\boldsymbol{M}=n\boldsymbol{q}} = \left(\frac{\partial cLD(\boldsymbol{M}/n)}{\partial M_1}, \frac{\partial LD(\boldsymbol{M}/n)}{\partial M_2}, \frac{\partial cLD(\boldsymbol{M}/n)}{\partial M_3}, \frac{\partial cLD(\boldsymbol{M}/n)}{\partial M_4}\right)\Bigg|_{\boldsymbol{M}=n\boldsymbol{q}}.$$

Similar to the situation in the last section, from (2.8) we see that the asymptotic variance of $cLD(\boldsymbol{M}/n)$ is $n\boldsymbol{J}_{cLD}\boldsymbol{Q}\boldsymbol{J}_{cLD}^\top$, which can not be used directly in practice due to the unknown $\boldsymbol{q}$ involved. Thus to estimate $Var(\widehat{cLD})$, we let $\widehat{\boldsymbol{Q}}$ denote the $\boldsymbol{Q}$ in (2.9) with $\boldsymbol{q}$ replaced by $\boldsymbol{M}/n$ and let $\widehat{\boldsymbol{J}}_{cLD} = \frac{\partial cLD(\boldsymbol{M}/n)}{\partial \boldsymbol{M}}$, then when sample size n is large, the variance of

$\widehat{cLD} = cLD(\boldsymbol{M}/n)$ can be estimated by

$$\widehat{Var}(cLD(\boldsymbol{M}/n)) = n\widehat{\boldsymbol{J}}_{cLD}\widehat{\boldsymbol{Q}}\widehat{\boldsymbol{J}}_{cLD}^{\top}. \tag{2.10}$$

2.3 Comparison of variances using closed-form distributions

2.3.1 Comparison of variances between cLD and LD

In Section 2.1 and 2.2, we have derived the asymptotic variances of $cLD(\boldsymbol{M}/n)$ and $LD(\boldsymbol{O}/n)$ and provided their variance estimates which can be calculated directly from data. In this section, we conducted the comparison between cLD and LD in terms of their variability by plugging-in the frequency estimates using the 1000 Genomes Project data.

First, we randomly sampled 1000 gene pairs from the 1000 Genomes Project data. Second, we calculated $Var(cLD(\boldsymbol{M}/n))$ for each gene pair by their estimated cMAF in the real data; and then obtain the average value $\overline{Var}(cLD(\boldsymbol{M}/n))$ over all gene pairs in our sample. Third, similarly, within each gene pair, we calculated the $Var(LD(\boldsymbol{O}/n))$ for each rare SNV pair, and we define the variance of LD of a gene pair as the average value of $Var(LD(\boldsymbol{O}/n))$ over all rare SNV pairs within that gene pair. As such, we obtained the average value of variance $\overline{Var}(cLD(\boldsymbol{M}/n))$ and $\overline{Var}(LD(\boldsymbol{O}/n))$ over the randomly sampled gene pairs.

However, as cLD is not at the same scale as LD, the direct comparison between the variances may be unfair. In the above setting, as there are many pairs of SNVs between a pair of genes (therefore many LD values) whereas only one cLD between a pair of genes, the LD has been highly averaged, leading to lower variance. In contrast, if we do not conduct such within-gene average, and only conduct the sum or taking a random pair of SNVs, it is unfair for LD.

To elaborate the above intuition mathematically, let us look at the formula of variances:

$$Var(X) = E[X - E(X)]^2$$

where X is a random variable. If the scale of X is a small value, then the variance could also be also small. Thus, in order to compare the stability of them, we mush use mean value to re-scale $cLD(\boldsymbol{M}/n)$ and $LD(\boldsymbol{O}/n)$.

Based on the above rationale, we used the following formulas to conduct the re-scaling before the comparison:

$$cLD_{scaled} = \frac{cLD(\boldsymbol{M}/n)}{mean\ value\ of\ cLD(\boldsymbol{M}/n)}$$

and

$$LD_{scaled} = \frac{LD(\boldsymbol{O}/n)}{mean\ value\ of\ LD(\boldsymbol{O}/n)}.$$

Where the mean values of LD and cLD are calculated over all gene pairs in our sample. Thus we can compare the scaled variance of LD and cLD:

$$\overline{Var}(cLD)_{scaled} = \frac{\overline{Var}(cLD(\boldsymbol{M}/n))}{(mean\ value\ of\ cLD(\boldsymbol{M}/n))^2}$$

and

$$\overline{Var}(LD)_{scaled} = \frac{\overline{Var}(LD(\boldsymbol{O}/n)))}{(mean\ value\ of\ LD(\boldsymbol{O}/n))^2}.$$

The result from 1000 Genomes Project data shows:

$$\overline{Var}(cLD)_{scaled} \approx 1.3 \times 10^8 \ll 3.6 \times 10^{14} \approx \overline{Var}(LD)_{scaled} \tag{2.11}$$

Evidently, the variance of cLD is **six** magnitude lower than the one of LD, indicating it is a way stabler statistic than standard LD.

Moreover, we wanted to stratify the comparison of variance by the value of cMAF. To do

this, we split the gene pairs into 4 groups according to the smaller cMAF of the two genes (in the pair to calculate LD and cLD). The cMAF intervals are set to be [0, 0.05], [0.05, 0.1], [0.1, 0.2] and [0.2, 0.4]. After separating gene pairs into 4 groups, we calculated the average variance of cLD using the closed-form formula over gene pairs for each group. Thus we have 4 values for Var(cLD), matching the 4 cMAF groups. Similarly, for each cMAF group, we used the same method in this section to calculate the average Var(LD). Finally, we calculate the ratio for each cMAF group:

$$Ratio \;=\; \frac{\overline{Var}(cLD)}{\overline{Var}(LD)}$$

The results of EUR, AFR and EAS population are shown in Supplementary Fig. A (a), Supplementary Fig. B(a) and Supplementary Fig. C(a), respectively. They show the same pattern that, in all populations, cLD is more stable at all cMAF spectrum, with the most significant effect at the low cMAF end.

2.3.2 Asymptotic distribution of cLD changes in Case and Control groups

Suppose the cLD between gene i and gene j in the case group is $cLD_{i,j}^{case}$, and the corresponding estimator is $\widehat{cLD}_{i,j}^{case}$. Similarly, we can define $cLD_{i,j}^{control}$ and its estimator $\widehat{cLD}_{i,j}^{control}$. From 2.2, we can derive the asymptotic distribution of $\widehat{cLD}_{i,j}^{case}$ is:

$$\widehat{cLD}_{i,j}^{case} - cLD_{i,j}^{case} \sim AN(0, n_{case}\boldsymbol{J}_{cLD_{i,j};case}\boldsymbol{Q}_{i,j;case}\boldsymbol{J}_{cLD_{i,j};case}^{\top})$$

Where n_{case} is the case sample size, $\boldsymbol{J}_{cLD_{i,j};case}$ follows the same calcualtion from 2.2 using the case population data, similarly for $\boldsymbol{Q}_{i,j;case}$. One may easily use the case sample data to obtain the respoective estimators $\hat{\boldsymbol{J}}_{cLD_{i,j};case}$ and $\hat{\boldsymbol{Q}}_{i,j;case}$. Thus, the approximate distribution is:

$$\widehat{cLD}_{i,j}^{case} - cLD_{i,j}^{case} \sim AN(0, n_{case}\widehat{\boldsymbol{J}}_{cLD_{i,j};case}\widehat{\boldsymbol{Q}}_{i,j;case}\widehat{\boldsymbol{J}}^{\top}_{cLD_{i,j};case})$$

In the same way, the approximate asymptotic distribution of $\widehat{cLD}_{i,j}^{control}$ is:

$$\widehat{cLD}_{i,j}^{control} - cLD_{i,j}^{control} \sim AN(0, n_{control}\widehat{\boldsymbol{J}}_{cLD_{i,j};control}\widehat{\boldsymbol{Q}}_{i,j;control}\widehat{\boldsymbol{J}}^{\top}_{cLD_{i,j};control})$$

Where $n_{control}$ is the control sample size, $\widehat{\boldsymbol{J}}_{cLD_{i,j};control}$ and $\widehat{\boldsymbol{Q}}_{i,j;control}$ are the estimators of $\boldsymbol{J}_{cLD_{i,j};control}$ and $\boldsymbol{Q}_{i,j;control}$ respectively.

Since we are interested in the difference between $cLD_{i,j}^{case}$ and $cLD_{i,j}^{control}$ (i.e. $\Delta cLD_{i,j}^{case,control}$), we can use the sample data to obtain the estimator $\widehat{cLD}_{i,j}^{case} - \widehat{cLD}_{i,j}^{control} = \Delta\widehat{cLD}_{i,j}^{case,control}$. Now, if we assume the $\widehat{cLD}_{i,j}^{case}$ and $\widehat{cLD}_{i,j}^{control}$ are independent from each other, then the approximate asymptotic distribution of $\Delta\widehat{cLD}_{i,j}^{case,control}$ is:

$$\begin{aligned}\Delta\widehat{cLD}_{i,j}^{case,control} &\sim AN(\Delta cLD_{i,j}^{case,control}, n_{case}\widehat{\boldsymbol{J}}_{cLD_{i,j};case}\widehat{\boldsymbol{Q}}_{i,j;case}\widehat{\boldsymbol{J}}^{\top}_{cLD_{i,j};case}+\\ &n_{control}\widehat{\boldsymbol{J}}_{cLD_{i,j};control}\widehat{\boldsymbol{Q}}_{i,j;control}\widehat{\boldsymbol{J}}^{\top}_{cLD_{i,j};control})\end{aligned}$$

If we assume that there is no difference between case and control groups, the approximate asymptotic distribution of $\Delta\widehat{cLD}_{i,j}^{case,control}$ is:

$$\begin{aligned}\Delta\widehat{cLD}_{i,j}^{case,control} &\sim AN(0, n_{case}\widehat{\boldsymbol{J}}_{cLD_{i,j};case}\widehat{\boldsymbol{Q}}_{i,j;case}\widehat{\boldsymbol{J}}^{\top}_{cLD_{i,j};case}+\\ &n_{control}\widehat{\boldsymbol{J}}_{cLD_{i,j};control}\widehat{\boldsymbol{Q}}_{i,j;control}\widehat{\boldsymbol{J}}^{\top}_{cLD_{i,j};control}).\end{aligned}$$

2.4 Estimating stability by Bootstrapped distributions

In Section 2.3, we used the closed-form formula to compare the variance of scaled cLD and LD, and concluded that cLD is stabler. To conduct such comparisons thoroughly, in this section, we used a different strategy, the Bootstrap method, to compare the variance of cLD

and LD. We are still using the 1000 Genomes Project data in the calculation.

The idea of this comparison is that we randomly sampled half of the haplotypes in a given population and calculated the average cLD and average LD over the gene pairs within a cMAF group, and then we repeat this procedure 500 times. After that, we have 500 values of cLD and LD. Base on these cLDs and LDs from 500 iterations, we can use the same method in Section 2.3 to re-scale the cLD and LD, and then calculate the distribution of scaled cLD and LD. In order to study the effect of cMAF on the distribution, we separate the gene pairs into 10 groups according to the two cMAFs of the two genes in the gene pair. More specifically, the steps are as follows.

Step 1: Randomly sample 1000 genes from the 1000 Genomes Project data.

Step 2: Separate genes into 4 bins according to these threshold:

$$\begin{aligned}
&Bin\ 1:\ cMAF\ in\ [0.2,\ 0.4]\\
&Bin\ 2:\ cMAF\ in\ [0.1,\ 0.2]\\
&Bin\ 3:\ cMAF\ in\ [0.05,\ 0.1]\\
&Bin\ 4:\ cMAF\ in\ [0,\ 0.05]
\end{aligned}$$

Then pair-wisely, the genes were paired into 10 groups:

$$\begin{aligned}
&(Bin\ 1, Bin\ 1),\ (Bin\ 1, Bin\ 2),\ (Bin\ 1, Bin\ 3),\ (Bin\ 1, Bin\ 4),\\
&(Bin\ 2, Bin\ 2),\ (Bin\ 2, Bin\ 3),\ (Bin\ 2, Bin\ 4),\\
&(Bin\ 3, Bin\ 3),\ (Bin\ 3, Bin\ 4),\ and\\
&(Bin\ 4, Bin\ 4).
\end{aligned}$$

Then we repeated the following Step 3 to Step 5 for 500 times.

Step 3: Randomly sampled half of the haplotypes in the given population (AFR, EAS or

EUR).

Step 4: For each gene pair, we calculated the cLD and LD. Similar to Section 2.3, we define the LD of a gene pair as the average value of $LD(\boldsymbol{O}/n)$ over all rare SNV pairs within that gene pair.

Step 5: In each iteration, calculated the avarage cLD (denoted as cLD_{ij}) and LD (denoted as LD_{ij}) over the gene pairs in each group, where i denotes the i-th iteration (i = 1, 2, ..., 500) and j denotes the group number (j = 11, 12 ,13, 14, 22, 23, 24, 33, 34, 44).

Step 6: Lastly, base on cLD_{ij} and LD_{ij} (i = 1,2...500 and j = 11, 12 ,13, ..., 44), we obtained the density of cLD and LD for 10 cMAF groups. To be more specific, we are using kernel estimation to smooth the distribution for the visualization.

The results of EUR, AFR and EAS population are shown in Supplementary Fig. A (b), Supplementary Fig. B (b) and Supplementary Fig. C (b), respectively. These results show that cLD has a way slimmer bootstrapped distribution than LD, further evidencing the stability of cLD over LD.

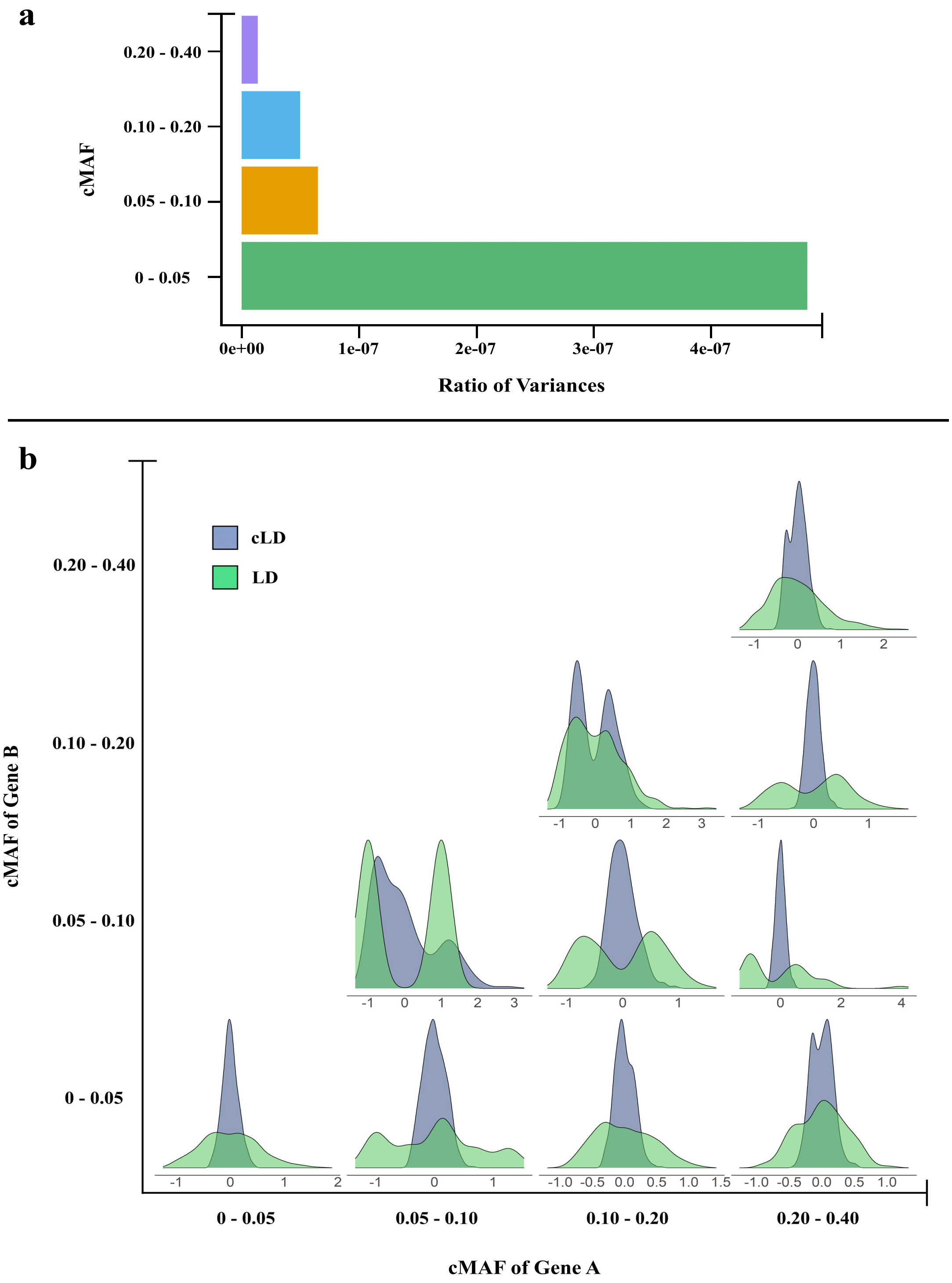

Fig. A: Stability of cLD and LD revealed by closed-form variance calculation and bootstrapped resampling. The EUR population is shown. a) The gene pairs were split into four different bins based on the cMAF values, i.e., < 0.05, 0.05 - 0.1, 0.1 - 0.2, and 0.2-0.4, which has been shown in the y-axis. The x-axis is the ratio between the variance of LD and cLD. b) Probability density distribution of cLD and LD by generating bootstrapped samples.

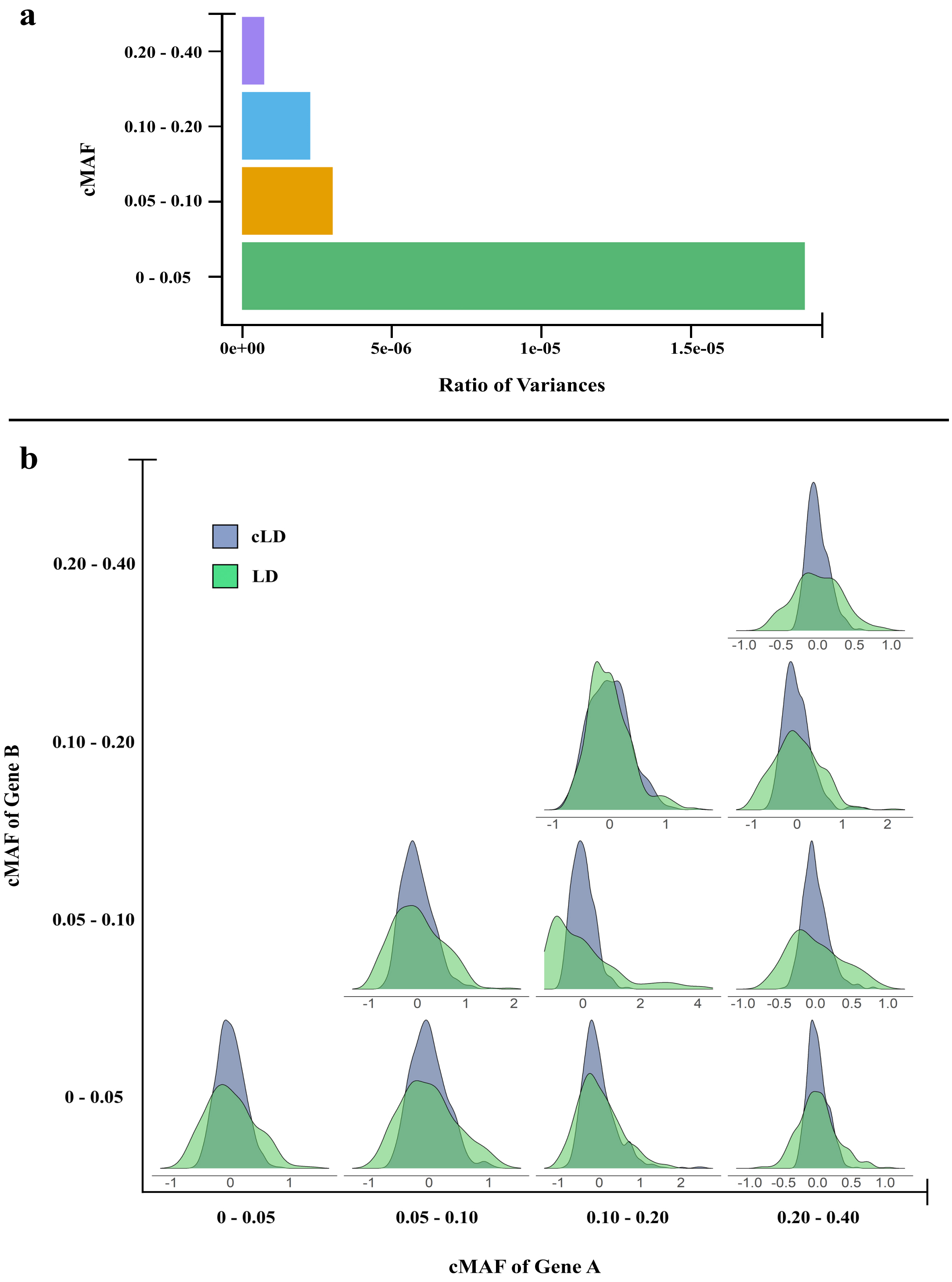

Fig. B: Stability of cLD and LD revealed by closed-form variance calculation and bootstrapped resampling. The AFR population is shown. a) The gene pairs were split into four different bins based on the cMAF values, i.e., < 0.05, 0.05 - 0.1, 0.1 - 0.2, and 0.2-0.4, which has been shown in the y-axis. The x-axis is the ratio between the variance of LD and cLD. b) Probability density distribution of cLD and LD by generating bootstrapped samples.

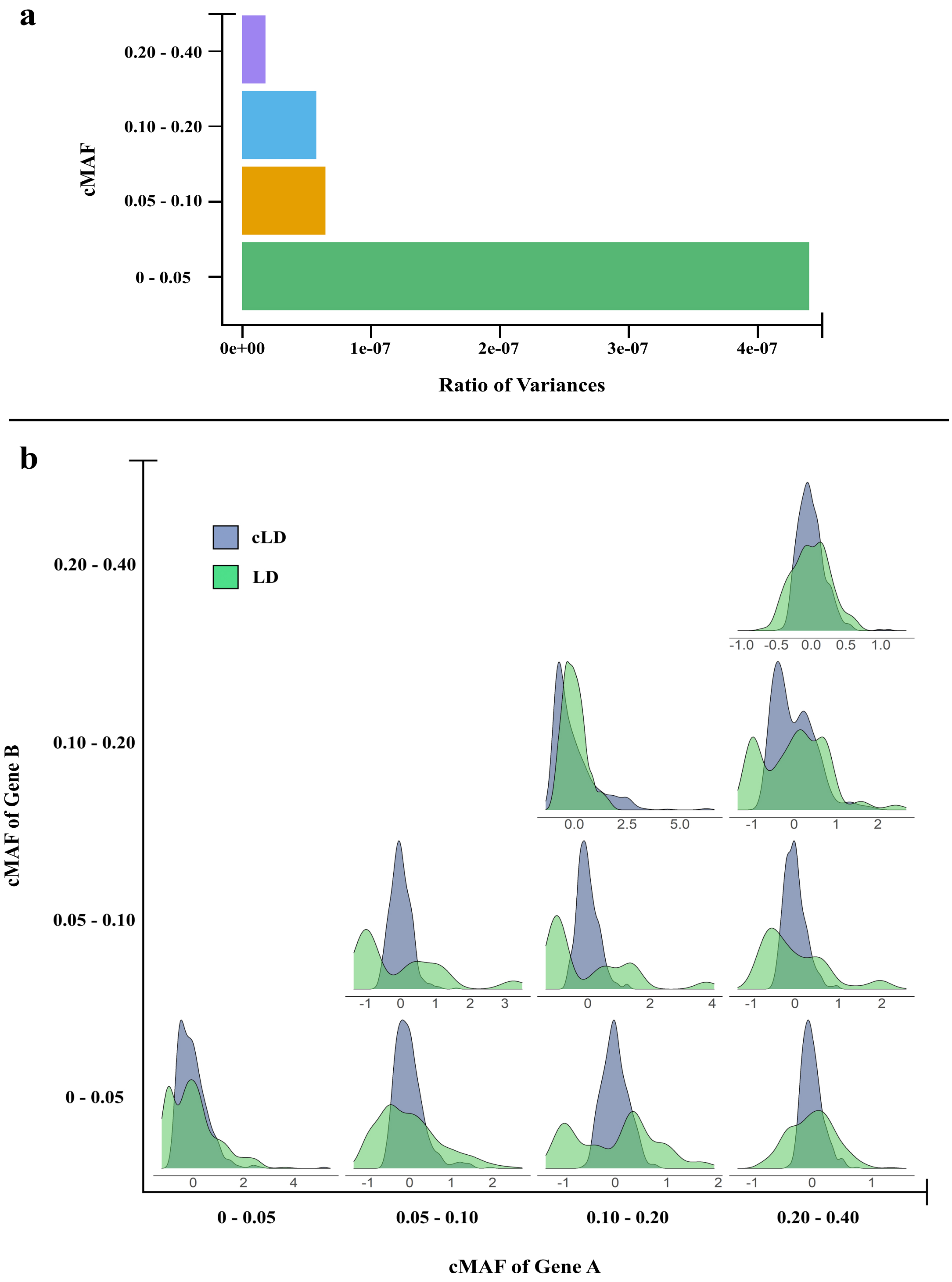

Fig. C: Stability of cLD and LD revealed by closed-form variance calculation and bootstrapped resampling. The EAS population is shown. a) The gene pairs were split into four different bins based on the cMAF values, i.e., < 0.05, 0.05 - 0.1, 0.1 - 0.2, and 0.2-0.4, which has been shown in the y-axis. The x-axis is the ratio between the variance of LD and cLD. b) Probability density distribution of cLD and LD by generating bootstrapped samples.

Chapter 3

Applying cLD to real sequence data

In this chapter, to show the practical use of cLD in capturing genetic interactions, we calculated cLD using phased version of the 1000 Genomes Project data described in Chapter 1 and characterized its enrichment based on known interactions. These interactions include both 3D spatial genomic interactions (assessed by the Hi-C experiments) and the four popular interaction databases.

The rest of this chapter is organized as follows. Because such disequilibrium statistics (including both cLD and LD) in general decrease as the distance between the two loci increases, in Section 3.1, we defined the bins according to the distance between the pairs as the units of the analyses in this chapter. We then use cLD to assess the enrichment of high cLD pairs in the 3D interacting regions. In contrast to the negative results reported previously using standard LD [2], we indeed discovered the higher cLD in 3D interaction regions in Section 3.2. Next, we applied cLD to four interaction databases Intact, Reactom, MINT & Biogrid and discovered that cLD is also enriched in gene pairs with known interactions in Section 3.3. To rigorously assess the statistical significance of the above mentioned enrichment in both 3D and gene-gene interactions, we designed the use of the Fisher's Exact test and Mantel-Haenszel test to quantify the p-values of the comparisons in the previous sections. Finally, in Section 3.5, we provided the details of enrichment analysis that shows

the higher proportion of pairs of genes in 3D interaction regions.

3.1 Gene pairs grouped by distance bins

To give one single number of the location of a gene, we calculated the mean value of the positions of all SNVs in this gene. Then for every pairs of genes, the difference of their locations (as calculated above) will be their distance. Based on the distances of the gene pairs, they are split in to 13 groups using the cut points below:

$35 \cdot 10^3 = 35Kb$, $70 \cdot 10^3 = 70Kb$, $140 \cdot 10^3 = 140Kb$, $280 \cdot 10^3 = 280Kb$, $560 \cdot 10^3 = 560Kb$, $1.12 \cdot 10^6 = 1.12Mb$, $2.24 \cdot 10^6 = 2.24Mb$, $4.48 \cdot 10^6 = 4.48Mb$, $8.96 \cdot 10^6 = 8.96Mb$, $17.92 \cdot 10^6 = 17.92Mb$, $35.84 \cdot 10^6 = 35.84Mb$, $71.6 \cdot 10^6 = 71.6Mb$ and $143.2 \cdot 10^3 = 143.2Mb$.

The above grouping applies to all the analysis in this chapter.

3.2 LD and cLD in 3D interacting regions assessed by Hi-C experiments

There are a series of emerging molecular biology methods for analyzing the spatial organization of chromatin in a cell using next-generation sequencing instruments. Using these techniques, researchers can quantify the interactions between loci that are nearby in 3D space, but may not be close to each other in the linear genome [3]. Among these techniques, Hi-C is a frequently used techniques assessing 3D structure in a high resolution.

There was a widely spread expectation that the 3D genomic interaction in the form of chromatin contact may leave a footprint in the form of genetic LD. Motivated by such discussions, Whalen and Pollard have measured the LD using r^2 based on the common variants (MAF > 0.05) in 1000 Genomes Project data and reported negative results stating that genetic LD maps are not overlapping with the 3D contact map. However, in this section, by re-analyzing the same genotype data and the Hi-C revealed 3D contact map

in the developing brain using cLD and rare variants, we revealed that the 3D chromatin interactions did leave genetic footprints in the form of higher cLD in pairs of genes that are in the contacting Hi-C regions.

Since cLD statistic is designed to capture the association between genes, we expect the cLD between gene pairs that are in the 3D-interaction regions to be higher than the cLD between gene pairs that do not. To demonstrate this, we compare the average cLD of gene pairs in 3D-interaction regions against the average cLD of gene-pairs not in 3D-interaction regions. For an appropriate comparison, we stratify the data using the different distance bins defined in section 3.1.

The distribution of cLD values in the 3D Hi-C dataset are displayed in Supplementary Figure D. One can see that the average cLD of the int group is significantly higher than that in the no interaction regions. The corresponding numerical values supporting this figure are listed in Supplementary Table I. The rigorous test to justify the significance statistically will be elaborated in section 3.4. This result indicates that cLD statistic captures the 3D interaction between genes. It is also clearly observed that the cLD decreases along with the increased distance between genes. In addition, the largest difference in cLD between int groups and no-int groups occurs in the first distance unit and the difference in cLD between int groups and no-int groups decreases considerably as the distance increases. This phenomenon implies that the interaction between gene pairs with shorter distances is more likely to result in a higher cLD value.

Then, in order to show that cLD is more appropriate than LD in capturing the 3D interaction between genes, we generated Supplementary Figure E, the results analogue to Supplementary Figure D using the standard LD (r^2). The details of how we conducted the calculations are presented as follows. Again the gene pairs are split into the same 13 distance units. Then for each gene pair within the Hi-C assessed 3D-interaction, we randomly chose 2000 SNV pairs from it to calculate the average LD value. For each selected SNV pair, we calculated its distance and then among the gene pairs without Hi-C interaction we randomly

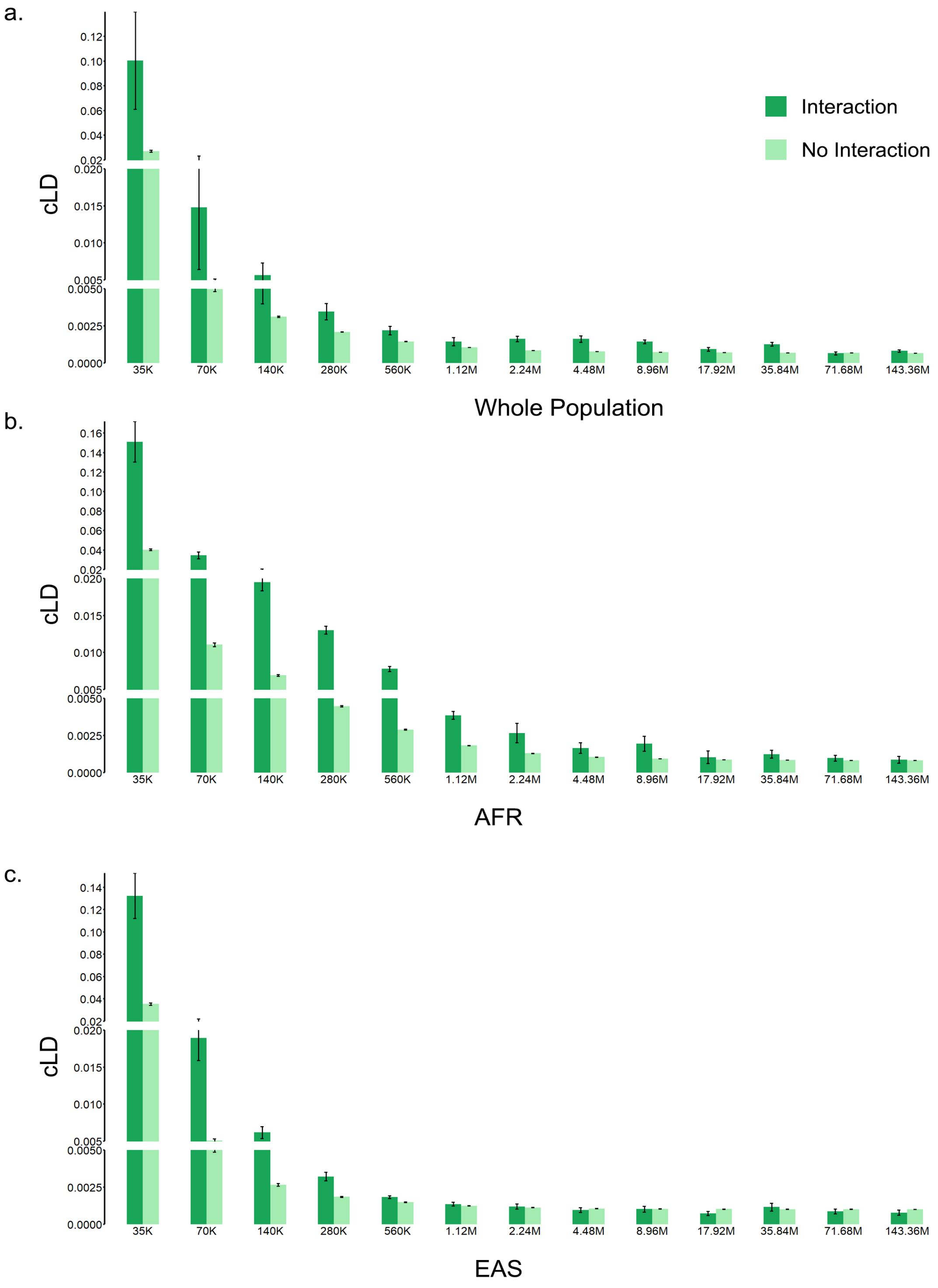

Fig. D: The comparisons of cLD values between the 3D chromatin interaction regions and non-interaction regions among 13 different distance groups in (a) the whole population, (b) AFR, and (c) EAS. The European population, EUR, has been displayed in the main text Figure 3(a)

selected another SNV pair with the same or very similar distance. As a result, we had total 2000 randomly selected SNVs from gene pairs without interaction that are paired up with the 2000 SNVs from gene pairs within 3D-interaction regions. Finally, we grouped the 2000 distance pairs into the 13 distance units and calculated the average LD for in interaction regions and non-interaction regions within each distance unit.

As shown in Supplementary Figure E, the average LD values for the 3D-interaction and non-interaction regions are only slightly different in each of the 13 distance units. Indeed, statistically, the subtle difference is not significant, quantified by our two-sample test in section 3.4.

Therefore, based on the observations from Supplementary Figure D and Supplementary Figure E, as well as their statistical test (to be presented in section 3.4), we concluded that cLD is a more appropriate statistic than LD to capture the 3D interaction between genes.

The 95% quantiles of the cLD in 3D chromatin interaction regions are shown in the Supplementary table I.

From this Table I, we can see the cLD's distribution of the interaction group is higher than the distribution of the no-interaction group. This means our cLD value does distinguish the interaction between genes.

3.3 LD and cLD in databases for gene-gene interactions

In this section, we first gave an introduction to the gene-gene interaction databases used for assessing the enrichment of cLD in interacting gene pairs. Based on these interaction databases, the gene pairs are separated into interaction groups and no-interaction groups. Then we presented the results of mean cLD values in these two groups.

1. Biogrid database

Biogrid [4] is a biomedical interaction repository with data compiled through comprehen-

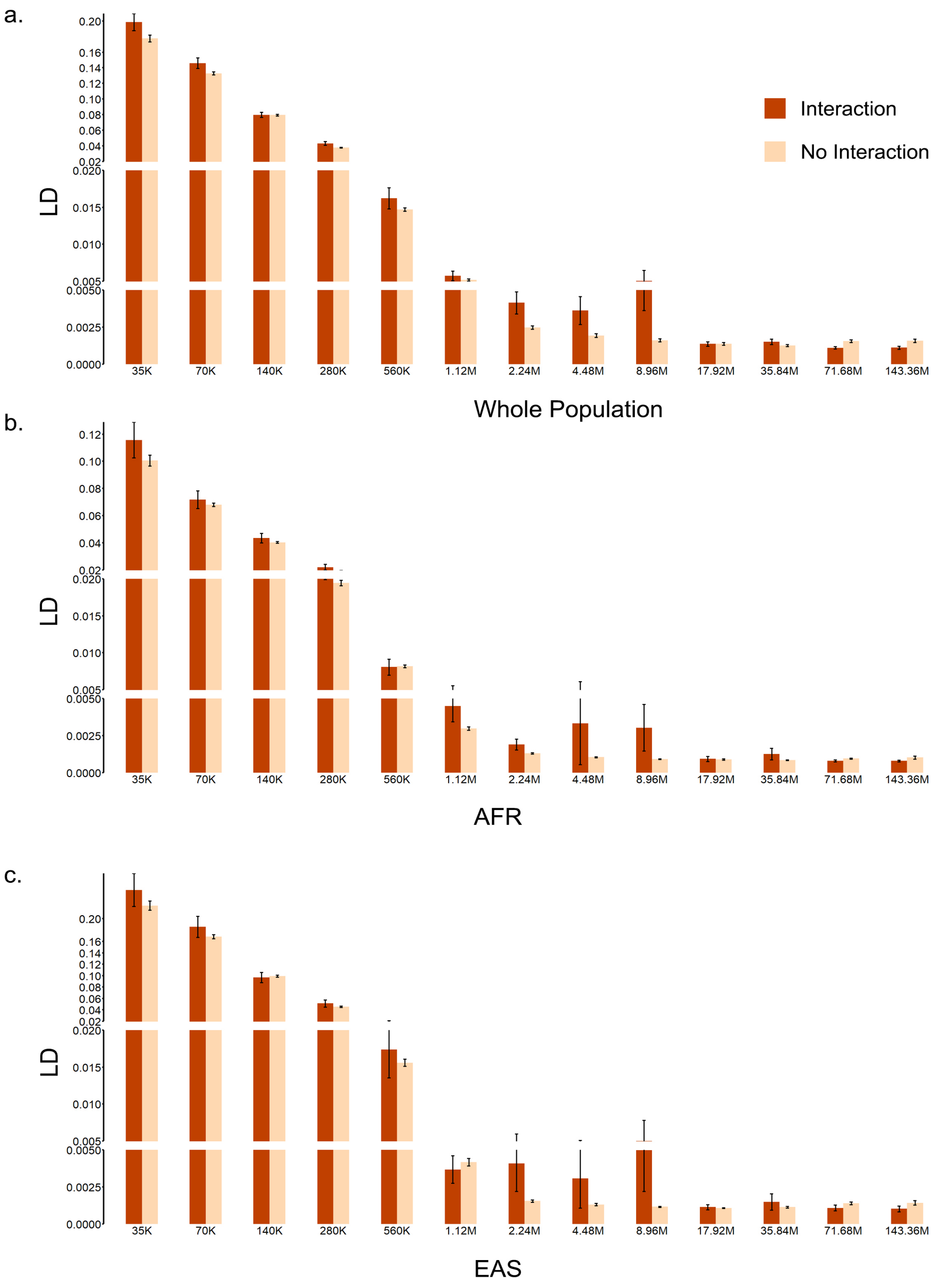

Fig. E: The comparisons of LD values between the 3D chromatin interaction regions and non-interaction regions among 13 different distance groups in (a) the whole population, (b) AFR, and (c) EAS. The European population, EUR, has been displayed in the main text Figure 3(b).

sive curation efforts. Their current index is version 4.4.197 and searches 76,687 publications for 2,045,743 protein and genetic interactions, 29,093 chemical interactions and 1,070,825 post-translational modifications from major model organism species. The data were downloaded from https://thebiogrid.org/.

2. Reactom database

Reactome[5] is a free, open-source, curated and peer-reviewed pathway database. This dataset provides intuitive bioinformatics tools for the visualization, interpretation and analysis of pathway knowledge to support basic research, genome analysis, modelling, systems biology and education. The data were downloaded from https://reactome.org/.

3. MINT database

MINT [6] focuses on experimentally verified protein-protein interactions mined from the scientific literature by expert curators.

Protein interaction databases represent unique tools to store, in a computer-readable form, the protein interaction information disseminated in the scientific literature. Well-organized and easily accessible databases permit the easy retrieval and analysis of large interaction data sets. MINT is a database designed to store data on functional interactions between proteins. Beyond cataloguing binary complexes, MINT was conceived to store other types of functional interactions, including enzymatic modifications of one of the partners. Since the MINT dataset provides interaction between proteins, we used the Uniprot website to convert the protein's UniProtKB to the gene's Ensembl ID. The data were downloaded from https://mint.bio.uniroma2.it/.

4. Intact database

The Intact database [7] is similar to the MINT dataset. Intact provides a freely available, open-source database system and analysis tools for molecular interaction data. All interactions are derived from literature curation or direct user submissions and are freely available. We first transferred the protein-protein interaction to gene-gene interaction and

then conducted further analysis.

We combined the four interaction databases (Biogrid, Reactom, MINT and Intact) into a single data entry. Then we used the cLD statistic calculated before to analyze their distributions in the gene pairs in or not in the interaction databases. The results are displayed in Supplementary Figure F, from which we observed that the cLD of gene pairs with interactions is higher than that of gene pairs without interaction. The statistical significance tests in section 3.4 also quantitatively confirmed this intuition. Thus we concluded that the cLD captures the gene-gene interaction well.

3.4 Two-sample tests assessing significance levels

In this section, we compared the mean cLD (or LD) between the two categories under comparison, i.e., the interaction or non-interaction regions (in terms of either 3D interaction or gene-gene interactions) in a statistical rigorous way. More specifically, we designed the Mantel-Haenszel test and Fisher's exact test to assess the p-values to quantify the significant level.

The null hypothesis of the tests is that the distributions (of cLD, for example) are the same in the two groups (of gene pairs in interaction or non-interaction regions); and the alternative hypothesis is that the distributions are not the same. To distinguish these two hypotheses, we designed a 2 x 13 contingency table, in which 2 columns are for two distributions and 13 rows are for 13 distance groups. Each cell of the contingency table will be filled in the following way:

First we create a "standard" by calculating the median (i.e., the 0.5 quantile) value in the whole population (e.g., EUR or AFR), denoted by $q_{0.5}$. Then we define the "success rate" for a given distance group and a given distribution (interaction or non-interaction) as the proportion of statistics (such as cLD or LD) that are larger than the standard value of $q_{0.5}$. Then we put these 13 x 2 success rates into the contingency table.

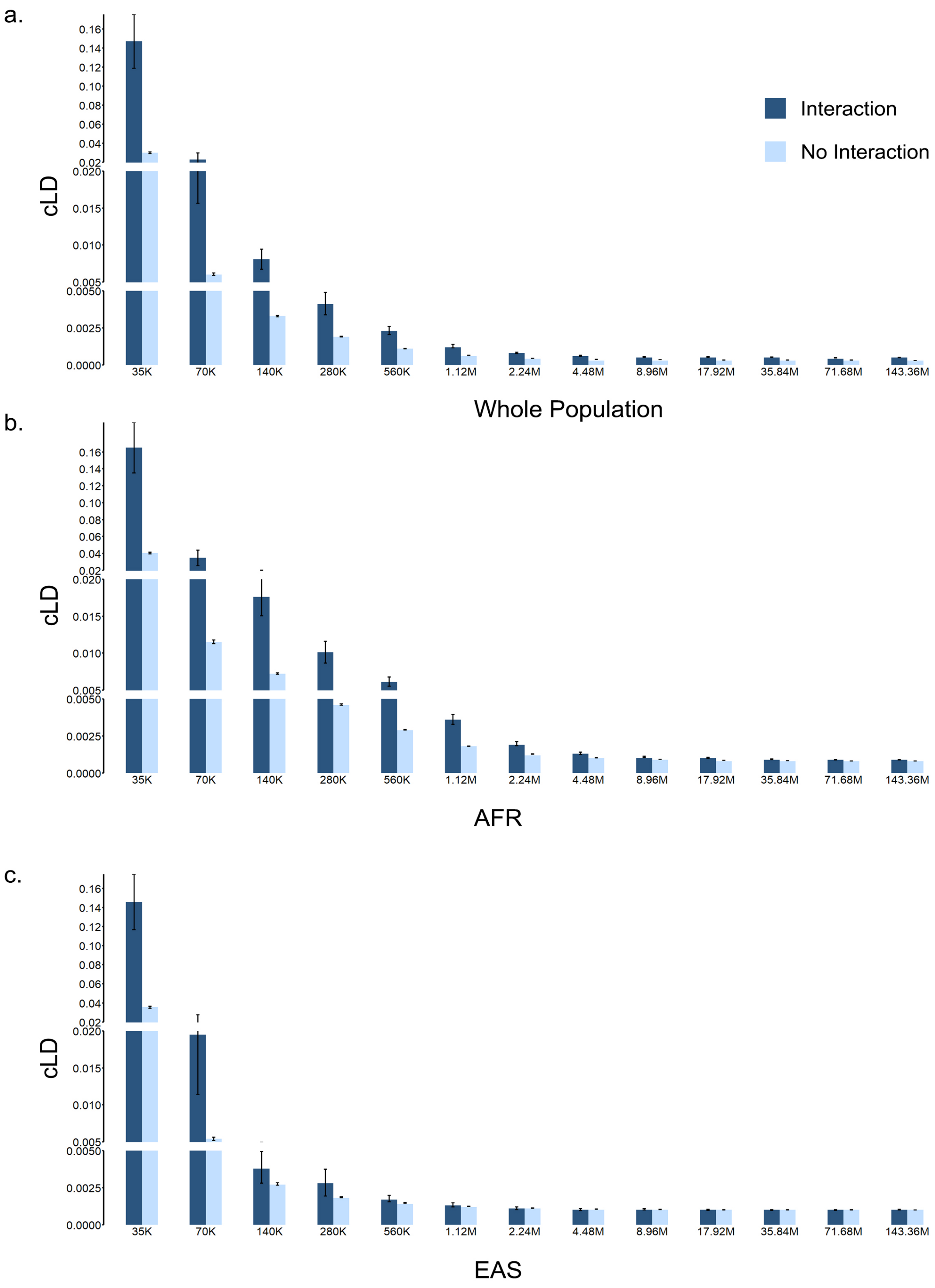

Fig. F: The comparisons of cLD values between the gene-gene interaction regions and regions without interactions among 13 different distance groups in (a) the whole population, (b) AFR, and (c) EAS. The European population, EUR, has been displayed in the main text Figure 4.

If we view the data collected from 13 different distance groups as independent experiments, then the standard Mantel-Haenszel test can be used to test the significance level. Meantime, Fisher's Exact test can also be utilized to test whether the two columns are from the same distribution.

By applying the above Mantel-Haenszel test and Fisher's Exact test to the cLD and LD distributions, we assessed whether this visible or subtle differences displayed in the figures in section 3.2 and section 3.3 are statistically significant.

3.4.1 Two-sample Tests of cLD & LD in 3D interacting regions

We conducted the Mantel-Haenszel test and Fisher's exact test for LD and cLD in the Hi-C assessed 3D interaction regions. Since the results for different populations are quite similar, we only presented those for EUR in Table J as an illustration. The results for AFR, EAS and the whole population are listed in Supplementary Table R (enclosed the attached Excel file).

Based the protocol described in the previous subsection, we formed the data for the test in Supplementary Table J for EUR population. The last two columns (Int-ration and Non-int-ratio) are the contingency table ready for the two-sample test.

The Fisher's Exact test statistic is 29.84 and the Mantel-Haenszel test statistic is 12.99. The p-values for both tests are less than 1E-50 (based on the corresponding R libraries) and thus we rejected the null hypothesis based on either test. The Fisher's Exact and the Mantel-Haenszel test statistics are 51.34 and 28.52 respectively for EAS population, and 87.80 and 34.81 for AFR population. The p-values of these test statistics are also less than 1E-50. Therefore we concluded that, the cLD values in the 3D interaction regions are significantly higher than that of the non-interaction regions.

Similarly, for LD, again using EUR as the representative example, the number of successes, number of failures and success/failure ratios for interaction and non-interaction groups are presented in Supplementary Table K.

Based on Supplementary Table K for the EUR population, the Fisher's Exact test statistic is -45.65 and the Mantel-Haenszel test statistic is -45.68. The p-values for both tests are very close to 1 and thus we could not reject the null hypothesis based on either test. The Fisher's Exact and the Mantel-Haenszel test statistics are -21.34 and -21.35 respectively for EAS population, and -20.74 and -20.75 for AFR population. The p-values of these test statistics are also very close to 1. Therefore we concluded that, the LD in the 3D-interaction regions are not higher than that of the non-interaction group. In other words, the high-LD gene pairs are not overlapped with the gene pairs within the 3D interaction regions, hence the standard LD could not capture the 3D interactions between gene pairs.

3.4.2 Two-sample tests of cLD in gene-gene interaction databases

Analogue to the case of 3D-interactions, we conducted the same two-sample tests to the categorization based on gene-gene interactions. We combined all the reported interactions by the four datasets Biogrid, Reactom, MINT and Intact to form the interaction category and the compliment as the non-interaction category. The number of successes, number of failures and the success/failure ratios for interaction and non-interaction groups for EUR, AFR and EAS are presented in Supplementary Table Q (enclosed the attached Excel file).

Based on the contingency table for the EUR population, the Fisher's Exact test statistic is 60.09 and the Mantel-Haenszel test statistic is 40.59. The p-values for both tests are less than 1E-50 and thus we rejected the null hypothesis based on either test. Based on the table for AFR population, the Fisher's Exact test statistic is 58.73 and the Mantel-Haenszel test statistic is 39.72. The p-values for both tests are again less than 1E-50 and thus we rejected the null hypothesis based on either test. Based on the table for EAS population, the Fisher's Exact test statistic is 87.80 and the Mantel-Haenszel test statistic is 34.81. The p-values for both tests are less than 1E-50 and thus we rejected the null hypothesis based on either test. Taking together, the above results proved that cLD captures the gene-gene interaction well.

3.5 Enrichment of high-cLD pairs in 3D Interaction regions

In this section we provide details of the analysis of the enrichment of high-cLD pairs as a function of the extreme value of cLD, which is presented in the Main Text Figure 3c,d,e.

First, we set up multiple cLD cutoffs to distinguish different levels of extremely high values, i.e., 0.05, 0.1, 0.2, 0.3, and 0.4. Then, for the interval between every two consecutive cutoffs, we count gene pairs with their values falling in the interval and split them into regions covered by 3D interactions or not, respectively. As the number of gene pairs is huge, we only considered gene pairs on chromosome 1 and chromosome 2, which in contains 15,102,208 and 6,053,460 gene pairs, respectively. Amount these gene pairs, 1,686 and with 1,357 are within the 3D regions. Next, we calculated the percentage of gene pairs within 3D-interaction regions with the cLD values falling in the internal (P_1) as well as the percentage of such high-cLD gene pairs in the whole chromosome (P_2). The ratio between the above two percentages is plotted as the "Improved ratio" for this particular interval defined by two cLD-value cutoffs. The specific math formula are:

$$P_1 = \frac{Number\ of\ high\ cLD\ gene\ pairs\ within\ 3D\ regions}{Total\ number\ of\ gene\ pairs\ within\ 3D\ regions}$$

$$P_2 = \frac{Number\ of\ high\ cLD\ gene\ pairs\ in\ the\ chromosome}{Total\ number\ of\ gene\ pairs\ in\ the\ chromosome}$$

$$Improved\ ratio = \frac{P_1}{P_2}$$

Evidently, the improved ratios at different cLD cutoffs, across different populations (EUR, EAS and AFR), are large, and enjoy an increasing trend as the cLD cutoff increases (Main text Figure 3c,d,e; Supplementary Tables S).

Table I: The quantiles of cLD values in the 3D chromatin interaction regions and non-interaction regions among 13 different distance groups in three populations.

**EUR**						
Distance groups	Int 0.025	Int 0.5	Int 0.975	No Int 0.025	No Int 0.5	No Int 0.975
35K	2e-5	0.0150	0.8712	2e-6	0.0017	0.4499
70K	1e-5	0.0043	0.2516	3e-6	0.0007	0.0514
140K	5e-6	0.0030	0.0543	3e-6	0.0005	0.0280
280K	5e-6	0.0021	0.0276	2e-6	0.0004	0.0200
560K	3e-6	0.0013	0.0182	2e-6	0.0003	0.0143
1.12M	3e-6	0.0007	0.0136	2e-6	0.0003	0.0105
2.24M	1e-6	0.0005	0.0081	9e-7	0.0003	0.0080
4.48M	1e-6	0.0005	0.0060	7e-7	0.0002	0.0063
8.96M	2e-6	0.0004	0.0126	6e-7	0.0002	0.0060
17.92M	3e-6	0.0004	0.0051	5e-7	0.0002	0.0061
35.84M	1e-6	0.0004	0.0078	5e-7	0.0002	0.0059
71.88M	1e-6	0.0004	0.0067	5e-7	0.0002	0.0060
143.36M	1e-6	0.0006	0.0064	6e-7	0.0002	0.0057
**AFR**						
Distance groups	Int 0.025	Int 0.5	Int 0.975	No Int 0.025	No Int 0.5	No Int 0.975
35K	5e-5	0.0238	0.9068	2e-6	0.0039	0.4356
70K	7e-5	0.0119	0.3047	2e-6	0.0020	0.0657
140K	3e-5	0.0083	0.0783	2e-6	0.0012	0.0454
280K	7e-6	0.0049	0.0461	2e-6	0.0007	0.0313
560K	1e-5	0.0034	0.0300	2e-6	0.0005	0.0202
1.12M	3e-6	0.0016	0.0192	2e-6	0.0004	0.0120
2.24M	1e-6	0.0008	0.0103	1e-6	0.0003	0.0080
4.48M	1e-6	0.0005	0.0070	1e-6	0.0003	0.0061
8.96M	1e-6	0.0004	0.0056	9e-7	0.0003	0.0054
17.92M	1e-6	0.0004	0.0054	8e-7	0.0002	0.0049
35.84M	8e-7	0.0004	0.0048	9e-7	0.0002	0.0047
71.88M	8e-7	0.0004	0.0046	8e-7	0.0002	0.0046
143.36M	7e-7	0.0004	0.0044	6e-7	0.0002	0.0045
**EAS**						
Distance groups	Int 0.025	Int 0.5	Int 0.975	No Int 0.025	No Int 0.5	No Int 0.975
35K	1e-5	0.0106	0.8201	2e-6	0.0007	0.4352
70K	7e-6	0.0017	0.2281	2e-6	0.0004	0.0324
140K	4e-6	0.0013	0.0402	1e-6	0.0004	0.0162
280K	3e-6	0.0009	0.0166	1e-6	0.0003	0.0117
560K	2e-6	0.0007	0.0098	1e-6	0.0002	0.0092
1.12M	2e-6	0.0005	0.0074	5e-7	0.0003	0.0074
2.24M	1e-6	0.0005	0.0057	4e-7	0.0003	0.0065
4.48M	9e-7	0.0003	0.0056	4e-7	0.0003	0.0060
8.96M	1e-6	0.0004	0.0053	3e-7	0.0002	0.0059
17.92M	1e-6	0.0003	0.0046	4e-7	0.0002	0.0058
35.84M	2e-6	0.0004	0.0047	3e-7	0.0002	0.0057
71.88M	1e-6	0.0004	0.0043	3e-7	0.0002	0.0057
143.36M	1e-7	0.0004	0.0036	3e-7	0.0002	0.0057

Table J: EUR counts for Mantel-Haenszel test (3D-interaction, cLD)

	Int-succ	Int-fail	Non-int-succ	Non-int-fail	Int-ratio	Non-int-ratio
35K	75	7	47642	21004	.91	.69
70K	209	12	45516	19804	.95	.70
140K	318	44	82967	38725	.88	.68
280K	379	51	148833	78943	.88	.65
560K	337	68	260934	163980	.83	.61
1.12M	100	38	451231	335797	.72	.57
2.24M	51	31	779888	661248	.62	.54
4.48M	43	20	1346386	1238435	.68	.52
8.96M	41	30	2389097	2281671	.57	.51
17.92M	72	46	4126180	4073582	.61	.50
35.84M	122	65	6792988	6878739	.65	.49
71.88M	154	54	9759922	10001248	.68	.49
143.36M	158	72	11206696	11646643	.53	.49
Overall	2059	538	37438280	37439819	.79	.50

Table K: EUR counts for Mantel-Haenszel test (Hi-C, LD)

	Int-succ	Int-fail	Non-int-succ	Noint-fail	Int-ratio	Non-int-ratio
35K	764	43	807	0	.94	1.
70K	1786	365	2151	0	.83	1.
140K	2854	1480	4323	11	.65	1.
280K	2137	3024	4515	646	.41	.87
560K	720	3506	1420	2808	.17	.34
1.12M	123	2019	9	2133	.06	0
2.24M	41	956	0	999	.02	0
4.48M	15	556	0	572	.02	0
8.96M	26	403	0	430	.06	01
17.92M	0	234	0	234	0	0
35.84M	0	260	0	261	0	0
71.88M	0	258	0	117	0	0
143.36M	0	117	0	117	0	0
Overall	8466	13221	13225	8462	.39	.61

Chapter 4

Protein interactions and docking

Despite the large number of gene-gene interactions reported by the four databases used in section 3.3, many of the gene pairs with high cLD are still not found in these databases, such as between 3BCZ and 4RIQ (encoded for by genes MEMO1 and DPY30, respectively) with a cLD of 0.86. This leaves room for discovering novel gene-gene interactions indicated by high cLD values. In this chapter, we presented our characterization of the novel interactions by computational prediction of protein docking. This enables us to investigate the nature of the relationship between the two cLD-identified genes through the receptor-ligand interactions of their translated proteins.

4.1 Pipeline for protein-protein docking analysis

The interacting protein-protein candidates were selected by identifying the top gene pairs with cLD values between the ranges of 0.33 to 0.86. Subsequently, the proteins' tertiary structures were obtained from the RCSB Protein Data Bank (PDB) [8]. The structures were validated based on their resolution being less than 3Å, having almost zero Ramachandran outliers, and the macro-molecules parent organism being Homo sapiens. The structures were visualized and prepared for docking using PyMOL [9].

We used Huang Lab's software HDOCKlite for protein-protein docking. HDOCKlite is

a user downloadable software-based version of their more famous, CASP-CAPRI winning HDOCK server. HDOCK's docking protocol uses a hybrid protein docking technique that incorporates both ab-initio and homology-based principles [10, 11]. We selected HDOCKlite as its a time-tested and validated user-friendly tool, and it can run on High-Performance Computing (HPC) clusters. Additionally, it had very short processing times enabling us to conduct multiple analyses efficiently. The docking analysis was conducted using HDOCK-lite's default parameters.

Once the docking analysis was conducted between the candidate pairs, post-processing of the protein-protein complexes was conducted. First, it was ensured that the formed complexes had negative binding energies. Based on the laws of thermodynamics, the more negative the value of the binding energy, the more stable the protein substrate complex formed between the two proteins [10]. Afterwards, the 3D complex was examined in PyMOL and the interacting surfaces of the two macro-molecules were investigated using DIMPLOT from LigPlot+ [12]. DIMPLOT provides a 2D representation of the interacting surfaces at an atomic level complete with the chemical bonds responsible for the establishment of the protein-protein complex.

4.2 Protein-protein docking results

We conducted protein docking analysis for the 19 pairs of genes with large cLD values (top 0.01% among all gene pairs) with cMAF $>$ 0.05 and existing IDs in PDB, however, not reported in any databases Supplementary Table L. As examples, we selected five pairs of protein-protein docking results for presentation. The detailed list of the candidate proteins and their cLD values as well as binding energy are listed below in Supplementary Table M. The graphical overviews of the formed complexes are depicted in this section as Supplementary Figures G, H, I and J except that of the 3BCZ and 4RIQ complex which is present in the Main Text Figure 5.

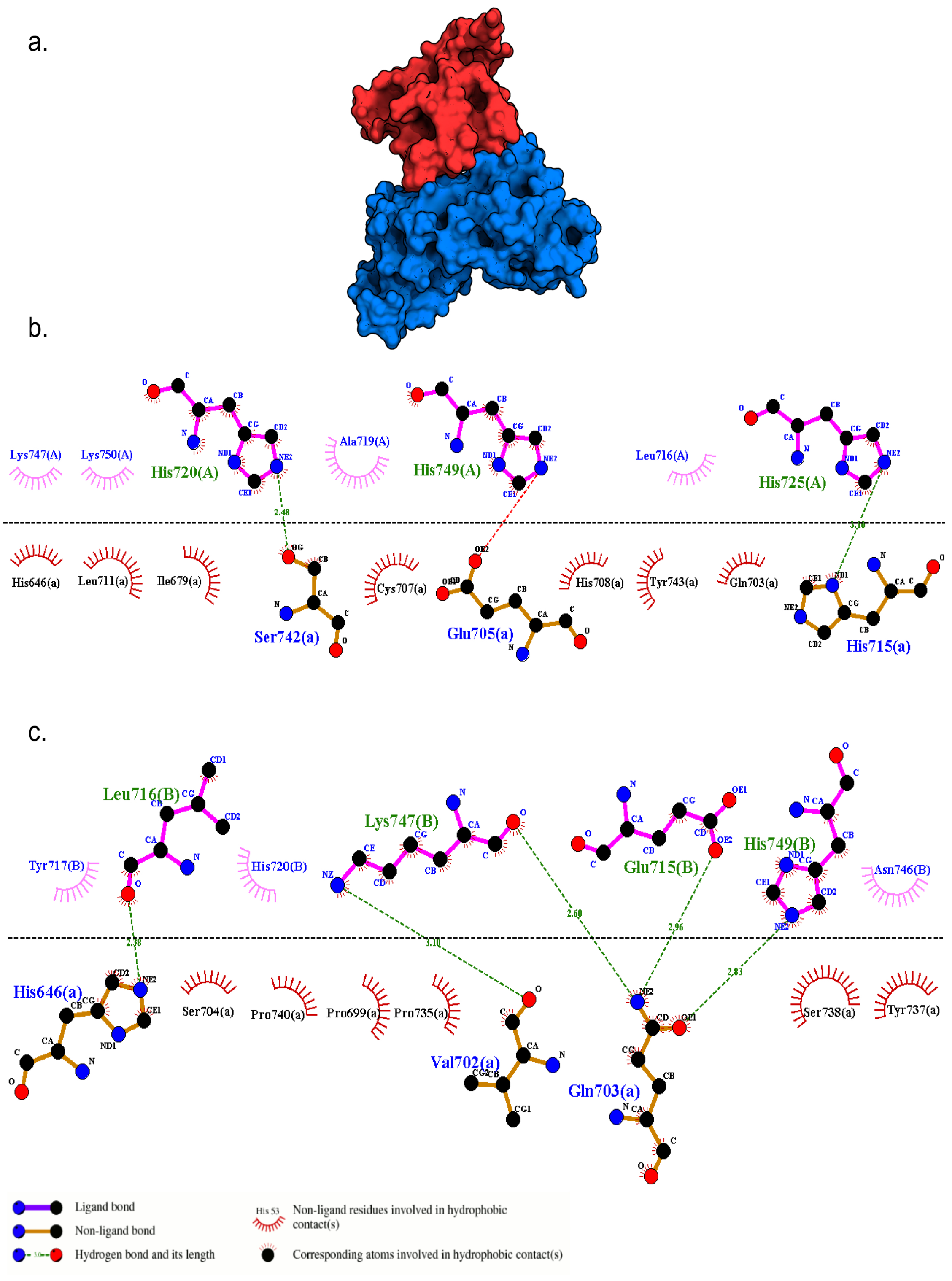

Fig. G: Protein docking interaction between 1FYV and 4OM revealed by cLD (0.69) with a binding affinity of -266.77 kJ/mol. a) Structure of 1FYV (red) and 4OM (blue) protein-protein complex. b-c) 2D representation of closest interacting residues around the protein-protein interaction interfaces, including hydrogen bonds (green dotted line) and hydrophobic interactions (read and rose semi-circle with spikes). Residues for the 1FYV are depicted by uppercase letters (A, B) and for the 4OM are depicted by lower lowercase letter (a).

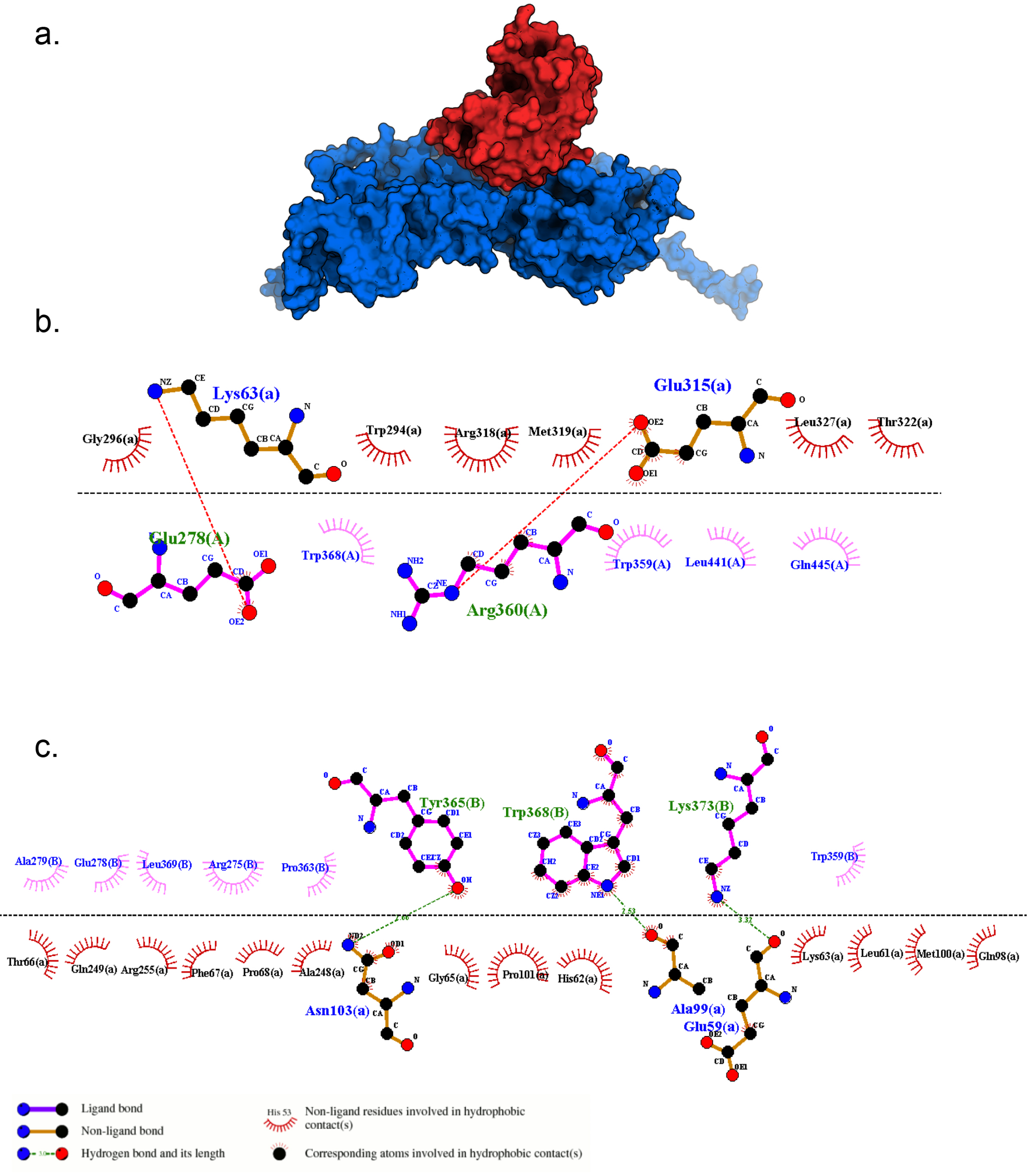

Fig. H: Protein docking interaction between 3S5N and 4HND revealed by cLD (0.52) with a binding affinity of -302.64 kJ/mol. a) Structure of 3S5N (red) and 4HND (blue) protein-protein complex. b-c) 2D representation of closest interacting residues around the protein-protein interaction interfaces, including hydrogen bonds (green dotted line) and hydrophobic interactions (read and rose semi-circle with spikes). Residues for the 3S5N are depicted by uppercase letter (A) and for the 4HND are depicted by lowercase letters (a, b).

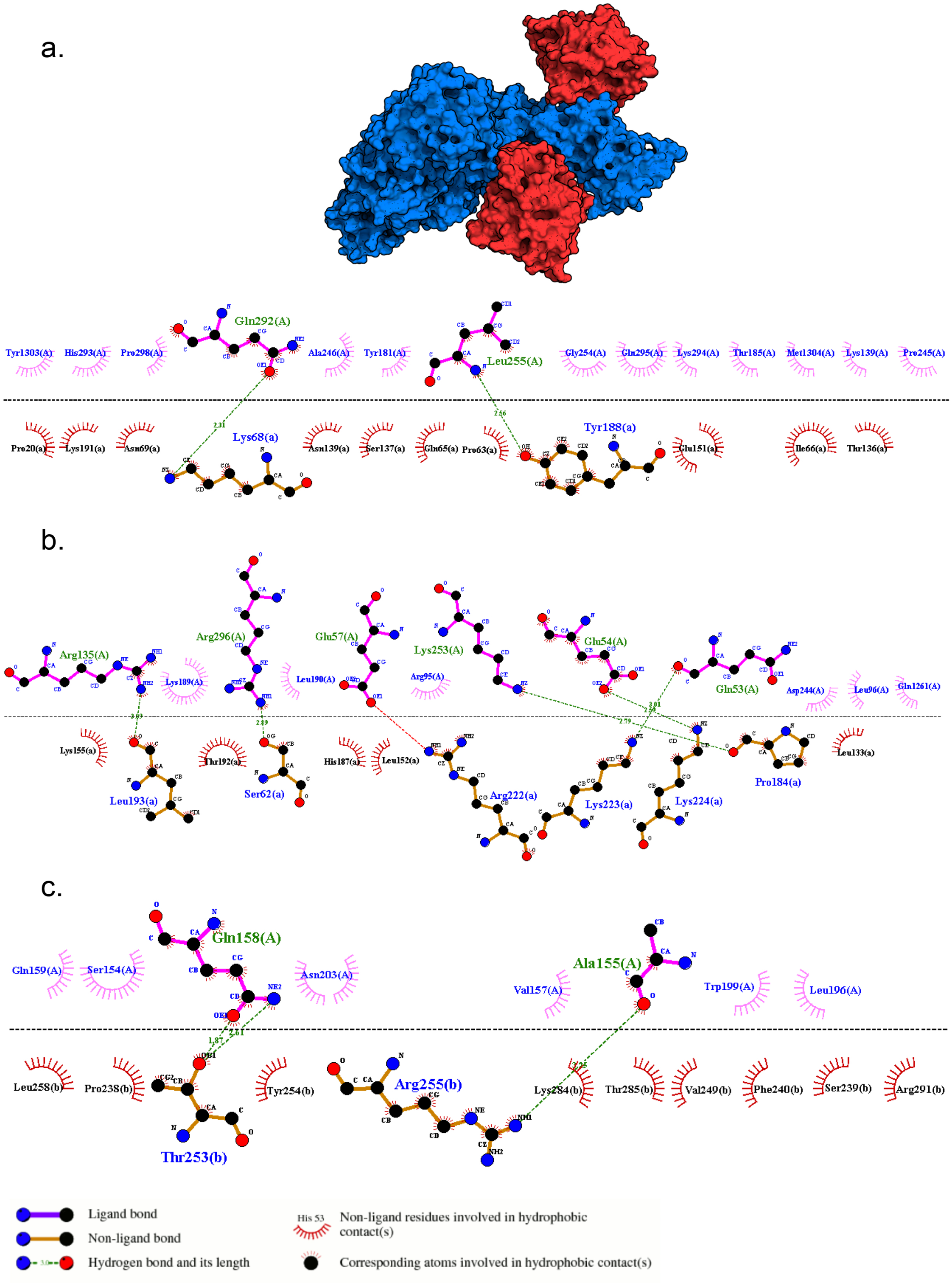

Fig. I: Protein docking interaction between 1WVA and 6HO2 revealed by cLD (0.40) with a binding affinity of -263.19 kJ/mol. a) Structure of 1WVA (red) and 6HO2 (blue) protein-protein complex. b-c) 2D representation of closest interacting residues around the protein-protein interaction interfaces, including hydrogen bonds (green dotted line) and hydrophobic interactions (read and rose semi-circle with spikes). Residues for the 1WVA are depicted by uppercase letter (I) and for the 6HO2 are depicted in lowercase letter (a).

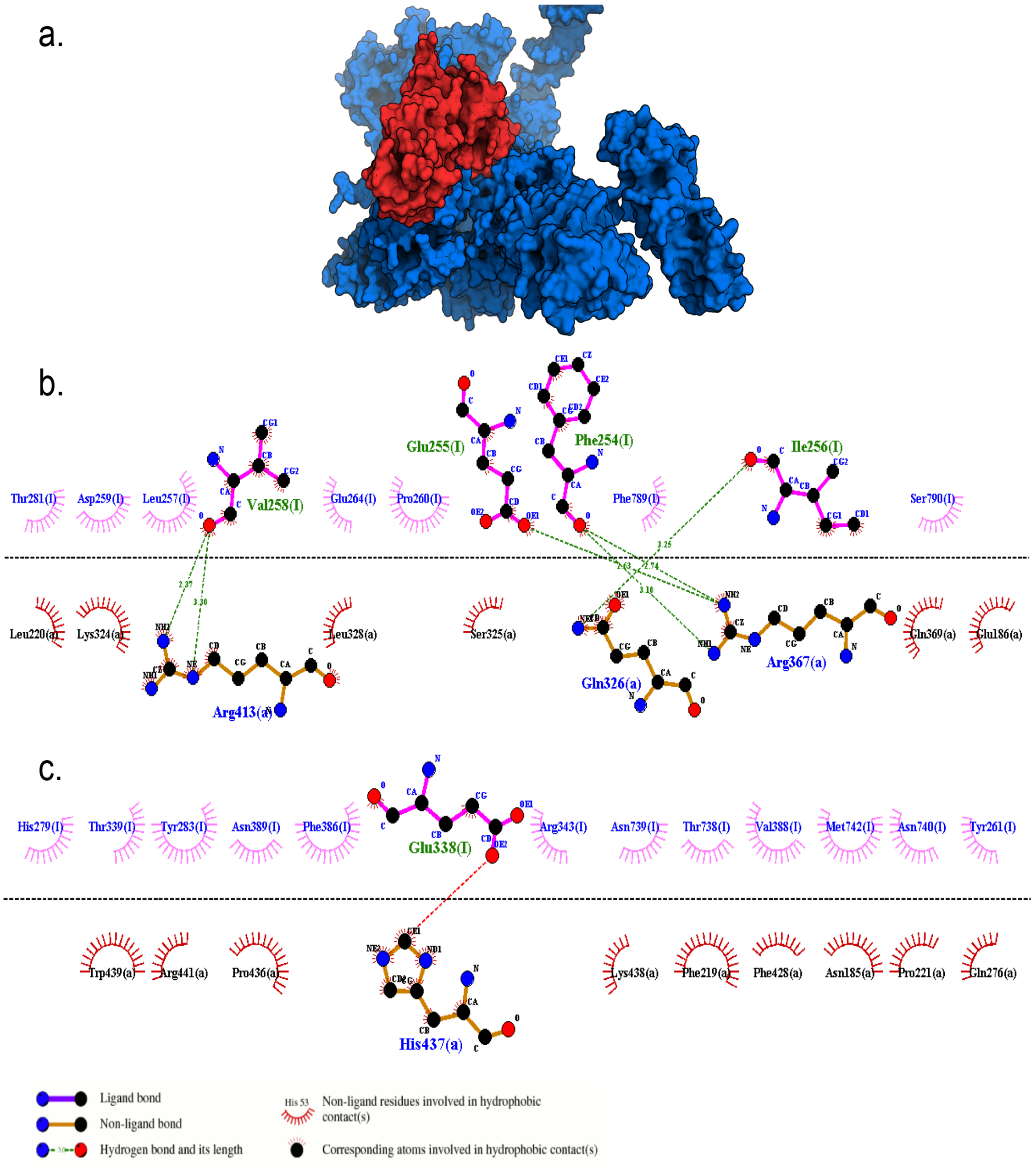

Fig. J: Protein docking interaction between 3L81 and 5FUR revealed by cLD (0.33) with a binding affinity of -277.36 kJ/mol. a) Structure of 3L81 (red) and 5FUR (blue) protein-protein complex. b-c) 2D representation of closest interacting residues around the protein-protein interaction interfaces, including hydrogen bonds (green dotted line) and hydrophobic interactions (read and rose semi-circle with spikes). Residues for the 3L81 are depicted by uppercase letters (A, B) and for the 5FUR are depicted in the lowercase letter (a).

Table L: List of 19 gene pairs(not reported in any databases) with large cLD values with cMAF > 0.05 and existing IDs in PDB.

Ensembl ID 1	PDB ID 1	Ensembl ID 2	PDB ID 2
ENSG00000162959	3BCZ	ENSG00000162961	4RIQ
ENSG00000114779	1IMJ	ENSG00000243989	1Q7L
ENSG00000168827	6VLZ	ENSG00000079257	2BO9
ENSG00000174125	1FYV	ENSG00000174130	4OM7
ENSG00000131187	6SZW	ENSG00000198055	2ACX
ENSG00000166278	2I6Q	ENSG00000243649	1DLE
ENSG00000240065	6AVO	ENSG00000168394	1JJ7
ENSG00000231389	3LQZ	ENSG00000223865	3LQZ
ENSG00000124596	2EEE	ENSG00000001167	4AWL
ENSG00000118520	1WVA	ENSG00000112282	6H02
ENSG00000241685	6YW7	ENSG00000130429	6UHC
ENSG00000221838	3L81	ENSG00000106290	5FUR
ENSG00000087077	1X61	ENSG00000087085	1B41
ENSG00000197448	1YZX	ENSG00000106144	6GKF
ENSG00000133742	1AZM	ENSG00000164879	1Z93
ENSG00000042832	6SCJ	ENSG00000155926	2CUD
ENSG00000122705	6E5N	ENSG00000159921	2YHW
ENSG00000095380	1WVO	ENSG00000106785	6JBM
ENSG00000241935	3S5N	ENSG00000155252	4HND

Table M: List of candidate proteins with their respective cLD values and binding affinities. All candidates formed stable protein-protein complexes with negative binding energies.

Ensembl ID	Gene Name	PDB ID	Ensembl ID	Gene Name	PDB ID
ENSG00000162959	*MEMO1*	3BCZ	ENSG00000162961	*DPY30*	4RIQ
ENSG00000174125	*TLR1*	1FYV	ENSG00000174130	*TRL6*	4OM7
ENSG00000241935	*HOGA*1	3S5N	ENSG00000155252	*PI4K2A*	4HND
ENSG00000118520	*ARG1*	1WVA	ENSG00000112282	*MED23*	6H02
ENSG0000221838	*AP4M1*	3L81	ENSG00000106290	*TAF6*	5FUR

Chapter 5

Association mapping using cLD and annotations

In this chapter, we tested whether cLD serves as a valid tool to discover genes whose interactions may play critical role in diseases. More specifically, we conducted analysis to assess whether gene pairs with highly differential cLD(ΔcLD) values distinguishing cases and controls are enriched in functionally relevant databases as well as sensible gene ontology (GO) and pathways (reported by the KEGG repository).

5.1 Data source and quality control

We calculated cLD on Autism Spectrum Disorder (ASD) whole exon sequencing dataset [18], which is downloaded from dbGaP web portal (http://www.ncbi.nlm.nih.gov, ID: phs000298.v4.p3). The dataset is quality controlled using PLINK [19]. The QC steps include excluding markers with missing rate > 0.1, high deviations from Hardy-Weinberg Equilibrium > 10-6, and removing samples with missing rate > 0.1.

5.2 Calculating cLD in case-control datasets

We conducted the analysis composed of the following 5 steps:

Step 1: For the target whole exome sequencing dataset, we only retained rare variants with MAF < 0.005

Step 2: We then located gene region for all human genes, and the corresponding gene information is downloaded from GENCODE (https://www.gencodegenes.org/human/). The gene region is ranging from location start to location end.

Step 3: For the target dataset, if there is no rare variant within the gene region, this gene will be excluded, otherwise, we further split the entire dataset into case and control groups. We then calculated the cMAF within each gene region for cases and controls separately.

Step 4: We retained genes with cMAF > 0.05 in both case and control groups.

Step 5: Lastly, we calculated cLD within the gene regions for each gene pair. This calculation was also done for case and control separately.

5.3 Enrichment of cLD-differential genes in functional databases

We calculated the absolute differences between the cLD values in cases and controls for every pairs of genes. This difference is the contrast between cases and controls in terms of cLD indicated interactions. We then ranked them from largest to smallest. From the ranked list, we selected the top 200, 500, 1,000, 1,500, or 2,000 gene pairs for functional annotations. For each set of high ΔcLD pairs, we form a gene list containing all genes included by at least a pair.

The model for the functional annotation is to check the enrichment of cLD identified genes in known databases. We referred to two databases: one is from Simons Foundation Autism Research Initiative (SFARI) [20] and the other one is from DisGeNet [21]. The SFARI database is a well-established repository for existing ASD genes [20] and its data can be directly downloaded. For the DisGeNet database, we used "Autism" as the keyword and searched for all Autism-associated genes. We checked these two databases separately. For a given protocol, we defined the "success" as the number of identified genes reported in the corresponding database, and the "success rate" as the ratio of these validated genes against all of the selected genes.

To test whether the enrichment is significant. We calculated the p-values under hypergeometric distribution. The density of the hypergeometric distribution with parameters m, n, k, and $N = m + n$, is given by the formula below:

$$P(x) = \frac{\binom{m}{x}\binom{n}{k-x}}{\binom{N}{k}}$$

m: The number gene is the database. The database is downloaded from either SFARI or DisGeNet.

n: The total number of genes in the population ($= N$) minus the number of genes in the database ($= m$). Here, N =20,000, as we considered roughly 20,000 human genes in the population.

k: The number of genes from selected top (200, 500, ..., 2000) gene pairs.

q: The number of selected genes being validated in the database.

We calculated the distribution function using `"phyper"`, provided in R. The p-value is calculated as `1- phyper(q, m, n, k, lower.tail = TRUE, log.p = FALSE)`. If the p-value < 0.05, it is considered as a significant enrichment.

By applying the above hypergeometric test to the previously formed ΔcLD gene lists and the DisGeNet and SFARI databases, we could quantify the enrichment led by different sets

of high ΔcLD genes. It is observed that, as we included more top gene pairs (from 200 to 2,000), the number of success is increasing in both DisGeNet and SFARI databases. The success rate is ranging from 9.80% to 10.34% for the DisGeNet database, the success rate increased a little bit as we included more genes. For the SFARI database, the success rate is ranging from 6.72% to 8.84%, which is a bit lower than the success rate in the DisGeNet database. This might be due to the reason that firmly verified ASD genes in the SFARI database are limited (Supplementary Table N). Looking at the enrichment p-value in both databases, it is evident that the p-values are significant for all different selection of gene pairs (from top 200 to top 2,000), and the p-value is decreasing (i.e., more significant) dramatically as we include more gene pairs (Main Text Figure 6a, 6b and Supplementary Table N). This indicated that high ΔcLD genes between the case and control may be highly associated with diseases.

From the top 10 gene pairs with the highest cLD values, we identified 20 unique genes. 14 out of these 20 genes (70%) have been reported to be associated with ASD, including *DENND4A*, *EFCAB5*, *ABI2*, *RAPH1*, *MSTO1*, *DAP3*, *ARL13B*, *PRB2*, *PRB1*, *ZNF276*, *FANCA*, *ADAM7*, *SLC26A1* and *TUBB8* (Supplementary Table N). Among the rest of six genes, some genes also showed indirect associations with ASD. For example, mutations of *RAB11A* were identified in patients with developmental diseases or/and epilepsy [13]. Gene *IDUA* participates in the degradation of glycosaminoglycans (GAGs) within the lysosome [14], and degradation of glycosaminoglycans has been suggested to be occurred in ASD patients [15]. Long noncoding RNA has been widely reported to be associated with ASD [16, 17], therefore *RP11-1148O4.2* , *RP11-624C23.1* and *DHRS4-AS1* may consider as potential candidate genes for ASD.

Table N: From the top 10 gene pairs with the highest cLD values, we identified 20 unique genes. 14 out of these 20 genes (70%) have been reported to be associated with ASD, including *DENND4A, EFCAB5, ABI2, RAPH1, MSTO1, DAP3, ARL13B, PRB2, PRB1, ZNF276, FANCA, ADAM7, SLC26A1* and *TUBB8*.

Gene 1	Gene 2	cLD	Reference 1	Reference 2
RAB11A	DENND4A	0.223		PMID: 30559488
EFCAB5	RP11-1148O4.2	0.200	PMID: 26189338<br>PMID: 22542183<br>PMID: 24267886	
ABI2	RAPH1	0.188	PMID: 30610205<br>PMID: 15542242<br>PMID: 33157009	PMID: 26731442
MSTO1	DAP3	0.170	PMID: 31463572<br>PMID: 28544275	PMID: 28392909<br>PMID: 25363768
ARL13B	DHFRL1	0.165	PMID: 23153492	
PRB2	PRB1	0.159	PMID: 31540669<br>PMID: 27708715	PMID: 31540669<br>PMID: 27708715
ZNF276	FANCA	0.157	PMID: 31838722<br>PMID: 33431980	PMID: 25531569
ADAM7	RP11-624C23.1	0.146	PMID: 33374371	
IDUA	SLC26A1	0.141		PMID: 33374967<br>PMID: 26399424
TUBB8	DHRS4-AS1	0.134	PMID: 22169095	

5.4 Enrichment of cLD-differential genes in GO and KEGG

As another independent line of analysis in parallel to the DisGeNet and SFARI databases, we conducted gene ontology, or GO, enrichment [22] and KEGG pathway analysis [23]. The GO enrichment is carried out from the aspect of biological process (BP) [22]. KEGG is used to systematically analyze gene functions, which links gene information with high-level functional information [24]. The GO enrichment and KEGG pathway analyses were conducted using the 'clusterProfiler' R package [25]. The enriched GO terms and KEGG pathways with a p-value < 0.05 were considered disease-related biologic processes or signalling pathways.

We included all selected genes within the top 2000 gene pairs with high ΔcLD for the GO enrichment and KEGG pathway analysis. We showed that genes with high ΔcLD are

enriched in sensible pathways and biological processes that are related to ASD (Main Text Figure 6c, 6d). Here are examples supported by literature:

Glutamatergic synapse was the leading pathway identified in KEGG analysis (Main Text Figure 6c). Glutamate is the major excitatory neurotransmitter in the central nervous system and its dysfunction has been central to neurotransmitters that are involved in autism[26]. A fruitful of GWAS studies have reported the associations between genes involved in glutamatergic neurotransmission and ASD [27–29]. Moreover, glutamatergic synapses play a key role in neuronal functions, including synaptic transmission, neuronal migration, excitability, plasticity, long-term potentiation, and long-term depression [30]. Many genes, including SHANK families [31–33], NLGN and NRXN families [34] related to the glutamatergic synapses have been found to be associated with ASD [35].

Followed by the Glutamatergic synapse, the second most enriched pathway is the focal adhesion. Many neuronal cell adhesion molecules help form the structure of the neuronal network during development and are involved in cognitive functions and memory [36]. For ASD, a body of studies have also demonstrated that alterations in synaptic genes including those encoding cell adhesion molecules, e.g., cadherins, NCAM, and neurexin superfamily, can affect the structural connectivity of neurons, thus play important roles in the pathogenesis of ASD [37–39].

From the most significantly enriched GO terms, we observed extracellular matrix organization, extracellular structure organization and cell-substrate adhesion are associated with the structural integrity of the neuronal network. Other biological processes, including dendrite development, dendrite morphogenesis and synapse organization, are all contributing to synaptic dysfunction, neurotransmission, and neurodevelopment (Main Text Figure 6d).

Chapter 6

Evaluating cLD under different conditions

6.1 Sequencing depth

The main analysis in this work is based on the Phase 3 data of the 1000 Genomes Project, which has a low coverage of 4X per individual on average. However, with the increasing ability of genomic sequencing, deeper sequencing may reveal more rare-SNVs, which may alter the values of cLD and affect its applicability in measuring gene similarity. To investigate this potential concern, we compared the performance of cLD on the Phase 3 low-depth data and the newly released high-depth data of 30X coverage by the 1000 Genomes Project, by calculating the distributions of the number of rare variants per gene, cMAF per gene, and cLD patterns.

To assess the performance of cLD on high-depth data, we used the data released in 2022 to calculate the number of rare variants per gene, cMAF per gene, and to check cLD's pattern on Hi-C 3D gene-gene interactions. To facilitate fine-scale comparison, we standardized the raw counts of genes into probability distribution functions, displayed in Supplementary Figure K and Supplementary Figure L.

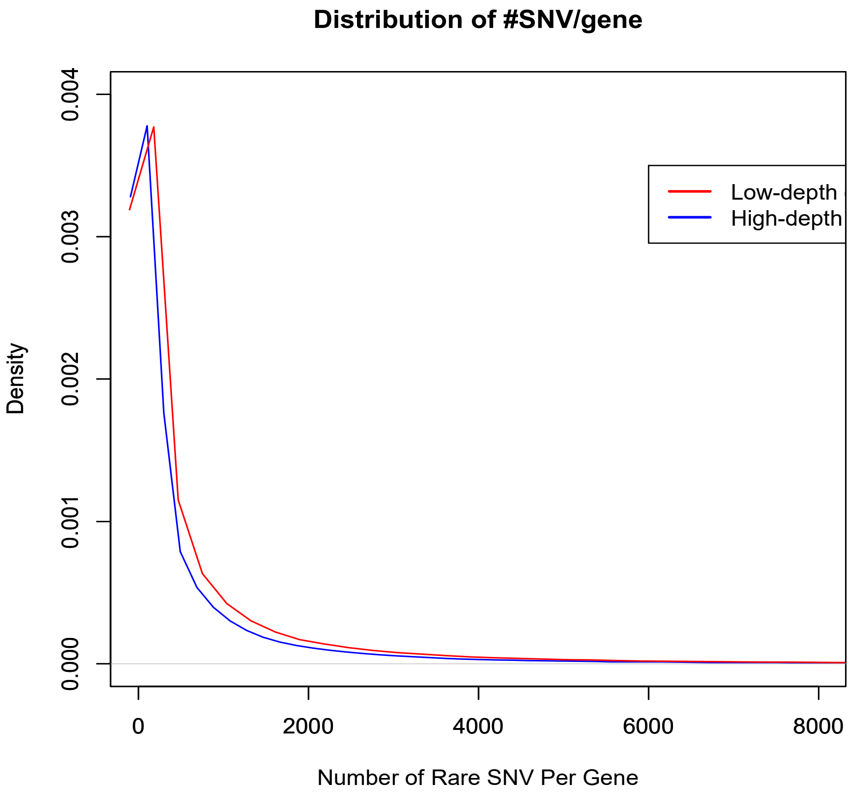

Fig. K: Probability Density Functions of Distributions of the Number of Rare SNVs per Gene in Low-Depth (Red) and High-Depth (Blue) Data. The median number of rare SNVs per gene in the low-depth data is 86, while in the high-depth data it is 94. Although there is a difference in the median values, the distribution of the number of rare SNVs per gene is not significantly different between the two datasets. The definition of rare SNV is MAF < 0.005.

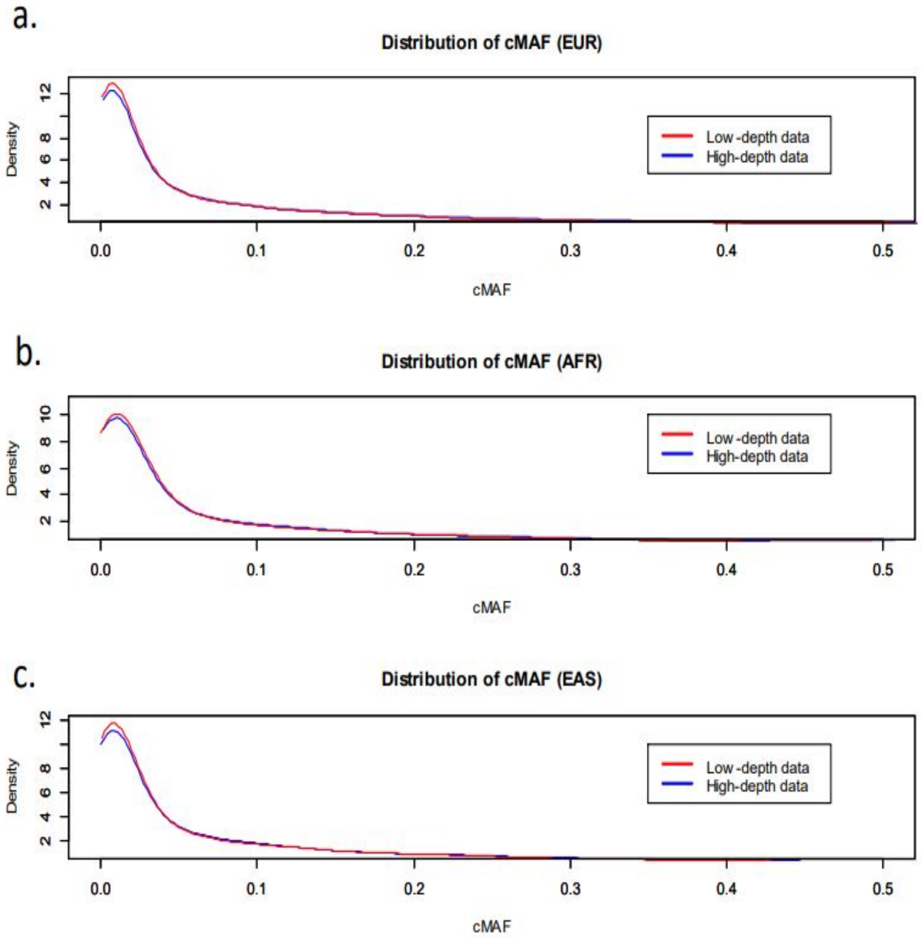

Fig. L: Probability Density Functions of Distributions of cMAF in Low-Depth (Red) and High-Depth (Blue) Data. (a) EUR population; (b) AFR population; (c) EAS population. The distributions of cMAF are very similar in the three populations, indicating that cLD performs similarly in low-depth and high-depth data. The cMAF calculation uses a standard cutoff of defining the rare variants as MAF < 0.005.

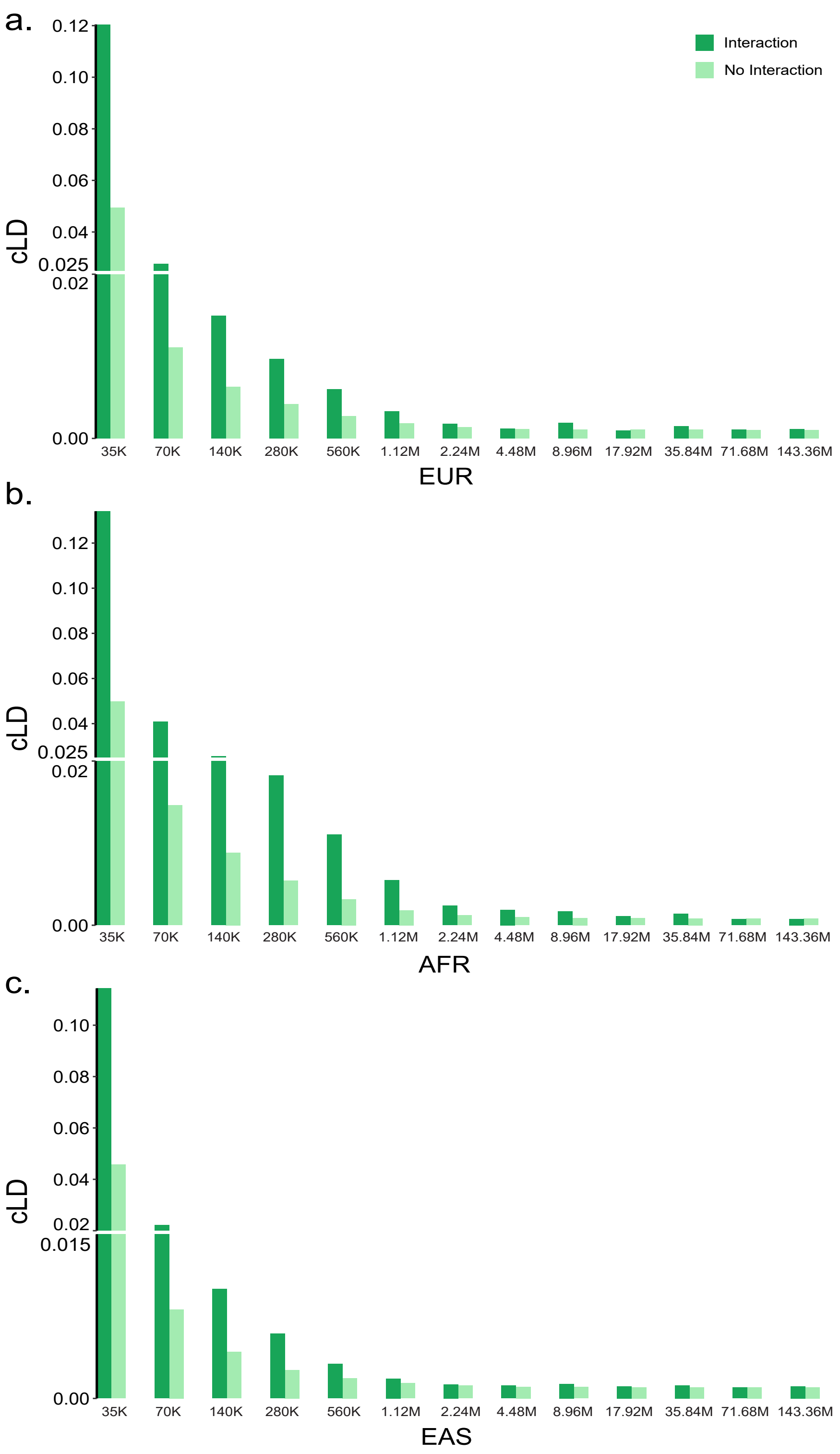

Fig. M: PThe comparisons of cLD values between the 3D chromatin interaction regions and non-interaction regions among 13 different distance groups using High-depth data. (a) EUR population, (b) AFR population, and (c) EAS population.

Our results demonstrated that the distributions of the number of rare variants per gene doesn't change significantly between low-depth data and high-depth data (Supplementary Figure K). Additionally, the rare-variants-based cMAF per gene also does not significantly differ between the two datasets (Supplementary Figure L). Finally, Supplementary Figure M shows that cLD has the same pattern observed in the main analysis using low-depth data. Our results showed that cLD is generally robust to the number of rare variants, as long as the total numbers are in a reasonable range, which can be ensured by selecting regions being a gene. Overall, our findings suggest that cLD is a reliable measure of gene similarity, even in high-depth data, and that it can be effectively applied to rare variant analysis.

6.2 Recombination and interaction

The decay of Linkage Disequilibrium (LD) between two loci across generations in proportion to the recombination rate is a documented phenomenon1. Similarly, the same phenomenon must be experienced by our statistic, cumulative Linkage Disequilibrium (cLD). To this end, we have conducted a series of simulation to show the decay of cLD with increasing recombination rates. Furthermore, we investigate the effects of genetic linkage on cLD when two genes have interactions between each other.

The simulations were conducted by the design and development of a software in C++. The simulator will analyse the variations in cLD between two genes A and B. The overview of the software architecture is showen in Supplementary Table O.

The simulation begins by formulating the genomic structure of an ideal population. The population has two genes (Gene A and Gene B). We evenly spread the mutations among these two genes for half the population. This creates a starting cLD value of 1. Effective population size of an equal number of males and females is assumed. In each generation, first, the parents are paired. Then the number of progeny produced by each parent pair is determined using random independent draws from a binomial distribution. Each progeny

Table O: List of parameters and their descriptions explaining their functionality.

Parameter	Description
Number of generations	The number of generations through which the simulations will have to run. Generations represent time.
Starting population size	The initial effective population size of the study. (It is assumed that a 50% male and female population size is maintained across generations).
Progeny generation distribution	The number of progenies a parent pair (Male and Female) will generate is determined by a binomial distribution.
Mutation rate	This takes the form of a Poisson distribution where the mean is essentially the rate/ or average number of mutations. Both genes A and B have the same mutation rate.
Variable sites	Number of regions in each gene susceptible to mutations. Both genes; gene A and gene B have equal number of variable sites.
Recombination probability	Probability that a recombination event will occur within gene's A, B.
Interactions	Whether the genes are in interaction with each other or not.

begins with a set fitness level, which comes in the form of their probability of survival for the next generation. To accommodate our negative selection model the wild type (No mutations in either gene A or gene B) is set to have an 80% chance of survival. Then the genetic configuration of each progeny's gene A and gene B is determined using a 50% probability draw deciding if it will have either the paternal or maternal genes. Once the progeny's genetic makeup for gene A and gene B is determined recombination is considered for the different mutation points. Afterward, each gene's variable sites are subjected to independent mutation events. Once mutation has occurred the effects of these mutations on the fitness of the progeny is assessed. Due to the negative selection model mutations have a negative effect on survival. This is accounted for by the reduction in the progeny's survivability parameter. However, in the presence of gene A and gene B interaction (that can be activated by the Interaction parameter above), if both gene regions have undergone mutation events, then fitness is increased above the wild type. Finally, the surviving progeny are determined, and based on their genetic makeup the cLD value of the surviving population in that generation is calculated. The surviving progeny are then made to parents and moved into the next

generation. The cycle continues till the generation span is completed.

Across the board of increasing recombination probabilities of 0.001, 0.005, 0.010 and 0.050 there was an increase in the rate of cLD decay conforming the correlation between the two factors. Furthermore, it was evident that rate of cLD decay was reduced in the presence of gene-gene interaction. In each instance the rate of cLD decay was higher in the absence of interaction in contrast to when it was present.

6.3 Switching error in haplotype inference

As phased data is the precondition to calculate cLD. The accuracy of haplotype phasing becomes paramount. It is widely known that rare variants are more prone to switch errors compared to common variants. Both our theoretical framework and bootstrap analysis hinge on the precision of haplotype phasing. Thus, it's imperative to assess how cLD performs when faced with inaccurate phasing results, especially in terms of switching errors. To address this concern, we conducted simulations of haplotype data, introducing switching errors modeled after those reported by Choi et al. (2018). Subsequently, we computed cLD using these simulated datasets. Below, we outline the detailed steps of this process.

Step 1: Randomly selected genes from the 1000 Genomes Project data.

Step 2: In the selected genes, we identified all sites susceptible to causing switching errors. Then, we employed a predefined Switching Error Rate (SER) ranging from 0.01 to 0.1.

Step 3: Afterward, we calculate the cLDs based on the original input of haplotypes and the cLDs based on the haplotypes with switching errors (Switching Error cLD).

The results are illustrated depicted in Main Text Figure 7b. As illustrated in the figure, the Switching Error cLD closely aligns with the true cLD, suggesting that switching errors exert only a marginal influence on cLD. This finding underscore the robustness of cLD.

Furthermore, we provide an example to further illustrate this resilience in Supplementary Figure N. In this example, there are 7 individuals and 7 SNVs in the gene. We intentionally introduced phasing errors in individuals 1, 2, and 3 at SNVs 6-7, 1-3, and 7, respectively. Although switching errors manifest at the SNV level, they do not alter the gene's sequence upon the integration. While this example may represent an ideal case, it effectively demonstrates the robust nature of cLD.

In summary, the cLD derived from the SNVs exhibits robustness against switching errors. Our findings demonstrate that despite potential alternations by switching errors, their impact on the cLD is remains minimal.

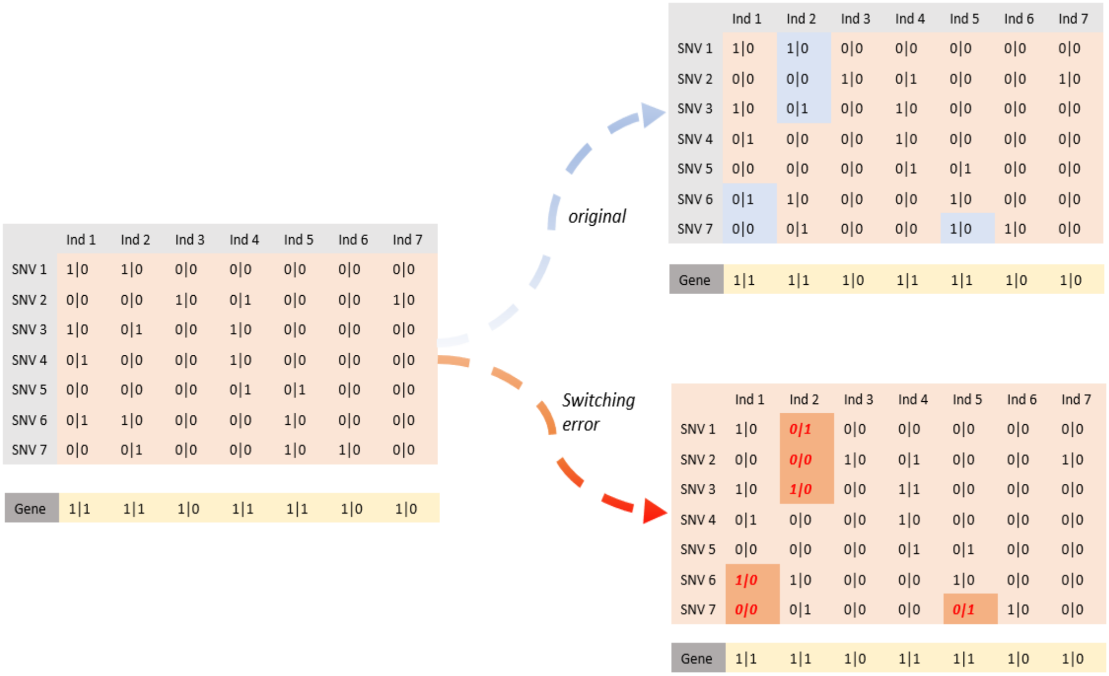

Fig. N: Illustration of the robustness of cLD, a) original SNVs b) simulated switching error. a) In the original data, we have 7 individuals and 7 SNVs in the gene. The gene line at the bottom is computed through integration across SNVs. b) We simulated phasing errors in individuals 1, 2, and 3 at SNVs 6-7, 1-3, and 7, respectively. However, after integration, the gene line remains unchanged. This example illustrates the robustness of the cLD against switching errors.

Bibliography

1. Clarke, L. *et al.* The international Genome sample resource (IGSR): A worldwide collection of genome variation incorporating the 1000 Genomes Project data. *Nucleic acids research* **45,** D854–D859 (2017).
2. Whalen, S. & Pollard, K. S. Most chromatin interactions are not in linkage disequilibrium. *Genome research* **29,** 334–343 (2019).
3. Rajarajan, P. *et al.* Neuron-specific signatures in the chromosomal connectome associated with schizophrenia risk. *Science* **362** (2018).
4. Stark, C. *et al.* BioGRID: a general repository for interaction datasets. *Nucleic acids research* **34,** D535–D539 (2006).
5. Fabregat, A. *et al.* The reactome pathway knowledgebase. *Nucleic acids research* **46,** D649–D655 (2018).
6. Licata, L. *et al.* MINT, the molecular interaction database: 2012 update. *Nucleic acids research* **40,** D857–D861 (2012).
7. Orchard, S. *et al.* The MIntAct project—IntAct as a common curation platform for 11 molecular interaction databases. *Nucleic acids research* **42,** D358–D363 (2014).
8. Berman, H. M. *et al.* The protein data bank. *Nucleic acids research* **28,** 235–242 (2000).
9. DeLano, W. L. *et al.* Pymol: An open-source molecular graphics tool. *CCP4 Newsletter on protein crystallography* **40,** 82–92 (2002).

10. Yan, Y., Tao, H., He, J. & Huang, S.-Y. The HDOCK server for integrated protein–protein docking. *Nature protocols* **15,** 1829–1852 (2020).

11. Yan, Y., Zhang, D., Zhou, P., Li, B. & Huang, S.-Y. HDOCK: a web server for protein–protein and protein–DNA/RNA docking based on a hybrid strategy. *Nucleic acids research* **45,** W365–W373 (2017).

12. Laskowski, R. A. & Swindells, M. B. *LigPlot+: multiple ligand–protein interaction diagrams for drug discovery* 2011.

13. Hamdan, F. F. *et al.* High rate of recurrent de novo mutations in developmental and epileptic encephalopathies. *The American Journal of Human Genetics* **101,** 664–685 (2017).

14. Keeling, K. M. *et al.* Gentamicin-mediated suppression of Hurler syndrome stop mutations restores a low level of α-L-iduronidase activity and reduces lysosomal glycosaminoglycan accumulation. *Human molecular genetics* **10,** 291–300 (2001).

15. Dwyer, C. A. & Esko, J. D. Glycan susceptibility factors in autism spectrum disorders. *Molecular aspects of medicine* **51,** 104–114 (2016).

16. Parikshak, N. N. *et al.* Genome-wide changes in lncRNA, splicing, and regional gene expression patterns in autism. *Nature* **540,** 423–427 (2016).

17. Tang, J., Yu, Y. & Yang, W. Long noncoding RNA and its contribution to autism spectrum disorders. *CNS Neuroscience & Therapeutics* **23,** 645–656 (2017).

18. Satterstrom, F. K. *et al.* Large-scale exome sequencing study implicates both developmental and functional changes in the neurobiology of autism. *Cell* **180,** 568–584 (2020).

19. Purcell, S. *et al.* PLINK: a tool set for whole-genome association and population-based linkage analyses. *The American journal of human genetics* **81,** 559–575 (2007).

20. Abrahams, B. S. *et al.* SFARI Gene 2.0: a community-driven knowledgebase for the autism spectrum disorders (ASDs). *Molecular autism* **4,** 1–3 (2013).

21. Piñero, J. *et al.* DisGeNET: a comprehensive platform integrating information on human disease-associated genes and variants. *Nucleic acids research,* gkw943 (2016).

22. Ashburner, M. *et al.* Gene ontology: tool for the unification of biology. *Nature genetics* **25,** 25–29 (2000).

23. Kanehisa, M., Goto, S., Furumichi, M., Tanabe, M. & Hirakawa, M. KEGG for representation and analysis of molecular networks involving diseases and drugs. *Nucleic acids research* **38,** D355–D360 (2010).

24. Kanehisa, M. & Goto, S. KEGG: kyoto encyclopedia of genes and genomes. *Nucleic acids research* **28,** 27–30 (2000).

25. Yu, G., Wang, L.-G., Han, Y. & He, Q.-Y. clusterProfiler: an R package for comparing biological themes among gene clusters. *Omics: a journal of integrative biology* **16,** 284–287 (2012).

26. Rojas, D. C. The role of glutamate and its receptors in autism and the use of glutamate receptor antagonists in treatment. *Journal of neural transmission* **121,** 891–905 (2014).

27. Hannelius, U. *et al.* Phenylketonuria screening registry as a resource for population genetic studies. *Journal of medical genetics* **42,** e60–e60 (2005).

28. Richler, E., Reichert, J. G., Buxbaum, J. D. & McInnes, L. A. Autism and ultraconserved non-coding sequence on chromosome 7q. *Psychiatric genetics* **16,** 19–23 (2006).

29. O'Roak, B. J. *et al.* Sporadic autism exomes reveal a highly interconnected protein network of de novo mutations. *Nature* **485,** 246–250 (2012).

30. Pittenger, C., Bloch, M. H. & Williams, K. Glutamate abnormalities in obsessive compulsive disorder: neurobiology, pathophysiology, and treatment. *Pharmacology & therapeutics* **132,** 314–332 (2011).

31. Sato, D. *et al.* SHANK1 deletions in males with autism spectrum disorder. *The American Journal of Human Genetics* **90,** 879–887 (2012).

32. Berkel, S. *et al.* Mutations in the SHANK2 synaptic scaffolding gene in autism spectrum disorder and mental retardation. *Nature genetics* **42,** 489–491 (2010).

33. Durand, C. M. *et al.* Mutations in the gene encoding the synaptic scaffolding protein SHANK3 are associated with autism spectrum disorders. *Nature genetics* **39,** 25–27 (2007).

34. Wei, H. *et al.* Genetic risk factors for autism-spectrum disorders: a systematic review based on systematic reviews and meta-analysis. *Journal of Neural Transmission,* 1–18 (2021).

35. Fung, L. K. & Hardan, A. Y. Developing medications targeting glutamatergic dysfunction in autism: progress to date. *CNS drugs* **29,** 453–463 (2015).

36. Vukojevic, V. *et al.* Evolutionary conserved role of neural cell adhesion molecule-1 in memory. *Translational psychiatry* **10,** 1–13 (2020).

37. Ye, H., Liu, J. & Wu, J. Y. Cell adhesion molecules and their involvement in autism spectrum disorder. *Neurosignals* **18,** 62–71 (2010).

38. Betancur, C., Sakurai, T. & Buxbaum, J. D. The emerging role of synaptic cell-adhesion pathways in the pathogenesis of autism spectrum disorders. *Trends in neurosciences* **32,** 402–412 (2009).

39. Lin, Y.-C., Frei, J. A., Kilander, M. B., Shen, W. & Blatt, G. J. A subset of autism-associated genes regulate the structural stability of neurons. *Frontiers in cellular neuroscience* **10,** 263 (2016).

Table P Avarge cLD and LD differences under different distance groups within HiC regions

Differences	35K	70K	140K	280K	560K	1.12M	2.25M	4.48M	8.96M	17.92M	35.84M	71.68M	143.36M
							ALL						
cLD	10.5267	1.412582	0.361306	0.196311	0.106955	0.055429	0.11014	0.122863	0.100957	0.0328643	0.0838113	-0.003591	0.02210003
LD	0.564555	0.350551	0.006865	0.144742	0.040328	0.014619	0.044832	0.045313	0.092325	0.000178	0.0066397	-0.012243	-0.0125472
							EUR						
cLD	10.00956	1.608974	0.513451	0.247604	0.119063	0.049415	0.02088	0.00275	0.044744	0.0535726	0.0227532	0.0057118	0.02287723
LD	0.366129	0.341472	-0.0189	0.189302	0.032054	0.011781	0.026079	0.044087	0.162707	-0.000339	0.0037259	-0.013862	-0.011128
							AFR						
cLD	9.163749	1.939288	1.039862	0.707526	0.40415	0.168362	0.113889	0.050919	0.083903	0.0136101	0.0331189	0.0121929	0.00365998
LD	0.746834	0.190576	0.155369	0.129529	-0.00596	0.074953	0.029822	0.113671	0.105091	0.002107	0.0206937	-0.008054	-0.0112917
							EAS						
cLD	11.18113	1.598825	0.405407	0.158421	0.040557	0.013149	0.008521	-0.01193	-0.00154	-0.033424	0.0165557	-0.016521	-0.0267565
LD	0.601583	0.384175	-0.05783	0.120554	0.038649	-0.01089	0.055776	0.038745	0.08395	0.0013229	0.0077724	-0.006828	-0.0087927

Notes:
Differences: The cLD differences between HiC regions and non-HiC regions, which have been nomalized by mean cLD values
cLD: The cLD differences for cLD
LD: The cLD differences for LD

Table Q The number of gene pairs above and below the cutoff (the 0.5 quantile), the ratio and the statistic tests between interaction and no interaction groups

	No Interaction			Interaction		
Distances	Above cutoff	Below cutoff	Above cutoff Ratio	Above cutoff 2	Below cutoff 2	Above cutoff Ratio 2
			ALL			
35K	47395	20986	0.693101885	322	24	0.930635838
70K	45487	19797	0.696755713	238	18	0.9296875
140K	82966	38740	0.681691946	319	28	0.919308357
280K	148807	78921	0.653441825	405	73	0.847280335
560K	260726	163947	0.613945318	545	101	0.843653251
1.12M	450540	335619	0.573090176	791	215	0.786282306
2.24M	778634	660850	0.540911882	1305	429	0.752595156
4.48M	1344590	1237730	0.520690697	1839	724	0.717518533
8.96M	2386065	2280260	0.51133708	3073	1441	0.680770935
17.92M	4121403	4071399	0.503051703	4849	2229	0.685080531
35.84M	6785310	6875148	0.49671175	7800	3656	0.680865922
71.68M	9749466	9995837	0.493761276	10610	5465	0.660031104
143.36M	11195129	11641018	0.49023721	11725	5697	0.672999656
* cLD cutoff: 0.000111915 (the 0.5 quantile)<br>* Fishers exact test: 93.8635251678832; p value=0<br>* Mantel-Haenszel test: 64.42275039084855; p value=0						
			EUR			
35K	34416	18932	0.645122591	287	58	0.831884058
70K	33330	19649	0.629117197	207	49	0.80859375
140K	60992	38843	0.610928031	283	64	0.81556196
280K	109762	78673	0.582492637	350	127	0.733752621
560K	196071	154126	0.55988772	479	163	0.746105919
1.12M	346889	301292	0.535173046	694	312	0.689860835
2.24M	612785	572459	0.517011687	1130	606	0.650921659
4.48M	1078875	1040769	0.508988774	1637	924	0.639203436
8.96M	1932531	1894550	0.504962137	2874	1635	0.637391883
17.92M	3353328	3334543	0.501404408	4360	2706	0.617039343
35.84M	5537627	5558323	0.499067407	7233	4202	0.632531701
71.68M	7959717	8032067	0.497737901	9973	6073	0.621525614
143.36M	9091958	9329494	0.493552734	10795	6611	0.620188441
* cLD cutoff: 0.00026251 (the 0.5 quantile)<br>* Fishers exact test: 66.4553; p value=0<br>* Mantel-Haenszel test: 45.9197852624032; p value=0						
			AFR			
35K	40555	17954	0.693141226	306	38	0.889534884
70K	39566	17510	0.693216063	229	25	0.901574803
140K	72922	34908	0.6762682	296	50	0.855491329
280K	131997	71342	0.649147483	389	87	0.817226891
560K	233316	145670	0.61563224	532	111	0.827371695
1.12M	405540	294440	0.57935941	805	199	0.801792829
2.24M	702784	579264	0.548172923	1209	525	0.697231834
4.48M	1210471	1088045	0.526631531	1663	897	0.649609375
8.96M	2136767	2018902	0.514181231	2819	1685	0.625888099
17.92M	3669897	3605032	0.504458119	4419	2641	0.62592068
35.84M	6017987	6087190	0.497141595	6907	4537	0.603547711
71.68M	8626914	8876426	0.492872446	9488	6548	0.591668745
143.36M	9863005	10330214	0.488431537	10436	6980	0.599219109
* cLD cutoff: 0.00025231 (the 0.5 quantile)<br>* Fishers exact test: 60.09742; p value=0<br>* Mantel-Haenszel test: 40.598032344146; p value=0						
			EAS			
35K	32761	21264	0.606404442	271	73	0.787790698
70K	30896	22578	0.577776115	184	72	0.71875
140K	56832	44328	0.561803084	225	121	0.650289017
280K	102992	87790	0.539841285	309	166	0.650526316
560K	186605	168505	0.525485061	433	210	0.67340591
1.12M	338369	317327	0.516045545	653	348	0.652347652
2.24M	611357	588333	0.509595812	1063	669	0.613741339
4.48M	1088772	1058901	0.506954271	1551	1002	0.607520564
8.96M	1955839	1911798	0.505693528	2776	1715	0.618125139
17.92M	3402179	3364836	0.502759193	4294	2758	0.608905275
35.84M	5623602	5610171	0.500597796	6927	4487	0.60688628
71.68M	8071457	8131260	0.498154538	9756	6256	0.60929303
143.36M	9264409	9460569	0.494762077	10775	6581	0.62082277
* cLD cutoff: 0.000266469 (the 0.5 quantile)<br>* Fishers exact test: 58.5469995; p value=0<br>* Mantel-Haenszel test:40.7590064170726; p value=0						

Notes:
Above cutoff: Number of gene pairs above cLD cutoff without interactions
Below cutoff: Number of gene pairs below cLD cutoff without interactions
Above cutoff 2: Number of gene pairs above cLD cutoff with interactions
Below cutoff 2: Number of gene pairs below cLD cutoff with interactions
Above cutoff Ratio: The ratio of number of gene pairs above cutoff

Table R The number of gene pairs above and below the cutoff (the 0.5 quantile), the ratio and the statistic tests within and without HiC regions

	without HiC regions			within HiC regions		
Distances	Above cutoff	Below cutoff	Above cutoff Ratio	Above cutoff 2	Below cutoff 2	Above cutoff Ratio 2
			ALL			
35K	47642	21004	0.694024415	75	7	0.914634146
70K	45516	19804	0.696815677	209	12	0.945701357
140K	82967	38725	0.681778589	318	44	0.878453039
280K	148833	78943	0.653418271	379	51	0.881395349
560K	260934	163980	0.614086615	337	68	0.832098765
1.12M	451231	335797	0.573335383	100	38	0.724637681
2.24M	779888	661248	0.541161972	51	31	0.62195122
4.48M	1346386	1238435	0.520881717	43	20	0.682539683
8.96M	2389097	2281671	0.511499822	41	30	0.577464789
17.92M	4126180	4073582	0.503207288	72	46	0.610169492
35.84M	6792988	6878739	0.496863929	122	65	0.652406417
71.68M	9759922	10001248	0.493893934	154	54	0.740384615
143.36M	11206696	11646643	0.490374558	158	72	0.686956522
* cLD cutoff: 0.000111915 (the 0.5 quantile)						
* Fishers exact test: 29.8470180815284; p value=0						
* Mantel-Haenszel test: 12.9902403222601; p value=0						
			EUR			
35K	34265	18955	0.643836903	438	35	0.926004228
70K	32283	19459	0.623922539	1254	239	0.839919625
140K	58711	38325	0.605043489	2564	582	0.815003179
280K	106839	78005	0.577995499	3273	795	0.804572271
560K	193842	153376	0.558271749	2708	913	0.747859707
1.12M	346274	300979	0.534990182	1309	625	0.676835574
2.24M	613401	572746	0.517137421	514	319	0.617046819
4.48M	1080278	1041540	0.509128493	234	153	0.604651163
8.96M	1935253	1896061	0.505114694	152	124	0.550724638
17.92M	3357560	3337154	0.501524038	128	96	0.571428571
35.84M	5544727	5562428	0.499203171	133	97	0.57826087
71.68M	7969521	8038019	0.497860446	169	121	0.582758621
143.36M	9102659	9336058	0.493670953	94	47	0.666666667
* cLD cutoff: 0.000262506562664066 (the 0.5 quantile)						
* Fishers exact test: 67.4754247635733; p value=0						
* Mantel-Haenszel test: 32.3985821294509; p value=0						
			AFR			
35K	40401	17969	0.692153504	460	23	0.952380952
70K	38420	17383	0.68849345	1375	152	0.900458415
140K	70317	34636	0.669985613	2901	322	0.900093081
280K	128719	70935	0.644710349	3667	494	0.881278539
560K	230713	145207	0.613728985	3135	574	0.845241305
1.12M	404843	294161	0.579171221	1502	478	0.758585859
2.24M	703402	579515	0.548283326	591	274	0.683236994
4.48M	1211872	1088808	0.526745136	262	134	0.661616162
8.96M	2139411	2020481	0.514294842	175	106	0.622775801
17.92M	3674175	3607570	0.504573423	141	104	0.575510204
35.84M	6024755	6091621	0.497240677	139	106	0.567346939
71.68M	8636230	8882844	0.492961557	172	130	0.569536424
143.36M	9873362	10337127	0.488526626	79	67	0.54109589
* cLD cutoff: 0.000252305184470428 (the 0.5 quantile)						
* Fishers exact test: 87.8060651574607; p value=0						
* Mantel-Haenszel test: 34.8171247072209; p value=0						
			EAS			
35K	32613	21280	0.605143525	419	57	0.880252101
70K	29924	22324	0.572730057	1156	326	0.780026991
140K	54716	43635	0.556333947	2341	814	0.74199683
280K	100348	86824	0.536127199	2953	1132	0.722888617
560K	184548	167590	0.524078628	2490	1125	0.68879668
1.12M	337818	316943	0.515940931	1204	732	0.621900826
2.24M	611919	588668	0.50968318	501	334	0.6
4.48M	1090117	1059726	0.507068191	206	177	0.537859008
8.96M	1958454	1913404	0.50581762	161	109	0.596296296
17.92M	3406355	3367476	0.502869794	118	119	0.497890295
35.84M	5630390	5614553	0.500704183	139	106	0.567346939
71.68M	8081045	8137388	0.498262995	168	128	0.567567568
143.36M	9275099	9467091	0.49487808	85	59	0.590277778
* cLD cutoff: 0.000266468855552274 (the 0.5 quantile)						
* Fishers exact test: 51.3452777917977; p value=0						
* Mantel-Haenszel test: 28.5230909069764; p value=0						

Notes:
Above cutoff: Number of gene pairs above cLD cuttoff out of HiC regions
Below cutoff: Number of gene pairs below cLD cuttoff out of HiC regions
Above cutoff 2: Number of gene pairs above cLD cutoff within HiC regions
Below cutoff 2: Number of gene pairs below cLD cutoff within HiC regions
Above cutoff Ratio: The ratio of number of gene pairs above cutoff

Table S Comparisons between the number of gene pairs with cLD values larger than the cutoffs in whole genome and within 3D regions (only on chromosome 2)

Cutoff	Above cutoff	Without interactions	Percentage of significance 1	Percentage of significance 2	Ratio
			EUR		
* Total number of gene pairs in total: 6053460					
* Total number of gene pairs within 3D regions: 1357					
**0.05**	6345	6201	0.10612	0.00105	101.2406
**0.1**	2262	2203	0.04348	0.00037	116.35452
**0.2**	708	680	0.02063	0.00012	176.42032
**0.3**	426	407	0.01401	0.0000704	198.96094
**0.4**	238	225	0.00958	0.0000393	243.66336
			AFR		
* Total number of gene pairs: 6648481					
* Total number of gene pairs within 3D regions: 3940					
**0.05**	3940	3803	0.0973	0.00059	164.18902
**0.1**	1293	1250	0.03054	0.00019	157.03256
**0.2**	461	440	0.01491	0.0000693	215.09888
**0.3**	271	225	0.01136	0.0000408	278.78568
**0.4**	164	149	0.01065	0.0000247	431.88472
			EAS		
* Total number of gene pairs: 6140760					
* Total number of gene pairs within 3D regions: 1377					
**0.05**	5748	5614	0.097313	0.000936	103.96238
**0.1**	2055	1996	0.042847	0.000335	128.0349
**0.2**	610	587	0.016703	0.0000993	168.14586
**0.3**	352	336	0.011619	0.0000573	202.70549
**0.4**	214	205	0.006536	0.0000348	187.54994

Notes:
Cutoff: cLD values
Above cutoff: Number of gene pairs with cLD > cutoff (significant gene pairs)
Without interactions: Number of significant gene pairs without interactions
Percentage of Significance 1: Percentage of significant gene pairs with 3D interactions
Percentage of significance 2: Percentage of significant gene pairs in whole genome
Ratio: Ratio between above two percentages

Table T The success, success rate and p-value of hypergeometric distribution probability for DisGeNet and SFARI database from top 200 to 2000 gene pairs

Top gene pairs	Success	Success rate	p-value
DisGeNet			
**Top 200 gene pairs**	34	0.098	$2.39 \times 10-5$
**Top 500 gene pairs**	69	0.0981	$6.71 \times 10-9$
**Top 1000 gene pairs**	110	0.0992	$1.13 \times 10-13$
**Top 1500 gene pairs**	148	0.1031	0
**Top 2000 gene pairs**	175	0.1034	0
SFARI			
**Top 200 gene pairs**	23	0.0671	0.043
**Top 500 gene pairs**	62	0.0881	$1.59 \times 10-6$
**Top 1000 gene pairs**	96	0.0866	$6.92 \times 10-9$
**Top 1500 gene pairs**	127	0.0884	$5.49 \times 10-12$
**Top 2000 gene pairs**	146	0.0862	$8.69 \times 10-13$

Notes:
Top gene pairs: Rank the cLD differences between case and control groups and selected from top gene pairs
Success: the number of identified genes reported in the corresponding database
Success rate: the ratio of these validated genes against all of the selected genes identified.
p-value: the p-value from the hypergeometric distribution probability test
